# Supplementary material for: Cohort profile: the multigeneration Respiratory Health in Northern Europe, Spain and Australia (RHINESSA) cohort
Source: BMJ Open. 2022 Jun 2;12(6):e059434. doi: 10.1136/bmjopen-2021-059434 (PMC9163543; doi:10.1136/bmjopen-2021-059434)
Supplement: Supplementary data [file bmjopen-2021-059434supp001.pdf]

SUPPLEMENTARY MATERIAL

Cohort profile: The multi-generation Respiratory Health in Northern Europe, Spain and Australia (RHINESSA) cohort

Supplementary Table 1. Children and adolescents investigated as part of the RHINESSA G3 study (the adult offspring cohort is the main focus of the paper and not presented here).

	Children (4-9 years)		Adolescents (10-17 years)	
	Questionnaire	Clinical	Questionnaire	Clinical
Aarhus			245	6
Bergen	105	44	571	124
Melbourne	13	0	73	11
Tartu			132	16

Supplementary Table 2A. Bergen all generations

	G1	G2 RHINE/ ECRHS parent	G2 other parent	G3 (all age groups)	G4 (all age groups)
Birth years	1918-65	1947-71	1935-88	1965-2012	1988-2008
Study years	2016-18	1991-2012	2016-18	2012-18	2019-21
Numbers, questionnaire study	1470	3452	910	2440	750
Numbers, clinical study	145	835	152	667	433

Supplementary Table 2B. More details about the Bergen 4th generation cohort

	Children (4-9 years)	Adolescents (10-17 years)	Adults (18+ years)	Total	*Response rate (of total)*
Invited, eligible	436	399	138	973	
Participated questionnaire	379	263	108	750	*77%*
Participated clinical	197	163	73	433	*45%*

SUPPLEMENTARY INFORMATION ON THE EUROPEAN COMMUNITY RESPIRATORY HEALTH SURVEY (ECRHS)

ECRHS I

Co-ordinating Centre (London): P. Burney, S. Chinn, C. Luczynska†, D. Jarvis, E. Lai.

Project Management Group: P. Burney (Project leader-UK), S. Chinn (UK), C. Luczynska† (UK), D. Jarvis (UK), P. Vermeire† (Antwerp), H. Kesteloot (Leuven), J. Bousquet (Montpellier), D. Nowak (Hamburg), J. Prichard† (Dublin), R. de Marco† (Verona), B. Rijcken (Groningen), J.M. Antó (Barcelona), J. Alves (Oporto), G. Boman (Uppsala), N. Nielsen (Copenhagen), P. Paoletti (Pisa).

Financial support: The following grants helped to fund the local studies. **Australia**: Asthma Foundation of Victoria, Allen and Hanbury's; **Belgium**: Belgian Science Policy Office, National Fund for Scientific Research; **Estonia:** Estonian Science Foundation, grant no 1088; **France:** Ministère de la Santé, Glaxo France, Institut Pneumologique d'Aquitaine, Contrat de Plan Etat-Région Languedoc-Rousillon, CNMATS, CNMRT (90MR/10, 91AF/6), Ministre Delegué de la Santé, RNSP; **Germany**: GSF, Bundesminister für Forschung und Technologie; **Italy:** Ministero dell'Università e della Ricerca Scientifica e Tecnologica, CNR, Regione Veneto grant RSF n. 381/05.93; **Norway:** Norwegian Research Council project no. 101422/310; **Spain:** Fondo de Investigación Sanitaria (#91/0016-060-05/E, 92/0319 and #93/0393), Hospital General de Albacete, Hospital General Juan Ramón Jiménez, Dirección Regional de Salud Pública (Consejería de Sanidad del Principado de Asturias), CIRIT (1997 SGR 00079) and Servicio Andaluz de Salud; **Sweden:** The Swedish Medical Research Council, the Swedish Heart Lung Foundation, the Swedish Association against Asthma and Allergy; **Switzerland:** Swiss national Science Foundation grant 4026-28099; **UK:** National Asthma Campaign, British Lung Foundation, Department of Health, South Thames Regional Health Authority.

Coordination: The co-ordination of this work was supported by the European Commission; the authors and participants are grateful to the late C. Baya and M. Hallen for their help during the study, and to K. Vuylsteek and the members of the COMAC for their support.

ECRHS II

Steering Committee: U. Ackermann-Liebrich (University of Basel, Switzerland); N. Kuenzli (University of Basel, and University of Southern California, Los Angeles, USA); J.M. Antó and J. Sunyer (Institut Municipal d' Investigació Médica (IMIM-IMAS), Universitat Pompeu Fabra (UPF), Spain); P. Burney (project leader), S Chinn, D. Jarvis, J. Knox and C. Luczynska (King's College London, UK); I. Cerveri (University of Pavia, Italy); R. de Marco† (University of Verona, Italy); T. Gislason (Iceland University Hospital, Iceland); J. Heinrich and M. Wjst (GSF–Institute of Epidemiology, Germany); C. Janson (Uppsala University, Sweden); B. Leynaert and F. Neukirch

(Institut National de la Santé et de la Recherche Medicale (INSERM), France); J. Schouten (University of Groningen, The Netherlands); C. Svanes (University of Bergen, Norway); P. Vermeire† (University of Antwerp, Belgium).

Principal Investigators and senior scientific teams: **Australia: Melbourne** (M. Abramson, E.H. Walters, J. Raven); **Belgium: South Antwerp and Antwerp City** (P. Vermeire, J. Weyler, M. van Sprundel, V. Nelen); **Estonia: Tartu** (R. Jõgi, A. Soon); **France: Paris** (F. Neukirch, B. Leynaert, R. Liard, M. Zureik), **Grenoble** (I. Pin, J. Ferran-Quentin), **Bordeaux** (A. Taytard, C. Raherison), **Montpellier** (J. Bousquet, P.J. Bousquet); **Germany: Erfurt** (J. Heinrich, M. Wjst, C. Frye, I. Meyer); **Iceland: Reykjavik** (T. Gislason, E. Bjornsson, D. Gislason, K.B. *Jörundsdóttir*); **Italy: Turin** (R. Bono, M. Bugiani, P. Piccioni, E. Caria, A. Carosso, E. Migliore, G. Castiglioni), **Verona** (R. de Marco†, G. Verlato, E. Zanolin, S. Accordini, A. Poli, V. Lo Cascio, M. Ferrari, I. Cazzoletti), **Pavia** (A. Marinoni, S. Villani, M. Ponzio, F. Frigerio, M. Comelli, M. Grassi, I. Cerveri, A. Corsico); **Norway: Bergen** (A. Gulsvik, E. Omenaas, C. Svanes, B. Laerum); **Spain: Albacete** (J. Martinez-Moratalla Rovira, E. Almar, M. Arévalo, C. Boix, G. González, J.M. Ignacio García, J. Solera, J. Damián), **Galdakao** (N. Muñozguren, J. Ramos, I. Urrutia, U. Aguirre), **Barcelona** (J.M. Antó, J. Sunyer, M. Kogevinas, J.P. Zock, X. Basagaña, A. Jaen, F. Burgos, C. Acosta), **Huelva** (J. Maldonado, A. Pereira, J.L. Sanchez), **Oviedo** (F. Payo, I. Huerta, A. de la Vega, L. Palenciano, J. Azofra, A. Cañada); **Sweden**: **Göteborg** (K. Toren, L. Lillienberg, A.C. Olin, B. Balder, A. Pfeifer-Nilsson, R. Sundberg), **Umea** (E. Norrman, M. Soderberg, K.A. Franklin, B. Lundback, B. Forsberg, L. Nystrom), **Uppsala** (C. Janson, G. Boman, D. Norback, G. Wieslander, M. Gunnbjornsdottir); **Switzerland: Basel** (N. Küenzli, B. Dibbert, M. Hazenkamp, M. Brutsche, U. Ackermann-Liebrich); **United Kingdom: Ipswich** (D. Jarvis, R. Hall, D. Seaton), **Norwich** (D. Jarvis, B. Harrison).
Financial Support: **Australia:** National Health and Medical Research Council; **Belgium: Antwerp:** Fund for Scientific Research (G.0402.00), University of Antwerp, Flemish Health Ministry; **Estonia: Tartu:** Estonian Science Foundation grant no 4350; **France: (All)** Programme Hospitalier de Recherche Clinique—Direction de la Recherche Clinique (DRC) de Grenoble 2000 number 2610, Ministry of Health, Ministère de l'Emploi et de la Solidarité, Direction Génerale de la Santé, Centre Hospitalier Universitaire (CHU) de Grenoble; **Bordeaux:** Institut Pneumologique d'Aquitaine; **Grenoble:** Comité des Maladies Respiratoires de l'Isere; **Montpellier:** Aventis (France), Direction Regionale des Affaires Sanitaires et Sociales Languedoc-Roussillon; **Paris:** Union Chimique Belge-Pharma (France), Aventis (France), Glaxo France; **Germany: Erfurt:** GSF—National Research Centre for Environment and Health, Deutsche Forschungsgemeinschaft (FR1526/1-1); **Hamburg:** GSF—National Research Centre for Environment and Health, Deutsche Forschungsgemeinschaft (MA 711/4-1); **Iceland: Reykjavik:** Icelandic Research Council, Icelandic University Hospital Fund; **Italy: Pavia:** GlaxoSmithKline Italy, Italian Ministry of University and Scientific and Technological Research (MURST), Local University Funding for Research 1998 and 1999; **Turin:** Azienda Sanitaria Locale 4 Regione Piemonte (Italy), Azienda Ospedaliera Centro Traumatologico Ospedaliero/Centro Traumatologico Ortopedico—Istituto Clinico Ortopedico Regina Maria Adelaide Regione Piemonte; **Verona:** Ministero dell'Università e della Ricerca Scientifica (MURST), Glaxo Wellcome spa; **Norway: Bergen:** Norwegian Research Council, Norwegian Asthma and Allergy Association, Glaxo Wellcome AS, Norway Research Fund; **Spain: Albacete:** Fondo de Investigaciones Sanitarias (97/0035-01, 99/0034-01, and 99/0034-02), Hospital Universitario de Albacete, Consejeria de Sanidad; **Barcelona:** Sociedad Espanola de Neumología y Cirugía Toracica,

Public Health Service (R01 HL62633-01), Fondo de Investigaciones Sanitarias (97/0035-01, 99/0034-01, and 99/0034-02), Consell Interdepartamental de Recerca i Innovació Tecnològica (1999SGR 00241), Instituto de Salud Carlos III, Red de Centros de Epidemiología y Salud Pública (C03/09), Red de Bases moleculares y fisiológicas de las Enfermedades Respiratorias (C03/011), Red de Grupos Infancia y Medio Ambiente (G03/176); **Huelva:** Fondo de Investigaciones Sanitarias (97/0035-01, 99/0034-01, and 99/0034-02); **Galdakao:** Basque Health Department; **Oviedo:** Fondo de Investigaciones Sanitarias (97/0035-02, 97/0035, 99/0034-01, 99/0034-02, 99/0034-04, 99/0034-06, 99/350, and 99/0034-07), European Commission (EU-PEAL PL01237), Generalitat de Catalunya (CIRIT 1999 SGR 00214), Hospital Universitario de Albacete, Sociedad Española de Neumología y Cirugía Torácica (SEPAR R01 HL62633-01), Red de Centros de Epidemiología y Salud Pública (C03/09), Red de Bases moleculares y fisiológicas de las Enfermedades Respiratorias (C03/011), Red de Grupos Infancia y Medio Ambiente (G03/176, 97/0035-01, 99/0034-01, and 99/0034-02); **Sweden: Göteborg, Umea, and Uppsala**: Swedish Heart Lung Foundation, Swedish Foundation for Health Care Sciences and Allergy Research, Swedish Asthma and Allergy Foundation, Swedish Cancer and Allergy Foundation, Swedish Council for Working Life and Social Research (FAS); **Switzerland: Basel:** Swiss National Science Foundation, Swiss Federal Office for Education and Science, Swiss National Accident Insurance Fund; **UK: Ipswich and Norwich**: Asthma UK (formerly known as National Asthma Campaign).

Cordination: The coordination of this work was supported by the European Commission, as part of their Quality of Life programme (QLK4-CT-1999-01237).

ECRHS III

Principal Investigators and senior scientific teams: **Australia: Melbourne** (M. Abramson, G. Benke, S. Dharmage, B. Thompson, S. Kaushik, M. Matheson). **Belgium: South Antwerp & Antwerp City** (J. Weyler, H. Bentouhami, V. Nelen). **Estonia: Tartu** (R. Jõgi, H.Orru). **France: Bordeaux** (C. Raherison, P.O. Girodet), **Grenoble** (I. Pin, V. Siroux, J. Ferran, J.L. Cracowski), **Montpellier** (P. Demoly, A. Bourdin, I. Vachier), **Paris** (B. Leynaert, D. Soussan, D. Courbon, C. Neukirch, L. Alavoine, X. Duval, I. Poirier). **Germany: Erfurt** (J. Heinrich, E. Becker, G. Woelke, O. Manuwald), **Hamburg** (H. Magnussen, D. Nowak, A-M. Kirsten). **Iceland: Reykjavik** (T. Gislason, B. Benediktsdottir, D. Gislason, E.S. Arnardottir, M. Clausen, G. Gudmundsson, L. Gudmundsdottir, H. Palsdottir, K. Olafsdottir, S. Sigmundsdottir, K. Bara-Jörundsdottir). **Italy**: **Pavia** (I. Cerveri, A. Corsico, A. Grosso, F. Albicini, E. Gini, E.M. Di Vincenzo, V. Ronzoni, S. Villani, F. Campanella, M. Gnesi, F. Manzoni, L. Rossi, O. Ferraro), **Turin** (M. Bugiani, R. Bono, P. Piccioni, R. Tassinari, V. Bellisario, G. Trucco), **Verona** (R. de Marco†, S. Accordini, L. Calciano, L. Cazzoletti, M. Ferrari, A.M. Fratta Pasini, F. Locatelli, P. Marchetti, A. Marcon, E. Montoli, G. Nguyen, M. Olivieri, C. Papadopoulou, C. Posenato, G. Pesce, P. Vallerio, G. Verlato, E. Zanolin). **Norway: Bergen** (C. Svanes, E. Omenaas, A. Johannessen, T. Skorge, F. Gomez Real). **Spain: Albacete** (J. Martinez-Moratalla Rovira, E. Almar, A. Mateos, S. García, A. Núñez, P. López, R. Sánchez, E. Mancebo), **Barcelona** (J.M. Antó, J.P. Zock, J. Garcia-Aymerich, M. Kogevinas, X. Basagaña, A.E. Carsin, F. Burgos, C. Sanjuas, S. Guerra, B. Jacquemin, P. Davdand), **Galdakao** (N. Muñozguren, I. Urrutia, U. Aguirre, S. Pascual), **Huelva** (J. Antonio Maldonado, A. Pereira, J. Luis

Sánchez, L. Palacios), **Oviedo** (F. Payo, I. Huerta, N. Sánchez, M. Fernández, B. Robles). **Sweden: Göteborg** (K. Torén, M. Holm, J-L. Kim, A-C. Olin, A. Dahlman-Höglund), **Umea** (B. Forsberg, L. Braback, L. Modig, B. Järvholm, H. Bertilsson, K.A. Franklin, C. Wahlgreen), **Uppsala** (B. Andersson, D. Norback, U. Spetz Nystrom, G. Wieslander, G.M. Bodinaa Lund, K. Nisser). **Switzerland: Basel** (N.M. Probst-Hensch, N. Künzli, D. Stolz, C. Schindler, T. Rochat, J.M. Gaspoz, E. Zemp Stutz, M. Adam, C. Autenrieth, I. Curjuric, J. Dratva, A. Di Pasquale, R. Ducret-Stich, E. Fischer, L. Grize, A. Hensel, D. Keidel, A. Kumar, M. Imboden, N. Maire, A. Mehta, H. Phuleria, M. Ragettli, M. Ritter, E. Schaffner, G.A. Thun, A. Ineichen, T. Schikowski, M. Tarantino, M. Tsai). **UK: London** (P. Burney, D. Jarvis, S. Kapur, R. Newson, J. Potts), **Ipswich** (N. Innes), **Norwich** (A. Wilson).

Financial Support: **Australia:** National Health & Medical Research Council; **Belgium: Antwerp South, Antwerp City:** Research Foundation Flanders (FWO) (G.0.410.08.N.10); **Estonia: Tartu:** Estonian Ministry of Education (SF0180060s09); **France:** (**All centres**) Ministère de la Santé, Programme Hospitalier de Recherche Clinique (PHRC) national 2010; **Bordeaux:** INSERM U897 Université Bordeaux segalen; **Grenoble:** Comite Scientifique AGIRadom 2011; **Paris:** Agence Nationale de la Santé, Région Ile de France, domaine d'intérêt majeur (DIM); **Germany: Erfurt:** German Research Foundation (HE 3294/10-1); **Hamburg:** German Research Foundation (MA 711/6-1, NO 262/7-1); **Iceland: Reykjavik:** The Landspitali University Hospital Research Fund, University of Iceland Research Fund, ResMed Foundation, California, USA, Orkuveita Reykjavikur (Geothermal plant), Vegagerðin (The Icelandic Road Administration (ICERA). **Italy: (All centres)** Italian Ministry of Health, Chiesi Farmaceutici SpA; **Verona:** Cariverona foundation, Education Ministry (MIUR). **Norway:** Norwegian Research council (214123), Western Norway Regional Health Authorities (911631), Bergen Medical Research Foundation. **Spain:** Fondo de Investigación Sanitaria (PS09/02457, PS09/00716, 09/01511, PS09/02185, PS09/03190), Servicio Andaluz de Salud, Sociedad Española de Neumología y Cirurgía Torácica (SEPAR 1001/2010), Fondo de Investigación Sanitaria (PS09/02457); **Barcelona:** Fondo de Investigación Sanitaria (FIS PS09/00716); **Galdakao:** Fondo de Investigación Sanitaria (FIS 09/01511); **Huelva:** Fondo de Investigación Sanitaria (FIS PS09/02185), Servicio Andaluz de Salud; **Oviedo:** Fondo de Investigación Sanitaria (FIS PS09/03190). **Sweden: (All centres)** The Swedish Heart and Lung Foundation, The Swedish Asthma and Allergy Association, The Swedish Association against Lung and Heart Disease, Swedish Research Council for health, working life and welfare (FORTE); **Göteborg:** Swedish Council for Working life and Social Research; **Umea:** Vasterbotten Country Council ALF grant. **Switzerland:** The Swiss National Science Foundation (33CSCO-134276/1, 33CSCO-108796, 3247BO-104283, 3247BO-104288, 3247BO-104284, 3247-065896, 3100-059302, 3200-052720, 3200-042532, 4026-028099), The Federal office for forest, environment and landscape, The Federal Office of Public Health, The Federal Office of Roads and Transport, the canton's government of Aargan, Basel-Stadt, Basel-Land, Geneva, Luzern, Ticino, Valais, and Zürich, the Swiss Lung League, the canton's Lung League of Basel Stadt/Basel, Landschaft, Geneva, Ticino, Valais, and Zurich, SUVA, Freiwillige Akademische Gesellschaft, UBS Wealth Foundation, Talecris Biotherapeutics GmbH, Abbott Diagnostics, European Commission (018996) (GABRIEL), Wellcome Trust (WT 084703MA). **UK:** Medical Research Council (92091). Support also provided by the National Institute for Health Research through the Primary Care Research Network.

Coordination: The coordination was funded through the Medical Research Council (92091).

† deceased

Ethics approval: Ethics approval was obtained by all centres from the appropriate ethics committees: **Antwerp City and Antwerp South:** Adviescommissie Medische Ethiek UZA-UA (CME); **Tartu:** Research Ethics Committee of the University of Tartu, Estland (N° 209T-17); **French centres:** Comite de protection des personnes, Sud V Est (N° 2011-A00013-38); **German centres:** Ethik-Kommission der Bayerischen Landesarztekammer (N° 10015); **Reykjavik:** National Biotecs Committe of Iceland (NBCI) (N° VSNb2011090016/03.11); **Pavia:** Fondazione IRCCS Policlinico 'San Matteo' (N° P-20110024215); **Turin:** Comitato Etico dell'Azienda Sanitaria Locale TO/2 di Torino (N° 569/09/08); **Verona:** Comitato Etico per la Sperimentazione dell'Azienda Ospedaliera Istituti Ospitalieri di Verona (N° 1393); **Bergen:** Universitetet i Bergen, Regional komité for medisinsk og helsefaglig forskningsetikk, Vest-Norge (REK Vest) (N° 2010/759); **Albacete:** Comité de Ética e Investigación de Complejo Hospitalario de Albacete (N° 04/09); **Barcelona:** Comité Ético de Investigación Clínica del Instituto Municipal de Asistencia Sanitaria, Barcelona, Spain (N° PS09/00716); **Galdakao:** Comité Éticode Investigación del Hospital de Galdakao, Spain (N° 20101104); **Huelva:** Comisión de Investigación del Hospital Juan Ramón Jiménez de Huelva (N° 20090417); **Oviedo:** Comité Ético de Investigación Clínica Regional, Hospital Universitario Central de Asturias (N° 20110415); **Swedish centres:** Ethics Committee at the Medical Faculty, Uppsala University (N° 1999/313 and 2010/068); **Basel:** Swiss Academy of Medical Sciences and the ethics committee of Basel (N° PV123/00,157/00); **UK centres:** NRES Committee London - Stanmore (REC Reference 11/LO/0965 IRAS number 70769).
