# Supplementary figures and images for: Cohort profile: the multigeneration Respiratory Health in Northern Europe, Spain and Australia (RHINESSA) cohort
Source: BMJ Open. 2022 Jun 2;12(6):e059434. doi: 10.1136/bmjopen-2021-059434 (PMC9163543; doi:10.1136/bmjopen-2021-059434)

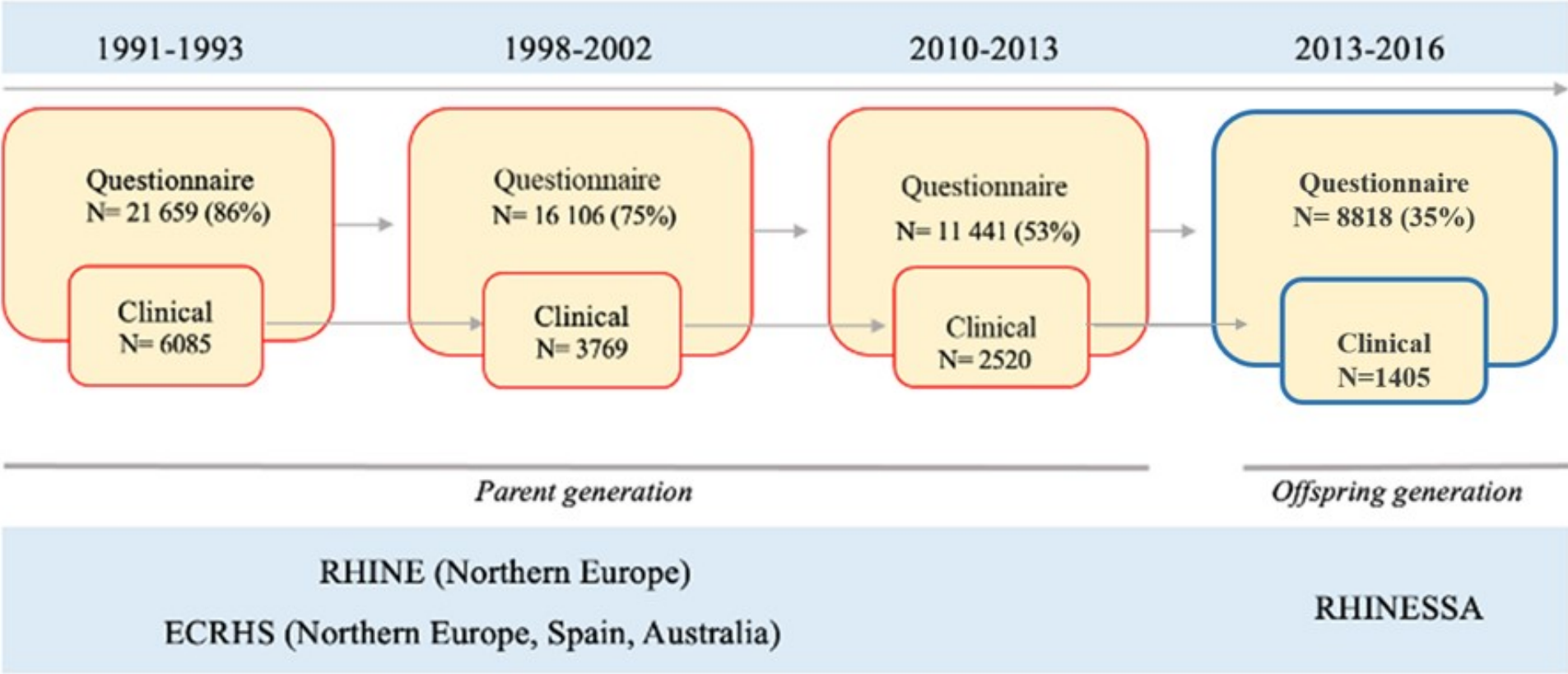

Supplement: Supplementary data [file bmjopen-2021-059434supp002.pdf]
