# Supplementary material for: Cohort profile: the multigeneration Respiratory Health in Northern Europe, Spain and Australia (RHINESSA) cohort
Source: BMJ Open. 2022 Jun 2;12(6):e059434. doi: 10.1136/bmjopen-2021-059434 (PMC9163543; doi:10.1136/bmjopen-2021-059434)
Supplement: Author's manuscript [file bmjopen-2021-059434.draft_revisions.pdf]

BMJ Open is committed to open peer review. As part of this commitment we make the peer review history of every article we publish publicly available.

When an article is published we post the peer reviewers' comments and the authors' responses online. We also post the versions of the paper that were used during peer review. These are the versions that the peer review comments apply to.

The versions of the paper that follow are the versions that were submitted during the peer review process. They are not the versions of record or the final published versions. They should not be cited or distributed as the published version of this manuscript.

BMJ Open is an open access journal and the full, final, typeset and author-corrected version of record of the manuscript is available on our site with no access controls, subscription charges or pay-per-view fees (http://bmjopen.bmj.com).

If you have any questions on BMJ Open's open peer review process please email info.bmjopen@bmj.com

BMJ Open

Cohort profile: The multi-generation Respiratory Health in Northern Europe, Spain and Australia (RHINESSA) cohort

Journal:	*BMJ Open*
Manuscript ID	bmjopen-2021-059434
Article Type:	Cohort profile
Date Submitted by the Author:	19-Nov-2021
Complete List of Authors:	Svanes, C; University of Bergen, Department of Global Public Health and Primary Care; Haukeland University Hospital, Department of Occupational Medicine<br>Johannessen, Ane; University of Bergen Department of Global Public Health and Primary Care, Centre for International Health<br>Bertelsen, Randi; University of Bergen, Department of Clinical Science; Oral Helath Centre of Expertise Western Norway<br>Dharmage, Shyamali; University of Melbourne, Allergy and Health Unit, School of Population and Global Health<br>Benediktsdottir, Bryndis; University of Iceland, Medical Faculty; Landspitali University Hospital Reykjavík, Department of Sleep<br>Bråbäck, Lennart; Umeå Universitet, Department of Public Health and Clinical Medicine<br>Gislason, Thorarinn; Landspitali University Hospital; Landspitali University Hospital Reykjavík, Department of Sleep<br>Holm, Mathias; University of Gothenburg, Occupational and Environmental Medicine, School of Public Health and Community Medicine, Institute of Medicine, Sahlgrenska Academy<br>Jõgi, Oskar; University of Bergen, Department of Clinical Science; Tartu University Hospital, Lung Clinic<br>Lodge, Caroline J.; University of Melbourne, Allergy and Lung Health Unit, School of Population and Global Health<br>Malinovschi, Andrei; Uppsala University, Department of Medical Sciences: Clinical Physiology<br>Martinez-Moratalla, Jesus; Complejo Hospitalario Universitario de Albacete, Servicio de Neumología; Universidad de Castilla-La Mancha - Campus de Albacete, Facultad de Medicina<br>Oudin, Anna; Umeå Universitet, Section of Sustainable Health, Department of Public Health and Clinical Medicine<br>Sánchez-Ramos, José Luis; University of Huelva, Department of Nursing<br>Timm, Signe; University of Southern Denmark, Department of Regional Health Research; University Hospital of Southern Denmark, Research Unit, Kolding Hospital<br>Janson, Christer; Uppsala University, Department of Medical Sciences: Clinical Physiology; Uppsala University, Department of Medical Sciences: Respiratory, Allergy, Sleep Research<br>Real, Francisco Gomez; University of Bergen, Department of Clinical Science; Haukeland University Hospital, Department of Obstetrics and Gynecology<br>Schlünssen, Vivi; Aarhus Universitet, Department of Public Health - Work, Environment and Health, Danish Ramazzini Centre; National

	Research Centre for the Working Environment
Keywords:	Asthma < THORACIC MEDICINE, Allergy < THORACIC MEDICINE, Chronic airways disease < THORACIC MEDICINE, EPIDEMIOLOGY, PUBLIC HEALTH

SCHOLARONE™
Manuscripts

I, the Submitting Author has the right to grant and does grant on behalf of all authors of the Work (as defined in the below author licence), an exclusive licence and/or a non-exclusive licence for contributions from authors who are: i) UK Crown employees; ii) where BMJ has agreed a CC-BY licence shall apply, and/or iii) in accordance with the terms applicable for US Federal Government officers or employees acting as part of their official duties; on a worldwide, perpetual, irrevocable, royalty-free basis to BMJ Publishing Group Ltd ("BMJ") its licensees and where the relevant Journal is co-owned by BMJ to the co-owners of the Journal, to publish the Work in this journal and any other BMJ products and to exploit all rights, as set out in our licence.

The Submitting Author accepts and understands that any supply made under these terms is made by BMJ to the Submitting Author unless you are acting as an employee on behalf of your employer or a postgraduate student of an affiliated institution which is paying any applicable article publishing charge ("APC") for Open Access articles. Where the Submitting Author wishes to make the Work available on an Open Access basis (and intends to pay the relevant APC), the terms of reuse of such Open Access shall be governed by a Creative Commons licence – details of these licences and which Creative Commons licence will apply to this Work are set out in our licence referred to above.

Other than as permitted in any relevant BMJ Author's Self Archiving Policies, I confirm this Work has not been accepted for publication elsewhere, is not being considered for publication elsewhere and does not duplicate material already published. I confirm all authors consent to publication of this Work and authorise the granting of this licence.

For peer review only

Title

Cohort profile: The multi-generation Respiratory Health in Northern Europe, Spain and Australia (RHINESSA) cohort

Authors

Svanes C[1,2*], Johannessen A[1]*, Bertelsen RJ[3,4], Dharmage SC[5], Benediktsdottir B [6,7], Bråbäck L[8], Gíslason T[6,7], Holm M[9], Jõgi NO[3, 10], Lodge C[5], Malinovschi A[11], Martinez-Moratalla J [12,13], Oudin A[8], Sanchez JL[14,], Timm S [15,16], Janson C[11,17], Gómez Real F[3,18]†, Schlünssen V[19,20]†.

* Shared first authors † Shared last authors

[1] Centre for International Health, Department of Global Public Health and Primary Care, University of Bergen, Bergen, Norway

[2] Department of Occupational Medicine, Haukeland University Hospital, Bergen, Norway

[3] Department of Clinical Science, University of Bergen, Bergen, Norway

[4] Oral Health Centre of Expertise Western Norway, Bergen, Norway

[5] Allergy and Lung Health Unit, School of Population and Global Health, University of Melbourne, Australia

[6] Medical Faculty, University of Iceland, Iceland

[7] Department of Sleep, Landspitali University Hospital Reykjavík, Iceland

[8] Section of Sustainable Health, Department of Public Health and Clinical Medicine, Umeå University, Sweden

[9] Occupational and Environmental Medicine, School of Public Health and Community Medicine, Institute of Medicine, Sahlgrenska Academy, University of Gothenburg, Gothenburg, Sweden

[10] Tartu Universtity Hospital, Lung Clinic, Tartu, Estonia

[11] Department of Medical Sciences: Clinical Physiology, Uppsala University, Uppsala, Sweden

For peer review only - http://bmjopen.bmj.com/site/about/guidelines.xhtml

[12] Servicio de Neumología del Complejo Hospitalario Universitario de Albacete, Albacete, Spain.

[13] Facultad de Medicina de Albacate. Universidad de Castilla – La Mancha. Albacete, Spain.

[14] Department of Nursing, University of Huelva, Huelva, Spain

[15] Department of Regional Health Research, University of Southern Denmark, 5230 Odense, Denmark;

[16] Research Unit, Kolding Hospital, University Hospital of Southern Denmark, 6000 Kolding, Denmark

[17] Department of Medical Sciences: Respiratory, Allergy, Sleep Research, Uppsala university, Uppsala, Sweden

[18] Department of Obstetrics and Gynecology, Haukeland University Hospital, Bergen, Norway

[19] Department of Public Health - Work, Environment and Health, Danish Ramazzini Centre, Aarhus University, Denmark

[20] National Research Centre for the Working Environment, Copenhagen, Denmark

Correspondence

Professor Cecilie Svanes, Centre for International Health, Overlege Danielsens Hus, Aarstadveien 21, University of Bergen, 5009 Bergen, Norway.

Telephone +4790892762. E-mail Cecilie.Svanes@uib.no

Word count 3626 words

Key words: RHINESSA; generation study; preconception factors; asthma; allergy; lung function; overweight; environment; occupation; smoking; puberty; non-genetic heredity; anthropometry; chronic diseases

For peer review only

ABSTRACT

Purpose: The Respiratory Health in Northern Europe, Spain and Australia (RHINESSA) cohort was established to investigate how exposures before conception, including in previous generations, influence health and disease, particularly allergies and respiratory health, to identify susceptible time windows, and to explore underlying mechanisms. The ultimate aim is to facilitate efficient intervention strategies targeting multiple generations.

Participants: The RHINESSA cohort includes adult offspring of ECRHS/RHINE study participants from ten study centres in Norway, Denmark, Sweden, Iceland, Estonia, Spain and Australia. The baseline investigation in 2014-17 included a questionnaire study (N=8818, age 18-53 years) and a clinical study of a subsample (n=1405). Data from one parent was obtained from the population-based multi-centre ECRHS and RHINE cohorts, which have followed women and men aged 20-44 years with 8-10 years intervals since the early 1990s. Study protocols for offspring were harmonised with those of the parents.

Findings to date: The collected data include spirometry, skin prick tests, exhaled nitric oxide, anthropometrics, bioimpedance, blood pressure; questionnaire/interview data on respiratory/general/reproductive health, indoor/outdoor environment, smoking, occupation, general characteristics and lifestyle; biobanked blood, urine, gingival fluid, skin swabs; and measured specific and total IgE, DNA methylation, sex hormones, oral microbiome, and more.

The results so far suggest 1) parental environment years before conception is important for asthma and lung function, 2) father's exposures such as smoking and overweight may be of key importance together with mother's exposures, and 3) there is an important susceptibility window in male prepuberty. Statistical analyses developed to approach causal inference suggest that these associations may be causal. DNA methylation studies suggest a mechanism for transfer of father's exposures to offspring health and disease through impact on offspring DNA methylation.

For peer review only - http://bmjopen.bmj.com/site/about/guidelines.xhtml

Future plans: Follow-up is planned at 5-8 year intervals, first in 2021-22. Linkage with health registries contributes to follow-up of the cohort.

Strengths and limitations of this study

- The main strength of the RHINESSA cohort is the availability of rich preconception exposure information for a large number of young adult study participants, both from the male and female line. Fathers and mothers have been extensively characterised over twenty years of childbearing age, providing important parental exposure information for adolescent and adult offspring.
- Excellent health and population registries in the Northern European study centres contribute to unbiased identification of study participants and enrichment of data. Some study centres have information on more generations covering cohorts born over the last century.
- The multi-generation design gives the possibility to validate next of kin information, thereby improving the value of retrospectively collected data on family members.
- The multi-centre study design is a strength; while the majority of study participants are from Northern Europe, the Spanish and Australian study centres contribute to wider generalisability. On the other hand, generalisation to low-income countries must be done with care.
- A weakness of RHINESSA is the relatively low response rates. While the availability of parental data for responders and non-responders allows for analyses of selection, potential for attrition to influence the study findings cannot be completely ruled out.
- A further weakness is that extensive exposure data are only directly available from one parent and this may lead to some misclassification of the preconception environment. Information on the other parent is available from (validated) data reported by the offspring, from a substudy of this cohort and from registry data.

For peer review only - http://bmjopen.bmj.com/site/about/guidelines.xhtml

INTRODUCTION

RHINESSA (**R**espiratory **H**ealth in **N**orthern **E**urope, **S**pain and **A**ustralia) is an international multi-generation multi-centre study for research on health and disease, in particular allergies and respiratory health. The RHINESSA study was set up to investigate the influence of exposures before conception including in previous generations for health and disease, to identify potentially susceptible time windows for such influences, and to explore mechanisms for exposure effects. RHINESSA's primary focus is allergies and chronic respiratory disorders, namely asthma, rhino-conjunctivitis, allergic sensitization, eczema, chronic obstructive pulmonary disease (COPD), lung function and sleep disorders. To be noted, the cohort resource and research methodologies of RHINESSA have the capacity for multi-generation research in other areas, such as obesity, women's health and oral health.

While it is generally acknowledged that intrauterine life is essential to health and disease throughout life, emerging evidence supports that there may be important susceptibility windows before conception[1-7]. This is the rationale behind RHINESSA, to explore how factors in time windows *before* conception may be of importance, for health and disease in general, and for respiratory health and allergies in particular. The hypothesis arose from new understanding of epigenetic mechanisms by which environmental effects could be transferred across generations[2, 8-10] and from studies supporting that such transfer of environmental effects (not causing mutations) across generations could actually be taking place in humans[11-13]. Theoretically, an exposure affecting one person might at the same time affect that person's germ cells, and thereby the health of future offspring (figure 1). The intrauterine period and male puberty may be time windows when the germ cells are more susceptible to external and internal factors due to extensive epigenetic reprogramming[2, 6, 14].

For peer review only - http://bmjopen.bmj.com/site/about/guidelines.xhtml

The concept of susceptible time windows opens opportunities for preventive measures resulting in improved health, not only of the individual but also for future generations[15], and thereby a new perspective on public health strategies. Long-term perspectives, such as strategies aimed at reducing cancer decades later, are fundamental in health policies. However, the perspective that a health intervention might benefit multiple generations over a similar time frame, is hardly acknowledged. The ultimate aim of RHINESSA is to improve health at large by generating a knowledge base for efficient strategies that may improve health over several generations.

As the human reproductive cycle spans decades, investigating exposure effects from before conception and across generations in humans represents a great challenge, and there are few cohorts that have the needed data. The RHINESSA study is designed to address this by investigating the offspring of persons who have already been extensively characterized during twenty years of their reproductive life. RHINESSA builds on the large longitudinal studies of respiratory health in adults, the European Community Respiratory Health Survey (ECRHS, www.ecrhs.org) established in the early 1990's[16-18] and the linked study, Respiratory Health in Northern Europe (RHINE, www.rhine.nu)[19, 20]. For a range of environmental exposures and lifestyle factors, the ECRHS and RHINE cohorts have data with high time-resolution, both before and during the age of childbearing. The children born to these parents, are the target population of RHINESSA. Northern Europe is well suited for generation studies due to excellent national registries with full coverage of the populations, providing means to identify family members in an unbiased manner as well as information on exposures and outcomes (i.e. life-time home addresses for geo-coding, prescription registries for asthma medication). Study centres in Estonia with Baltic diet and recent transition from middle- to high-income economy, Spain with Mediterranean characteristics, and Australia with particularly high allergy prevalence, contribute to greater generalisability of study results.

For peer review only - http://bmjopen.bmj.com/site/about/guidelines.xhtml

This cohort profile describes the RHINESSA adult offspring cohort (≥18 years) and their parents investigated as part of the ECRHS and RHINE studies (figure 2). The on-line supplement gives summary data for younger offspring and additional cohorts investigated in some study centres - altogether four generations.

COHORT DESCRIPTION

Study design

The RHINESSA adult offspring study invited offspring age ≥18 years of RHINE and ECRHS participants from ten study centres: Bergen, Norway; Aarhus, Denmark; Uppsala, Göteborg and Umeå, Sweden; Reykjavik, Iceland; Tartu, Estonia; Melbourne, Australia; Huelva and Albacete, Spain (table 1, figure 2). In the Northern European countries all offspring were identified through national registries, for the Spanish and Australian study centres the offspring's contact details were obtained from the parents in ECRHS III (table 1). All offspring with parental questionnaire information (from RHINE or ECRHS) were invited to a questionnaire study. The subsample of these with parental clinical information (from ECRHS) and residing in the study area, were invited to a clinical study. The baseline data collection was performed in all study centres during 2014-17. The study centres in Bergen, Aarhus, Tartu and Melbourne also investigated younger offspring <18 years (table S1). Bergen RHINESSA also investigated the grandparent generation, the parent not participating in RHINE/ECRHS, and the offspring's offspring. Summary data for these additional cohorts are given in the online supplement (table S2).

For peer review only - http://bmjopen.bmj.com/site/about/guidelines.xhtml

Table 1. Sources of selection / identification of RHINESSA adult participants (18+ years) and their parents, by centre, including a questionnaire cohort (8818 offspring with their 6441 parents), and a clinical cohort (1405 offspring with their 1041 parents).

	Parents		RHINESSA adult offspring	
Study centre	Source used for selecting offspring	N	Source of identification	N included in cohort
**QUESTIONNAIRE COHORT**				
Norway, Bergen	ECRHS I quest. respondents	1250	National registers	1763
Denmark, Aarhus	ECRHS I quest. respondents	974	National registers	1224
Sweden, Uppsala	RHINE III quest. respondents	894	National registers	1314
Sweden, Göteborg	RHINE III quest. respondents	709	National registers	951
Sweden, Umeå	RHINE III quest. respondents	876	National registers	1307
Iceland, Reykjavik	ECRHS I quest. respondents	977	National registers	1245
Estonia, Tartu	ECRHS I quest. respondents	525	National registers	618
Australia, Melbourne	ECRHS III clin. respondents	149	Through the parents	245*
Spain, Huelva	ECRHS III clin. respondents	48	Through the parents	72*
Spain, Albacete	ECRHS III clin. respondents	39	Through the parents	79*
**CLINICAL COHORT**				
Norway, Bergen	ECRHS III clin. respondents	346	National registers	499
Denmark, Aarhus	ECRHS III clin. respondents	68	National registers	83
Sweden, Uppsala	ECRHS III clin. respondents	74	National registers	90
Sweden, Göteborg	ECRHS III clin. respondents	53	National registers	60
Sweden, Umeå	ECRHS III clin. respondents	66	National registers	86
Iceland, Reykjavik	ECRHS III clin. respondents	97	National registers	120
Estonia, Tartu	ECRHS III clin. respondents	159	National registers	195
Australia, Melbourne	ECRHS III clin. respondents	102	Through the parents	144
Spain, Huelva	ECRHS III clin. respondents	38	Through the parents	62
Spain, Albacete	ECRHS III clin. respondents	38	Through the parents	66

For peer review only - http://bmjopen.bmj.com/site/about/guidelines.xhtml

*Parental information extracted from the ECRHS and harmonized with RHINE questions.

For peer review only

For peer review only - http://bmjopen.bmj.com/site/about/guidelines.xhtml

Table 2. Response rate for RHINESSA adult offspring (18+ years) participants for the questionnaire cohort and the clinical cohort. Eligible subjects were defined as live subjects 18 years and older with known residential addresses residing in the country (questionnaire cohort) or in/near the study centre (clinical cohort).

Center	*Questionnaire cohort*			*Clinical cohort*		
	Eligible*, N	Included, N	Response rate, %	Eligible*, N	Included, N	Response rate, %
Bergen	4385	1763	40.2	1278	499	39.0
Aarhus	4014	1224	30.5	381	83	21.8
Uppsala, Göteborg, Umeå	8256	3572	42.7	639	236	36.9
Reykjavik	4756	1245	26.2	200	120	60.0
Tartu	2907	618	21.3	669	195	29.1
Melbourne	499	245	49.1	245	144	58.8
Huelva	244	72	29.5	244	66	27.0
Albacete	365	79	21.6	365	62	17.0
Total	**25426**	**8818**	**34.7**	**4021**	**1405**	**34.9**

For peer review only - http://bmjopen.bmj.com/site/about/guidelines.xhtml

Parental cohorts

In the early 1990's the ECRHS conducted a population based survey among random samples of young adults aged 20-44 years in several European and non-European countries (www.ecrhs.org)[16]. On average 4000 persons (range 1000-7000) from each centre were invited to a postal survey (mean response rate 73%). Clinical examinations were conducted in subsamples from ~45 study centres. ECRHS followed up the clinical samples in 30 study centres in 2002-04 (ECRHS II)[17] and 2012-15 (ECRHS III)[18].

The RHINE study (www.rhine.nu) developed protocols to follow up responders to the initial ECRHS postal survey in seven Northern European centres: Bergen, Norway; Göteborg, Umeå and Uppsala, Sweden; Aarhus, Denmark; Reykjavik, Iceland and Tartu, Estonia. In 2000-2002 , 16106 persons answered extensive postal questionnaires (RHINE II, mean response rate 75%)[19, 20]. The population was reinvestigated in 2010-12, with 13093 answering a postal questionnaire. Analyses of non-response showed only minor differences between long-term participants and baseline participants in exposure-outcome associations between age, gender, smoking and respiratory symptoms[19].

Follow-up

Regular follow-up of the RHINESSA clinical and questionnaire cohorts is planned to take place with 5-8 years intervals. The first follow-up of the full cohort is about to start in all study centres in 2021-22. An *ad hoc* clinical follow-up was performed in Bergen study centre in 2020 to capture features related to the Covid-19 pandemic. The parent populations of all study centres have been followed with 8-10 year intervals since the 1990's, and the fourth study waves of RHINE and ECRHS are taking place in 2021-22.

Ethical consideration

For peer review only - http://bmjopen.bmj.com/site/about/guidelines.xhtml

Ethical permissions were obtained for each study wave from the local ethics committee in each of the participating centres. The ethical approval reference numbers are listed on www.rhinessa.net. All study participants provided written informed consent prior to participation. Permission to extract information about themselves and family members from national registers were obtained from each participant in the Northern European study centres. For children and adolescents participating in the additional study groups presented in the online supplementary, written informed consents were given by the parents/guardian, as required by the local ethics committees.

Response rate and parental characteristics related to offspring response

Identified offspring were sent an invitation letter with information about the study and log-in details to a web-based questionnaire, two reminders were sent, in some centres including a postal questionnaire. Persons eligible to a clinical study, were invited by a letter and/or contacted by telephone to agree on an appointment for clinical investigation, also with two reminders. For the three Swedish study centres, the researchers were not allowed to identify and approach study participants directly and Statistics Sweden distributed the invitation letters to participants of both the questionnaire and the clinical study. Altogether 8818 persons participated in the questionnaire cohort and 1405 of these in the clinical cohort (table 1). The overall response rate was 35% both for the questionnaire and the clinical cohort, with differences between study centres and between the questionnaire and clinical stages (table 2). Reasons for non-participation included inability to make contact with the persons due to erroneous contact details or because the person was no longer living at that address, as well as unwillingness or inability to participate. However, parental characteristics were compared between the responders and the source parental population (table 3), showing fairly similar characteristics and no clear patterns of differences, e.g. approximately 55% had a father or mother who had ever smoked in both groups, and there was e.g. no clear trend of higher response rates among offspring of symptomatic parents.

For peer review only - http://bmjopen.bmj.com/site/about/guidelines.xhtml

Table 3. Parental characteristics for RHINESSA adult (18+ years) responders compared to the source parental RHINE/ECRHS population for the questionnaire cohort and the clinical cohort.

	Questionnaire cohort		*Clinical cohort*	
	RHINE/ECRHS parents to RHINESSA adult offspring N = 6441	All RHINE N = 13260	RHINE/ECRHS parents to RHINESSA adult offspring N = 1041	All* ECRHS N =3205
**Paternal characteristics**				
Ever smoker, %	53.2	54.6	55.9	55.3
BMI (SD)	26.8 (4)	26.8 (4)	27.8 (4)	27.6 (4)
Overweight in puberty**, %	9.2	10.3	9.9	10.9
Asthma, %	12.3	10.3	23.7	17.6
Wheeze, %	20.1	20.7	28.6	27.1
**Maternal characteristics**				
Ever smoker, %	54.0	54.0	45.1	52.1
BMI (SD)	25.7 (5)	25.6 (5)	27.0 (5)	27.0 (5)
Overweight in puberty**, %	23.9	24.3	23.5	23.5
Asthma, %	14.3	13.6	23.1	26.4
Wheeze, %	20.2	19.3	24.4	27.7

* Only including data for the 10 study centres in RHINESSA.
** Overweight defined by self-reported body silhouette at age of menarche / age of voice break[29]

For peer review only - http://bmjopen.bmj.com/site/about/guidelines.xhtml

Collected data and characteristics of study participants

Data and biomaterial collected in RHINESSA include questionnaire/interview information on respiratory and general health, life style and environmental exposures; measurements of lung function, anthropometrics, blood pressure; allergy markers, sex hormones, DNA methylation, and biomarkers in urine and dust samples. Table 4 displays questionnaire/interview data, clinical measures, samples, and measured biomarkers that is available in the RHINESSA adult (18+ years) population, as well as information that is available from/about their parents, their grandparents and their offspring. In addition, national health registries in the Nordic countries with excellent coverage provide an additional data source for RHINESSA participants and family members of more generations.

For peer review only - http://bmjopen.bmj.com/site/about/guidelines.xhtml

Table 4. Key data available for the RHINESSA adult offspring (18+ years), and their grandparents, parents and offspring (*x available; ss available in subsample– see footnotes*).

	Grandparents<br>born 1898-1965	Parents<br>at 20-44 years<br>RHINE/ECRHS I	Parents<br>at 30-54 years<br>RHINE/ECRHS II	Parents<br>at 40-64 years<br>RHINE/ECRHS III	RHINESSA<br>adult offspring<br>18-53 years*	Offspring's<br>offspring<br>age 0-30 years
**Questionnaire/interview**						
Social class, education	x	x	x	x	x	ss
Childhood factors	x	x	x	x	x	ss
Birth characteristics**		ss			ss	ss
Place of upbringing	x	x	x	x	x	ss
Smoking	x	x	x	x	x	ss
Snus, oral moist tobacco, E-cigarettes					x	ss
Occupation	ss	x	x	x	x	ss
Indoor environment	ss	x	x	x	x	ss
Personal care products	ss			x	x	ss
Height/weight	ss	x	x	x	x	ss
Body shapes	x			x	x	ss
Waist circumference (self-measured)				x		
Physical activity	ss	ss	x	x	x	ss
Diet	ss	ss	ss	x	x	ss
Allergic diseases / symptoms	x	x	x	x	x	x
Asthma / symptoms	x	x	x	x	x	x
Asthma/ allergy treatment	ss	x	x	x	x	ss
Sleep	ss	ss	ss	x	x	ss
Other diseases/ symptoms	x	x	x	x	x	ss
Quality of life SF-36 /RAND	ss		ss	ss	ss	ss
Work disability			ss	x	x	
Air pollution and greenness***				ss	ss	
**Women questionnaire/interview** (from women in each cohort)						
Pregnancies and complications			x	x	x	ss
Birth characteristics of offspring			x	x	x	ss
Menarche, menstrual data, menopause			x	x	x	ss
Exogeneous sex hormones			x	x	x	ss
Irregular menstruation, PCOS			x	x	x	ss
Gynecological and related diseases			x	x	x	ss
**Clinical measures** (from clinical stage in each cohort)						
Anthropometry (height/weight/waist/hip)	ss	x	x	x	x	ss
Bioimpedance	ss			x	ss	ss

For peer review only

Spirometry (FEV1, FVC)	ss	x	x	x	x	ss
Post BD spirometry	ss			x	x	ss
Metacholine test		x	x			
FeNO	ss			x	x	ss
Skin prick test	ss	x		x	x	ss
Blood pressure	ss			x	x	ss
Heart rate	ss			x	x	ss
CIMT (carotis intima media)					ss	
CPI/caries index				ss	ss	
**Biological material and environmental samples** (from clinical stage in each cohort)						
Blood samples	ss	x	x	x	x	ss
Gingival samples	ss			ss	ss	ss
Skin swab	ss				ss	ss
Saliva	ss				ss	ss
Urine	ss	ss		x	x	ss
Bedroom dust samples	ss		ss	ss	ss	ss
**Biomarkers measured/funded at time of publication** (from clinical stage in each cohort)						
Total and specific IgEs	ss	x	x	x	x	ss
Genome wide genotyping			ss			
Selective genotyping			ss			
DNA methylation in fullblood	ss		ss	ss	ss	
Fasting blood glucose	ss			ss	ss	ss
Sex hormones			women	women	ss	ss
Oral microbiome	ss				ss	
Urine biomarkers of chemical exposures					ss	
Complete blood cell counts					ss	
Adipokines			ss			

* Sweden and Iceland did a shorter clinical protocol of RHINESSA adult offspring, not including bioimpedance, skin swap or saliva (except that Uppsala collected saliva). RHINESSA offspring <18 years were included in Aarhus, Bergen, Melbourne and Tartu, following age-adapted slightly shorter protocols, similar to protocols used for corresponding age groups in offspring's offspring.
** Information from registries and hospital protocols, and from mothers
*** Information generated using geo-coding based on registry data on life-time addresses

Subsamples:
Grandparents and offsprings' offspring were only investigated in Bergen, information in other centres are given by family members.
CIMT and CPI were only measured in Bergen.
Gingival samples were collected in parents and offspring from Bergen, Melbourne and Tartu, and in offspring from Uppsala.
DNA methylation was measured in fullblood using the Illumina EPIC BeadChip arrays in approximately 900 offspring, 400 parents and 140 grandparents.

For peer review only - http://bmjopen.bmj.com/site/about/guidelines.xhtml

Sex hormones were measured in mothers and approx. 1000 offspring from all centres.
Oral microbiome was measured using 16S rRNA Illumina MiSeq in Bergen adult/adolescent offspring and grandparent.
Urine biomarker concentrations of chemical exposure was measured in Bergen adult / adolescent offspring.
Complete blood cell counts were measured in Swedish centres, adipokines also in Reykjavik.
Helminth serology was measured in offspring from Bergen, Tartu and Aarhus, and parents from Bergen.

For peer review only

For peer review only - http://bmjopen.bmj.com/site/about/guidelines.xhtml

Tables 5a and 5b displays characteristics of the study population by study centre, separately presented for the questionnaire study population (5a) and the clinically investigated subsample (5b). Mean age at baseline was 30.1 years and there were 58% women, 33% had ever smoked and 21% had ever used oral moist tobacco (0.8% in Aarhus, 33.9% in Umeå). Asthma medication was used at the time of study by 8.7%, ranging from 6.5% in Aarhus to18.9% in Melbourne (table 5). The proportion of missing data ranges from <0.01 to 4.2% for key variables presented in tables 5a and b.

For peer review only - http://bmjopen.bmj.com/site/about/guidelines.xhtml

Table 5. Characteristics of the RHINESSA adult offspring (18+ years) cohorts by centre; 2a) questionnaire cohort (N=8818), and 2b) clinical cohort (N=1405).

a)

	Missing, %	Bergen	Aarhus	Uppsala	Göteborg	Umeå	Reykjavik	Tartu	Melbourne	Huelva	Albacete	*Total*
Age (mean, SD)	*0.4*	29.2 (7.4)	27.0 (7.4)	30.4 (7.6)	31.5 (8.0)	32.0 (7.5)	32.0 (8.1)	28.6 (6.2)	28.9 (6.5)	32.5 (7.0)	30.6 (7.1)	30.1 (7.7)
Sex, % females	*<0.1*	57.8	59.8	56.3	52.9	57.0	62.7	58.4	53.7	62.5	52.6	57.8
BMI (mean, SD)	*3.2*	24.3 (4.3)	23.7 (4.3)	24.0 (4.2)	24.5 (4.3)	24.6 (4.4)	26.2 (5.1)	23.8 (4.5)	23.7 (4.8)	24.2 (4.1)	23.9 (5.2)	24.4 (4.5)
Ever smoker, %	*1.9*	36.5	30.3	29.5	35.1	26.0	38.3	38.0	31.2	41.7	55.1	33.3
Ever used oral moist tobacco[a], %	*0.5*	29.6	4.1	23.1	24.3	33.9	15.7	9.2	0.8	N/A	N/A	20.5
Current smoker, %	*2.0*	12.7	15.2	8.8	14.4	7.6	14.1	21.5	13.9	33.3	32.1	13.0
Domestic ETS in childhood, %	*3.7*	54.8	50.8	37.0	49.3	43.5	61.2	55.8	24.6	54.2	63.6	49.4
Educational level:	*2.0*											
Primary school, %		2.6	2.1	2.5	2.4	2.2	5.3	7.2	0.0	1.4	6.6	3.1
Secondary educ. %		35.9	43.3	37.3	45.3	42.4	33.1	38.2	22.2	40.3	32.9	38.5
College, univ. %		61.5	54.6	60.2	52.3	55.4	61.6	54.6	77.8	58.3	60.5	58.4
Childhood asthma (onset <10 years), %	*1.1*	6.3	5.9	6.4	5.0	7.9	10.8	3.6	25.0	13.9	3.9	7.4
Current asthma medication, %	*<0.1*	7.6	6.5	9.9	8.1	11.4	8.6	3.9	18.9	9.7	14.3	8.7
Current hay fever / nose allergy, %	*0.4*	28.9	25.7	29.6	27.9	26.9	32.2	27.3	47.3	36.1	35.1	29.1
Childhood atopic dermatitis (onset <10 years), %	*4.0*	6.7	8.5	8.9	9.7	8.0	10.3	5.3	9.8	0.0	3.9	8.2
Current atopic dermatitis, %	*<0.1*	8.6	8.2	13.0	11.3	10.0	14.5	11.0	11.1	5.6	12.8	10.8

[a] Not available information on e-cigarettes in centres labelled N/A

[b] Defined as ever having had itchy rash that was coming and going for at least 6 months, and that the rash affected any of the following places: the fold of the elbows, behind the knees, in front of the ankles, under the buttocks or around the neck, ears or eyes.

For peer review only - http://bmjopen.bmj.com/site/about/guidelines.xhtml

b)

	Missing %	Bergen	Aarhus	Uppsala	Göteborg	Umeå	Reykjavik	Tartu	Melbourn3	Huelva	Albacete	*Total*
Age (SD)	*0.2*	28.0 (6.6)	28.2 (8.2)	31.4 (7.8)	31.3 (7.5)	31.2 (7.3)	34.6 (8.1)	29.8 (5.8)	29.1 (6.5)	32.2 (7.1)	31.0 (7.7)	29.9 (7.2)
Sex, % females	*0.2*	47.3	62.2	68.9	55.2	55.3	56.8	44.3	53.2	63.6	47.5	52.1
BMI (SD)	*0.3*	25.1 (4.5)	24.4 (5.0)	25.3 (5.5)	24.6 (3.5)	25.2 (4.4)	28.1 (5.1)	24.9 (5.0)	25.0 (4.5)	24.3 (4.5)	24.2 (5.0)	25.2 (4.8)
Ever smoker, %	*0.6*	29.9	25.3	30.0	30.0	17.4	45.0	38.5	26.4	43.9	51.6	32.6
Current smoker, %	*0.2*	13.9	16.9	4.5	5.0	1.2	9.2	19.5	6.9	28.8	29.0	13.4
Domestic ETS in childhood[a], %	*0.8*	54.2	44.6	N/A	N/A	N/A	N/A	53.9	36.8	53.0	75.8	52.2
Childhood asthma, onset <10 years, %	*1.1*	4.4	8.4	6.7	3.5	5.8	10.9	2.1	19.4	9.2	3.2	6.8
Current asthma medication, %	*3.1*	4.6	3.6	8.9	0.0	5.8	2.5	1.5	18.1	6.2	6.5	5.6
Current hay fever/ nose allergy, %	*3.7*	31.5	31.3	38.2	50.0	37.7	29.2	26.7	51.4	31.8	29.0	34.1
Childhood atopic dermatitis, onset <10 years) [b], %	*0.9*	5.4	2.4	5.6	3.3	4.7	9.2	5.1	20.1	3.0	1.6	6.6
Current atopic dermatitis, %	*0.1*	7.2	6.0	10.1	10.0	5.8	14.3	3.6	16.0	6.1	3.2	8.1
FEV_1 l (SD)	*1.6*	3.91 (0.8)	3.80 (0.7)	3.56 (0.7)	3.77 (0.7)	3.72 (0.7)	3.64 (0.8)	4.08 (0.8)	3.78 (0.8)	3.51 (0.7)	3.63 (0.6)	3.83 (0.8)
FVC l (SD)	*1.9*	4.80 (1.1)	4.65 (0.9)	4.42 (0.9)	4.70 (0.9)	4.73 (0.9)	4.60 (1.0)	4.97 (1.0)	4.69 (0.9)	4.29 (0.9)	4.28 (0.8)	4.73 (1.0)
FEV_1/FVC (SD)	*2.0*	0.82 (0.1)	0.82 (0.1)	0.81 (0.1)	0.80 (0.1)	0.79 (0.1)	0.79 (0.1)	0.82 (0.1)	0.81 (0.1)	0.82 (0.1)	0.85 (0.1)	0.81 (0.1)
FEV_1 % pred[c]. (SD)	*4.2*	95 (11)	94 (10)	94 (13)	93 (11)	93 (10)	94 (12)	97 (11)	95 (13)	96 (11)	95 (11)	95 (11)
FVC % pred[c]. (SD)	*4.2*	98 (11)	97 (10)	97 (12)	96 (10)	98 (10)	98 (11)	98 (10)	99 (11)	97 (10)	93 (12)	98 (11)
FEV_1/FVC% pred (SD)	*4.2*	97 (7)	96 (6)	96 (7)	96 (6)	94 (6)	96 (6)	98 (7)	95 (7)	98 (7)	102 (7)	97 (7)

[a] Not available information on ETS exposure in centres labelled N/A

[b] Defined as ever having had itchy rash that was coming and going for at least 6 months, and that the rash affected any of the following places: the fold of the elbows, behind the knees, in front of the ankles, under the buttocks or around the neck, ears or eyes.

[c] Calculated based on Global Lung function Initiative GLI2012 reference values (Quanjer et al, ERJ 2012)

Definitions of asthma, hay fever/ nose allergies and atopic dermatitis are the same as in the questionnaire study presented above in Table 2a, but based on information obtained by standardised interviews rather than self-filled in questionnaires.

The presented lung function data refer to pre-bronchodilator measurements.

For peer review only

For peer review only - http://bmjopen.bmj.com/site/about/guidelines.xhtml

Participant and public involvement

User representatives from the Norwegian Asthma and Allergy Foundation and from the Norwegian Labour Inspection have been involved in the RHINESSA Advisory Board from the first meetings when initial plans for the study were discussed, and have contributed to development of the study as well as priorities in analyses and publication of data. For instance, the Norwegian Labour Inspection in particular promoted data collection and research to shed light on risks related to occupational cleaning. The general public and the participants have not otherwise been involved in the design or conduct of the research.

One study participant has at a later stage been included in the RHINESSA Advisory Board Study, as user representative of the study population, and contributes to discussions regarding all aspects of the research, in particular research priorities and dissemination of study results. From the beginning of the study, information on the research has been available to the study participants through the study website and newsletters. Field workers have been alert to comments from the study participants and conveyed these experiences in annual meetings, but the study participants have not formally been asked to assess the burden related to participating in the research.

FINDINGS TO DATE

Smoking and overweight in male prepuberty and offspring health

A first explorative analysis of asthma in >24.000 offspring of the RHINE cohort[21], suggested that father's smoking before conception was associated with asthma in future offspring. Father's smoking debut before age 15 and more than 10 years of preconception smoking were associated with offspring asthma, while time of quitting or numbers of cigarettes smoked daily were not. Mother's smoking around the time of pregnancy, but not before conception, was associated with offspring asthma. Asthma was further associated with paternal grandmother's smoking. A multi-generation analysis of the ECRHS cohort[22] by Accordini et al. confirmed that father's pre-pubertal smoking was

For peer review only - http://bmjopen.bmj.com/site/about/guidelines.xhtml

associated with higher asthma risk in offspring. Statistical mediation models were used to account for the complexity in the multi-centre multi-generation data, including simulation analyses showing that the impact of unmeasured confounding on the estimates was limited[23, 24]. State-of-the-art statistical methods for causal inference from observational data[25] were applied in a subsequent analysis of the RHINESSA/ECRHS cohorts, suggesting that father's smoking <15 years caused lower lung function in offspring[26]. Impact on both FVC and FEV_1 suggested detrimental effects on lung growth/size as well as airway obstruction. Effects of father's smoking across generations are supported by preliminary mechanistic work. Involvement of epigenetic mechanisms was suggested by Knudsen TM et al. based on a preliminary EWAS analyses, showing that father's smoking was associated with specific DNA methylation patterns in adult offspring[27]. A murine study of cigarette smoke exposure from puberty onset in male mice, found that preconception smoke exposure altered miRNAs in the spermatozoa, and gave higher postnatal body weight in progeny[28].

Further support for early male puberty as an important susceptibility window was obtained in a study of parents' overweight starting by childhood, puberty, or young adulthood. Johannessen et al. found that father's onset of overweight between age 8 years and voice break was associated with asthma in future offspring[29]. This effect was not mediated by the offspring's own overweight, and there were no significant effects of father's overweight starting after puberty or mother's overweight before conception. An ongoing analysis by Lønnebotn et al. suggests that father's prepubertal overweight also may cause lower lung function in offspring[30]. Investigating ***overweight as outcome***, Knudsen et al. demonstrated that father's prepubertal smoking onset was associated with excessive fat mass in their future sons[31]. Johannessen et al. showed that father's and mother's overweight in childhood, and mother's overweight at menarche, were associated with offspring overweight in childhood[29].

Other preconception exposures in mothers and fathers and offspring health

For peer review only - http://bmjopen.bmj.com/site/about/guidelines.xhtml

Bertelsen et al. found that ***parental asthmatic and allergic disease activity*** *before* conception was more strongly associated with offspring allergic asthma, than parental disease activity *after* the child was born[32]. Shared environment would have given stronger associations with parental postnatal disease activity, and genetic inheritance would have given similar associations for preconception and postnatal disease activity. The identified pattern might possibly reflect an influence of asthmatic/allergic disease activity on germline cells and thereby on future offspring phenotype. A study of ***parental immune response to helminths*** in Norway by Jõgi et al. uncovered that IgG4 to the zoonotic helminth *Toxocara* was associated with more asthma symptoms and hay fever in a younger offspring generation but not in an older parent generation. Further, parental *Toxocara* IgG4 was associated with allergic symptoms in their offspring. The timing of parental exposure in relation to offspring birth year could not be determined, but the associations followed a sex-specific pattern[33]. Preliminary analyses from López-Cervantes found that parental tuberculosis with onset in childhood was associated with offspring asthma[34]. Timm et al. explored whether ***farm upbringing in previous generations*** could influence offspring asthma and allergies, and found no evidence of an association between parental/grandparental farm upbringing and offspring asthma[35]. Regarding selective migration which has not previously been studied across three generations, an analysis suggested that asthma in the family was not a risk factor for quitting farming[36]. Regarding ***parental occupational exposures***, Svanes et al. found that father's welding ≥10 years before conception was associated with a doubled risk of asthma in future offspring[21]. Pape et al. investigated four groups of exposures defined from an asthma-specific job exposure matrix (JEM), and compared exposure only before conception with exposure starting before conception and continuing. Associations with offspring asthma were not identified for most exposure groups, an exception was higher risk of early-onset asthma if the mother had been occupationally exposed to "allergens and reactive chemicals" both before and after the offspring's birth[37]. Tjalvin et al. investigated the specific exposure category "indoor cleaning agents: cleaning products/detergents and disinfectants", an exposure present in 21

For peer review only - http://bmjopen.bmj.com/site/about/guidelines.xhtml

ISCO-88 job codes such as nurses, personal care workers, cooks and cleaners[38]. The analysis found that exposure starting before conception was associated with higher asthma risk in offspring, while there was no association with exposure starting after birth. Kuiper et al.[39] analysed ***parental air pollution** in* childhood/adolescence as related to offspring asthma and hayfever. Data on various air pollutants in parents from 1975 onwards were generated by geocoding of parental individual residential addresses obtained from national registries. The analysis found that mother's PM10 exposure before age 18 years had a direct effect with doubled asthma risk in offspring, and that father's ozone exposure in the same age window was associated with increased offspring hayfever risk[39].

Heritability in symptoms and diseases across generations

In a study of ***sleep disturbances***, Lindberg et al. showed that sleep-related symptoms and sleep duration were more common in offspring whose parents had reported the same symptom, consistent after adjusting for lifestyle factors, education and parity[40]. Ekström et al. found that ***breathlessness*** was nearly doubled in offspring of parents with breathlessness, even when adjusting for factors associated with breathlessness in both generations (obesity, smoking, depression, asthma, lower lung function and female sex)[41]. Carsin et al. found that ***grandfather's cardiometabolic disease*** (CMD) was directly associated with grandoffspring asthma, while accounting for indirect effects transmitted through parental CMD or asthma, consistently in the RHINE, ECRHS cohorts and RHINESSA cohorts (not yet published).

Validation studies informing multi-generation epidemiological research

Information about family members is often sought from study participants, as this is cost-effective and may be the only feasible way to obtain the information. The RHINESSA/RHINE/ECRHS cohorts provide an important opportunity for validation of such information. Kuiper et al. found a

For peer review only - http://bmjopen.bmj.com/site/about/guidelines.xhtml

moderate to good agreement between self-reported asthma and asthma reported by family members, both regarding offspring asthma reported by parents, and parents' asthma reported by offspring[42]. The reporting was better for childhood onset *versus* later onset asthma. Pape et al. found that adults reported quite accurately their parents' smoking during their childhood and their mother' smoking when pregnant with them, when compared with the parents' own report[43]. The reporting was better for higher *versus* lower intensity smoking, and for mother's *versus* father's smoking. Timm et al. found that the accuracy in reporting parental place of upbringing was dependent on own place of upbringing, but this did not bias the associations of place of upbringing with asthma[44]. Skulstad et al. validated mothers' information about births and pregnancies against data from the Medical Birth Registry of Norway. The analysis found high validity for mother's report of important birth and pregnancy parameters, and that risk-associations were similar when using maternal *versus* registry based information[45].

Life course data on obesity is rarely available for multiple generations. The RHINESSA/RHINE/ECRHS studies have included a tool with pictorial drawings of body silhouettes in childhood, voice break/menarche and adult ages. Dratva et al. found that current body silhouettes were highly correlated with BMI calculated from measured or self-reported weight and height[46]. Lønnebotn et al. found that retrospective body silhouettes from adult ages correlated well with BMI calculated from measured height and weight at the corresponding ages in the past, and allowed for differentiation of obesity and non-obesity[47].

STRENGTHS AND LIMITATIONS

The main strength of the RHINESSA study is the large number of offspring-parent pairs with rich and high-quality information from both generations. Both fathers and mothers have been extensively characterised over twenty years of childbearing age, and the availability of such parental exposure

For peer review only - http://bmjopen.bmj.com/site/about/guidelines.xhtml

information for adolescent and adult offspring is quite unique. The prospectively and retrospectively collected data on family members in this multi-generation study have allowed validation of information provided about family members, thereby extending the number of generations that can be analysed in a robust manner. The multi-centre structure is a strength in terms of larger external validity; while the largest number of study participants are from the relatively homogeneous Nordic countries, the Estonian, Spanish and Australian study centres contribute to the diversity in the study population improving the external generalisability. The excellent population- and health registries in the Nordic countries represent a major strength of the study, family members can be identified in an unbiased manner and a wealth of data are available for all generations. For some study centres there is information on five generations, covering birth cohorts born over more than one century – the century when the welfare societies were established in many Western societies.

A weakness of the RHINESSA study is that detailed parental data are mostly available for one parent of the offspring, the parent (mother or the father) participating in the ECRHS and RHINE studies. To meet this challenge, a sub-cohort of the "other" parents has been studied in Bergen RHINESSA, validation studies have been performed to improve the usefulness of information reported by offspring on both parents, and there are registry data available for both parents in North European study centres. Another weakness is the relatively low response rates. Fortunately, exposure information in terms of parental information is available for responders and non-responders. While selection bias cannot be ruled out, it is reassuring that table 4 suggests similar parental characteristics for responding and non-responding offspring. In study centres with the appropriate parental consent, information on a number of health outcomes in offspring can be obtained from national registries. Finally, the multi-generation multi-centre study design is challenging with regard to standardization of data collection over time and between generations and study centres, and random heterogeneity in the data due to this may attenuate true results.

For peer review only - http://bmjopen.bmj.com/site/about/guidelines.xhtml

So, what lessons have we learned from the cohort's creation that could be useful for other researchers? One most useful contribution from RHINESSA to other researchers, is the possibility to validate information provided by family members. In general, we find that strong, longstanding collaboration and friendship has been key for creating a complex set of cohorts in a longitudinal multi-centre setting. Thus, building on existing cohorts with well-functioning researcher networks appears to be important for future multi-generation epidemiological studies.

For peer review only

For peer review only - http://bmjopen.bmj.com/site/about/guidelines.xhtml

COLLABORATION

Requests for collaboration with RHINESSA and access to data can be made to the RHINESSA steering committee by PI Professor Cecilie Svanes (cecilie.svanes@uib.no) or vice PI Professor Vivi Schlünssen (vs@ph.au.dk). Reuse of the data must be done in collaboration with the RHINESSA study team, and we in particular encourage collaboration related to multi-generation analyses. Further information including issues on data security and sharing of data and additional study results can be found at www.rhinessa.net.

FUNDING

Co-ordination of the RHINESSA study has received funding from the Research Council of Norway (Grants No. 274767, 214123, 228174, 230827 and 273838), ERC StG project BRuSH #804199, the European Union's Horizon 2020 research and innovation programme under grant agreement No. 633212 (the ALEC Study WP2), the Bergen Medical Research Foundation, and the Western Norwegian Regional Health Authorities (Grants No. 912011, 911892 and 911631). Study centres have further received local funding from the following: **Bergen**: the above grants for study establishment and co-ordination, and, in addition, World University Network (REF and Sustainability grants), Norwegian Labour Inspection, the Norwegian Asthma and Allergy Association and Trond Mohn Foundation (Grant ID BFS2017TMT02). **Albacete** and **Huelva**: Sociedad Española de Patología Respiratoria (SEPAR) Fondo de Investigación Sanitaria (FIS PS09). **Gøteborg**, **Umeå** and **Uppsala**: the Swedish Heart and Lung Foundation, the Swedish Asthma and Allergy Association. **Reykjavik**: Iceland University. **Melbourne**: Australian National Health and Medical Research Council (NHMRC) Project Grant ID1128450. **Tartu**: the Estonian Research Council (Grant No. PUT562). **Århus**: The Danish Wood Foundation (Grant No. 444508795), the Danish Working Environment Authority (Grant No. 20150067134), Aarhus University (PhD scholarship).

For peer review only - http://bmjopen.bmj.com/site/about/guidelines.xhtml

The **RHINE** study received funding by Norwegian Research Council, Norwegian Asthma and Allergy Association, Danish Lung Association, Swedish Heart and Lung Foundation, Vårdal Foundation for Health Care Science and Allergy Research, Swedish Asthma and Allergy Association, Icelandic Research Council, and Estonian Science Foundation. The RHINE IV ongoing study has received funding from the Research Council of Norway project Life-GAP grant No. 300765.

Co-ordination of the ECRHS study has received funding from the European Union's Horizon 2020 research and innovation programme under grant agreement No. 633212 (the ALEC study), the Medical Research Council (ECRHS III) and the European Commission FP5 and FP7 (ECRHS I and II). The ECRHS IV ongoing study in the ten RHINESSA study centres has received funding from the European Union's Horizon 2020 research and innovation programme projects EPHOR under grant agreement No. 874703 and European Research Council (ERC) project BRuSH under grant agreement No. 804199, and from the Research Council of Norway grant No. 273838. Funding agencies for ECRHS I, II and III are reported in the online supplement.

The funding agencies have had no direct role in the conduct of the study or the data collection and management, nor of data analysis or manuscript preparation.

ACKNOWLEDGEMENTS

We thank all RHINESSA and RHINE/ECRHS study participants and fieldworkers. A special thank to the fieldworkers at the Research Unit for Health Surveys, University of Bergen. We also thank the following: The RHINESSA study is led by Cecilie Svanes (PI) and Vivi Schlünssen (vice PI), and a **steering committee** that includes study centre PIs Francisco Javier Callejas (Albacete), Randi

For peer review only

J Bertelsen (Bergen), Mathias Holm (Göteborg), Jose Luis Sanchez (Huelva), Bryndis Benediktsdottir (Reykjavik), Shyamali Dharmage (Melbourne), Nils Oskar Jõgi (Tartu), Anna Oudin (Umeå), Andrei Malinovschi (Uppsala) and Vivi Schlünssen (Århus), and researchers with specific functions: Christer Janson (PI RHINE), Debbie Jarvis (PI ECRHS), Joachim Heinrich (SC ECRHS), Ane Johannessen (lead data management), Randi Bertelsen (lead biobank), Francisco Gómez Real (lead women's studies), Julia Dratva (children's and adolescent health), Thorarinn Gislason (sleep), Bertil Forsberg (air pollution), Dan Norbäck (indoor environment), Torben Sigsgaard (biodiversity, farm environment), Kjell Torén (occupation and disability). The **co-ordinating centre** in Bergen includes researchers Cecilie Svanes (lead RHINESSA), Randi J Bertelsen (lead biobank), Ane Johannessen (lead data management), Francisco Gomez Real (lead women's studies), and a support team with administrative leader/project coordinator Vilde Marie Svanes Sørbye (2015-d.d) and Eirunn Waatevik Saure (2012-15), assistant project coordinator Benedikte Svanes Sørbye, data managers Bente Sved Skottvoll and Eivind Rebnord, and lead field work Nina Særvold. The RHINESSA Scientific Advisory Board includes Simone Accordini (transgenerational statistical analyses), Jan Vilhelm Bakke (the Norwegian Labour inspection), Hogne Skogesal (the Norwegian Asthma and Allergy Association), Karin Lødrup Carlsen (paediatric asthma, children cohorts), Susanne Krauss-Etschmann (transgenerational animal models), Joachim Heinrich (respiratory epidemiology, children cohorts), John Holloway (epigenomics), William Horsnell (immunology, animal models), Deborah Jarvis (PI ECRHS, respiratory epidemiology), Francine Kaufmann (respiratory epidemiology, genetic epidemiology), Torvid Kiserud (foetal medicine), Stephanie London (genetic epidemiology), Benjamin Marsland (airways microbiome), Juha Pekkanen (respiratory epidemiology) and Jordi Sunyer (epidemiology, children cohorts). The related **ECRHS** study is led by Deborah Jarvis and Peter Burney (ECRHS co-ordinating team in London), and the **RHINE** study by Christer Janson (RHINE co-ordinator, Uppsala). Standardisation of procedures in ECRHS has guided standardisation of procedures in the RHINESSA clinical study phases, and

For peer review only

standardisation of ECRHS and RHINE questionnaires has guided the RHINESSA questionnaire study phases.

RHINESSA investigators

Albacete: Francisco Javier Callejas (PI), Raúl Godoy (vice PI), Jesus Martínez-Moratalla (former PI). **Bergen**: Randi J Bertelsen (PI), Trude Duelien Skorge (vice PI), Christine Drengenes, Ane Johannessen, Nils Oskar Jõgi, Maryia Khomich, Jorunn Kirkeleit, Toril Mørkve Knudsen, Ingrid Kuiper, Juan Pablo López-Cervantes, Marianne Lønnebotn, Shokouh Makvandi-Nejad, Antonio Pérez, Francisco Gomez Real, Anders Røsland, Rajesh Shigdel, Svein Magne Skulstad, Torgeir Storaas, Cecilie Svanes, Øistein Svanes, Kai Triebner, Gro Tjalvin, Hilde Vindenes, Shanshan Xu. **Göteborg**: Mathias Holm (PI), Kjell Torén. **Huelva**: Jose Luis Sanchez (PI), Jose Maldonado (vice PI). **Reykjavik**: Bryndis Benediktsdottir (PI), Thorarinn Gislason (vice PI). **Melbourne**: Shyamali Dharmage (PI), Caroline Lodge (vice PI), Michael Abramson, Lyle Gurrin, Adrian Lowe, David Martino, Gita Mishra, Jennifer Perret, Bruce Thompson. **Tartu**: Nils Oskar Jõgi (PI), Rain Jõgi (vice PI). **Umeå**: Anna Oudin (PI), Lennart Bräbeck (former PI), Bertil Forsberg (vice PI), Karl Franklin. **Uppsala**: Andrei Malinovschi (PI), Christer Janson (vice PI), Dan Norbäck, Eva Lindberg, Magnus Ekstrøm. **Århus**: Vivi Schlünssen (PI), Christine Cramer, Kathrine Pape, Torben Sigsgaard, Signe Timm, Anne Mette Lund Würtz. **Other**: Simone Accordini, Lucia Calciano, Julia Dratva, Joachim Heinrich, John Holloway, William Horsnell, Deborah Jarvis, Susanne Krauss-Etchmann.

For peer review only - http://bmjopen.bmj.com/site/about/guidelines.xhtml

Legend to figures

Figure 1. The RHINESSA multi-generation study provides data and biomaterial to study how factors in girls and boys, during different age windows, can influence the health of their future children. Factors such as smoking, overweight and air pollution could influence the developing and maturing germ cells in both sexes, and a "fingerprint" of such exposures could be transferred to future offspring and thereby influence their phenotype.

Figure 2. The RHINESSA adult offspring cohort (generation 3 "**G3**") includes 8818 young men and women investigated with questionnaires (q), of which 1405 were investigated clinically (c). These are the offspring of men and women participating in the RHINE/ECRHS studies (**G2**) who were followed up over twenty years. In addition, Aarhus, Bergen, Melbourne and Tartu study centres investigated **G3** offspring age 4-17 years (1139q/ 201c), and Bergen study centre investigated **G1** (1470q/145c), the **other G2** parent (910q/152c) and **G4** (750q/433c). In all study centres G3 and G2 study participants provided information about their parents and offspring in G1 and G4.

For peer review only

For peer review only - http://bmjopen.bmj.com/site/about/guidelines.xhtml

References

1. Pembrey M, Saffery R, Bygren LO. Human transgenerational responses to early-life experience: potential impact on development, health and biomedical research. *J Med Genet* 2014; **51**: 563-72.
2. Soubry A, Hoyo C, Jirtle RL, Murphy SK. A paternal environmental legacy: evidence for epigenetic inheritance through the male germ line. *Bioessays* 2014; **36**: 359-71.
3. Morkve Knudsen T, Rezwan FI, Jiang Y, Karmaus W, Svanes C, Holloway JW. Transgenerational and intergenerational epigenetic inheritance in allergic diseases. *J Allergy Clin Immunol* 2018; **142**: 765-72.
4. Jawaid A, Jehle KL, Mansuy IM. Impact of Parental Exposure on Offspring Health in Humans. *Trends Genet* 2021; **37**: 373-88.
5. Golding J, Pembrey M, Iles-Caven Y, Watkins S, Suderman M, Northstone K. Ancestral smoking and developmental outcomes: a review of publications from a population birth cohort†. *Biol Reprod* 2021; **105**: 625-31.
6. Svanes C, Bertelsen RJ, Accordini S, et al. Exposures during the prepuberty period and future offspring's health: evidence from human cohort studies†. *Biol Reprod* 2021; **105**: 667-80.
7. Krauss-Etschmann S, Meyer KF, Dehmel S, Hylkema MN. Inter- and transgenerational epigenetic inheritance: evidence in asthma and COPD? *Clin Epigenetics* 2015; **7**: 53.
8. Nilsson EE, Sadler-Riggleman I, Skinner MK. Environmentally induced epigenetic transgenerational inheritance of disease. *Environ Epigenet* 2018; **4**: dvy016.
9. Heard E, Martienssen RA. Transgenerational epigenetic inheritance: myths and mechanisms. *Cell* 2014; **157**: 95-109.
10. Hammer B, Wagner C, Divac Rankov A, et al. In utero exposure to cigarette smoke and effects across generations: A conference of animals on asthma. *Clin Exp Allergy* 2018; **48**: 1378-90.
11. Pembrey ME, Bygren LO, Kaati G, et al. Sex-specific, male-line transgenerational responses in humans. *European journal of human genetics : EJHG* 2006; **14**: 159-66.
12. Bygren LO, Kaati G, Edvinsson S. Longevity determined by paternal ancestors' nutrition during their slow growth period. *Acta Biotheor* 2001; **49**: 53-9.
13. Li YF, Langholz B, Salam MT, Gilliland FD. Maternal and grandmaternal smoking patterns are associated with early childhood asthma. *Chest* 2005; **127**: 1232-41.
14. Bline AP, Dearfield KL, DeMarini DM, Marchetti F, Yauk CL, Escher J. Heritable hazards of smoking: Applying the "clean sheet" framework to further science and policy. *Environ Mol Mutagen* 2020; **61**: 910-21.
15. Behrman JR, Calderon MC, Preston SH, Hoddinott J, Martorell R, Stein AD. Nutritional supplementation in girls influences the growth of their children: prospective study in Guatemala. *Am J Clin Nutr* 2009; **90**: 1372-9.
16. Burney PG, Luczynska C, Chinn S, Jarvis D. The European Community Respiratory Health Survey. *The European respiratory journal* 1994; **7**: 954-60.
17. Chinn S, Jarvis D, Burney P, et al. Increase in diagnosed asthma but not in symptoms in the European Community Respiratory Health Survey. *Thorax* 2004; **59**: 646-51.
18. Jarvis D, Newson R, Janson C, et al. Prevalence of asthma-like symptoms with ageing. *Thorax* 2018; **73**: 37-48.
19. Johannessen A, Verlato G, Benediktsdottir B, et al. Longterm follow-up in European respiratory health studies - patterns and implications. *BMC Pulm Med* 2014; **14**: 63.
20. Janson C, Johannessen A, Franklin K, et al. Change in the prevalence asthma, rhinitis and respiratory symptom over a 20 year period: associations to year of birth, life style and sleep related symptoms. *BMC Pulm Med* 2018; **18**: 152.

For peer review only - http://bmjopen.bmj.com/site/about/guidelines.xhtml

21. Svanes C, Koplin J, Skulstad SM, et al. Father's environment before conception and asthma risk in his children: a multi-generation analysis of the Respiratory Health In Northern Europe study. *International journal of epidemiology* 2017; **46**: 235-45.
22. Accordini S, Calciano L, Johannessen A, et al. A three-generation study on the association of tobacco smoking with asthma. *International journal of epidemiology* 2018; **47**: 1106-17.
23. Arah OA. Commentary: Tobacco smoking and asthma: multigenerational effects, epigenetics and multilevel causal mediation analysis. *International journal of epidemiology* 2018; **47**: 1117-9.
24. Lutz SM, Thwing A, Schmiege S, et al. Examining the role of unmeasured confounding in mediation analysis with genetic and genomic applications. *BMC Bioinformatics* 2017; **18**: 344.
25. Lederer DJ, Bell SC, Branson RD, et al. Control of Confounding and Reporting of Results in Causal Inference Studies. Guidance for Authors from Editors of Respiratory, Sleep, and Critical Care Journals. *Ann Am Thorac Soc* 2019; **16**: 22-8.
26. Accordini S, Calciano L, Johannessen A, et al. Prenatal and prepubertal exposures to tobacco smoke in men may cause lower lung function in future offspring: a three-generation study using a causal modelling approach. *The European respiratory journal* 2021; **58**.
27. Mørkve Knudsen GT, Rezwan FI, Johannessen A, et al. Epigenome-wide association of father's smoking with offspring DNA methylation: a hypothesis-generating study. *Environ Epigenet* 2019; **5**: dvz023.
28. Hammer B, Kadalayil L, Boateng E, et al. Preconceptional smoking alters spermatozoal miRNAs of murine fathers and affects offspring's body weight. *Int J Obes (Lond)* 2021; **45**: 1623-7.
29. Johannessen A, Lonnebotn M, Calciano L, et al. Being overweight in childhood, puberty, or early adulthood: Changing asthma risk in the next generation? *J Allergy Clin Immunol* 2020; **145**: 791-9 e4.
30. Lønnebotn M, Calciano C, Accordini S, et al. Lung function in adult offspring as associated with their father's overweight in childhood/puberty. *European Respiratory Journal* 2020; **56**: 2066.
31. Knudsen GTM, Dharmage S, Janson C, et al. Parents' smoking onset before conception as related to body mass index and fat mass in adult offspring: Findings from the RHINESSA generation study. *PloS one* 2020; **15**: e0235632.
32. Bertelsen RJ, Rava M, Carsin AE, et al. Clinical markers of asthma and IgE assessed in parents before conception predict asthma and hayfever in the offspring. *Clin Exp Allergy* 2017; **47**: 627-38.
33. Jogi NO, Svanes C, Siiak SP, et al. Zoonotic helminth exposure and risk of allergic diseases: A study of two generations in Norway. *Clin Exp Allergy* 2018; **48**: 66-77.
34. López-Cervantes JP SR, Mustafa T, Accordini S, Svanes C. . Does parental tuberculosis infection increase the risk of asthma in their offspring? A Norwegian registry-based study. . *Tropical Medicine & International Health* 2021; **26**: 251.
35. Timm S, Svanes C, Frydenberg M, et al. Does parental farm upbringing influence the risk of asthma in offspring? A three-generation study. *International journal of epidemiology* 2020.
36. Timm S, Frydenberg M, Abramson MJ, et al. Asthma and selective migration from farming environments in a three-generation cohort study. *Eur J Epidemiol* 2019; **34**: 601-9.
37. Pape K, Svanes C, Sejbaek CS, et al. Parental occupational exposure pre- and post-conception and development of asthma in offspring. *International journal of epidemiology* 2020.
38. Tjalvin G, Svanes Ø, Igland J, et al. Maternal preconception occupational exposure to cleaning products and disinfectants and offspring asthma. *J Allergy Clin Immunol* 2021.

For peer review only - http://bmjopen.bmj.com/site/about/guidelines.xhtml

39. Kuiper IN, Markevych I, Accordini S, et al. Associations of Preconception Exposure to Air Pollution and Greenness with Offspring Asthma and Hay Fever. *Int J Environ Res Public Health* 2020; **17**.
40. Lindberg E, Janson C, Johannessen A, et al. Sleep time and sleep-related symptoms across two generations - results of the community-based RHINE and RHINESSA studies. *Sleep Med* 2020; **69**: 8-13.
41. Ekström M, Johannessen A, Abramson MJ, et al. Breathlessness across generations: results from the RHINESSA generation study. *Thorax* 2021.
42. Kuiper IN, Svanes C, Benediktsdottir B, et al. Agreement in reporting of asthma by parents or offspring - the RHINESSA generation study. *BMC Pulm Med* 2018; **18**: 122.
43. Pape K, Svanes C, Malinovschi A, et al. Agreement of offspring-reported parental smoking status: the RHINESSA generation study. *BMC Public Health* 2019; **19**: 94.
44. Timm S, Schlunssen V, Benediktsdottir B, et al. Offspring Reports on Parental Place of Upbringing: Is It Valid? *Epidemiology* 2019; **30**: e16-e8.
45. Skulstad SM, Igland J, Johannessen A, et al. Validation of maternal reported pregnancy and birth characteristics against the Medical Birth Registry of Norway. *PloS one* 2017; **12**: e0181794.
46. Dratva J, Bertelsen R, Janson C, et al. Validation of self-reported figural drawing scales against anthropometric measurements in adults. *Public Health Nutr* 2016; **19**: 1944-51.
47. Lonnebotn M, Svanes C, Igland J, et al. Body silhouettes as a tool to reflect obesity in the past. *PloS one* 2018; **13**: e0195697.

For peer review only

For peer review only - http://bmjopen.bmj.com/site/about/guidelines.xhtml

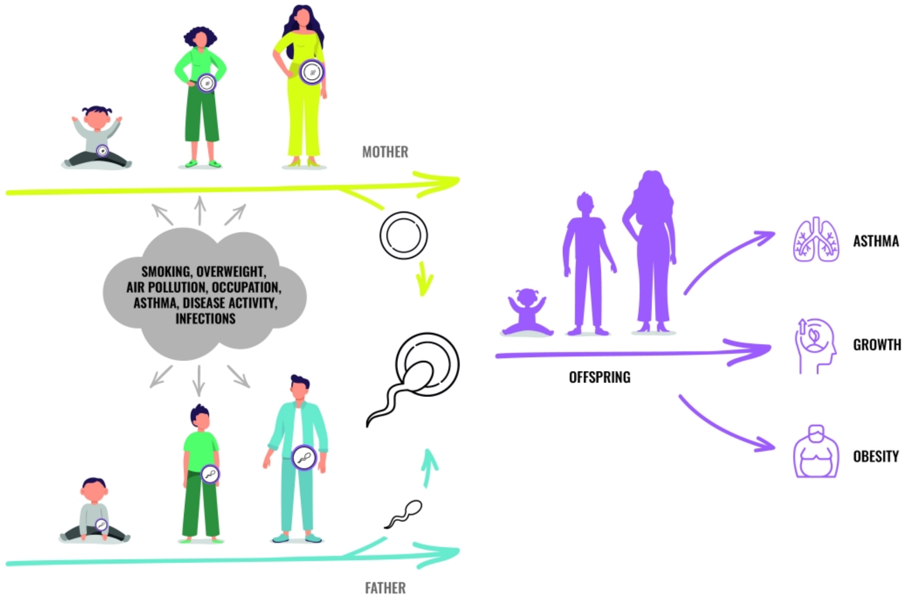

Figure 1. The RHINESSA multi-generation study provides data and biomaterial to study how factors in girls and boys, during different age windows, can influence the health of their future children. Factors such as smoking, overweight and air pollution could influence the developing and maturing germ cells in both sexes, and a “fingerprint” of such exposures could be transferred to future offspring and thereby influence their phenotype.

297x209mm (300 x 300 DPI)

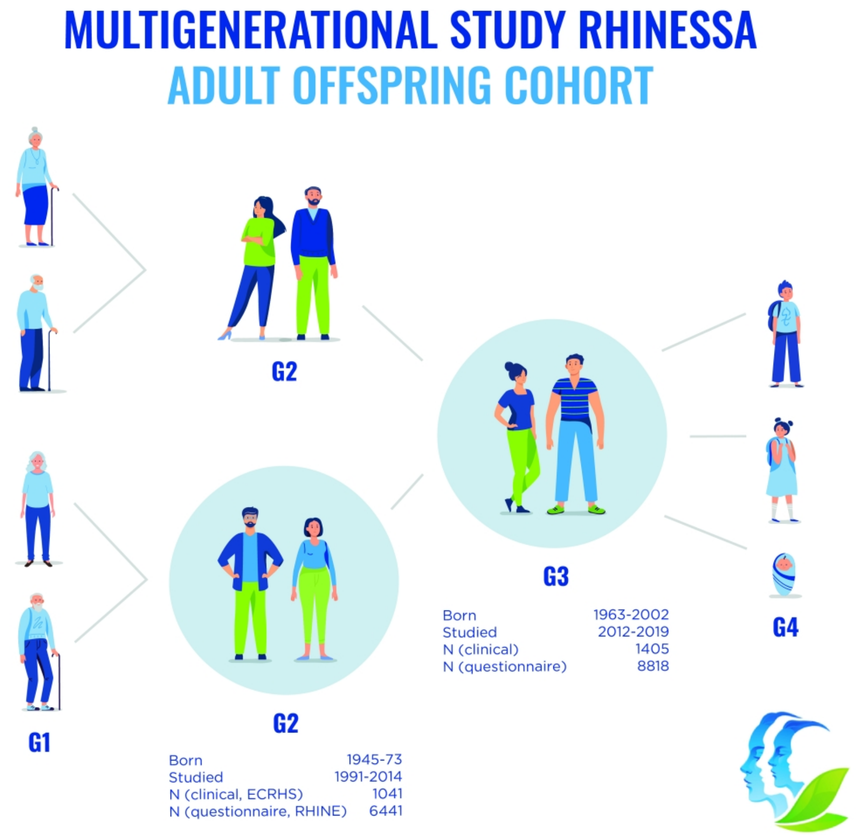

Figure 2. The RHINESSA adult offspring cohort (generation 3 "G3") includes 8818 young men and women investigated with questionnaires (q), of which 1405 were investigated clinically (c). These are the offspring of men and women participating in the RHINE/ECRHS studies (G2) who were followed up over twenty years. In addition, Aarhus, Bergen, Melbourne and Tartu study centres investigated G3 offspring age 4-17 years (1139q/ 201c), and Bergen study centre investigated G1 (1470q/145c), the other G2 parent (910q/152c) and G4 (750q/433c). In all study centres G3 and G2 study participants provided information about their parents and offspring in G1 and G4.

297x280mm (300 x 300 DPI)

SUPPLEMENTARY MATERIAL
Cohort profile: The multi-generation Respiratory Health in Northern Europe, Spain and Australia (RHINESSA) cohort

Supplementary Table 1. Children and adolescents investigated as part of the RHINESSA G3 study (the adult offspring cohort is the main focus of the paper and not presented here).

	Children (4-9 years)		Adolescents (10-17 years)	
	Questionnaire	Clinical	Questionnaire	Clinical
Aarhus			245	6
Bergen	105	44	571	124
Melbourne	13	0	73	11
Tartu			132	16

For peer review only

Supplementary Table 2A. Bergen all generations

	G1	G2 RHINE/ ECRHS parent	G2 other parent	G3 (all age groups)	G4 (all age groups)
Birth years	1918-65	1947-71	1935-88	1965-2012	1988-2008
Study years	2016-18	1991-2012	2016-18	2012-18	2019-21
Numbers, questionnaire study	1470	3452	910	2440	750
Numbers, clinical study	145	835	152	667	433

Supplementary Table 2B. More details about the Bergen 4th generation cohort

	Children (4-9 years)	Adolescents (10-17 years)	Adults (18+ years)	Total	*Response rate (of total)*
Invited, eligible	436	399	138	973	
Participated questionnaire	379	263	108	750	*77%*
Participated clinical	197	163	73	433	*45%*

SUPPLEMENTARY INFORMATION ON THE EUROPEAN COMMUNITY RESPIRATORY HEALTH SURVEY (ECRHS)

ECRHS I

Co-ordinating Centre (London): P. Burney, S. Chinn, C. Luczynska†, D. Jarvis, E. Lai.

Project Management Group: P. Burney (Project leader-UK), S. Chinn (UK), C. Luczynska† (UK), D. Jarvis (UK), P. Vermeire† (Antwerp), H. Kesteloot (Leuven), J. Bousquet (Montpellier), D. Nowak (Hamburg), J. Prichard† (Dublin), R. de Marco† (Verona), B. Rijcken (Groningen), J.M. Antó (Barcelona), J. Alves (Oporto), G. Boman (Uppsala), N. Nielsen (Copenhagen), P. Paoletti (Pisa).

Financial support: The following grants helped to fund the local studies. **Australia**: Asthma Foundation of Victoria, Allen and Hanbury's; **Belgium**: Belgian Science Policy Office, National Fund for Scientific Research; **Estonia:** Estonian Science Foundation, grant no 1088; **France:** Ministère de la Santé, Glaxo France, Institut Pneumologique d'Aquitaine, Contrat de Plan Etat-Région Languedoc-Rousillon, CNMATS, CNMRT (90MR/10, 91AF/6), Ministre Delegué de la Santé, RNSP; **Germany**: GSF, Bundesminister für Forschung und Technologie; **Italy:** Ministero dell'Università e della Ricerca Scientifica e Tecnologica, CNR, Regione Veneto grant RSF n. 381/05.93; **Norway:** Norwegian Research Council project no. 101422/310; **Spain:** Fondo de Investigación Sanitaria (#91/0016-060-05/E, 92/0319 and #93/0393), Hospital General de Albacete, Hospital General Juan Ramón Jiménez, Dirección Regional de Salud Pública (Consejería de Sanidad del Principado de Asturias), CIRIT (1997 SGR 00079) and Servicio Andaluz de Salud; **Sweden:** The Swedish Medical Research Council, the Swedish Heart Lung Foundation, the Swedish Association against Asthma and Allergy; **Switzerland:** Swiss national Science Foundation grant 4026-28099; **UK:** National Asthma Campaign, British Lung Foundation, Department of Health, South Thames Regional Health Authority.

Coordination: The co-ordination of this work was supported by the European Commission; the authors and participants are grateful to the late C. Baya and M. Hallen for their help during the study, and to K. Vuylsteek and the members of the COMAC for their support.

ECRHS II

Steering Committee: U. Ackermann-Liebrich (University of Basel, Switzerland); N. Kuenzli (University of Basel, and University of Southern California, Los Angeles, USA); J.M. Antó and J. Sunyer (Institut Municipal d' Investigació Médica (IMIM-IMAS), Universitat Pompeu Fabra (UPF), Spain); P. Burney (project leader), S Chinn, D. Jarvis, J. Knox and C. Luczynska (King's College London, UK); I. Cerveri (University of Pavia, Italy); R. de Marco† (University of Verona, Italy); T. Gislason (Iceland University Hospital, Iceland); J. Heinrich and M. Wjst (GSF–Institute of Epidemiology, Germany); C. Janson (Uppsala University, Sweden); B. Leynaert and F. Neukirch

(Institut National de la Santé et de la Recherche Medicale (INSERM), France); J. Schouten (University of Groningen, The Netherlands); C. Svanes (University of Bergen, Norway); P. Vermeire† (University of Antwerp, Belgium).

Principal Investigators and senior scientific teams: **Australia: Melbourne** (M. Abramson, E.H. Walters, J. Raven); **Belgium: South Antwerp and Antwerp City** (P. Vermeire, J. Weyler, M. van Sprundel, V. Nelen); **Estonia: Tartu** (R. Jõgi, A. Soon); **France: Paris** (F. Neukirch, B. Leynaert, R. Liard, M. Zureik), **Grenoble** (I. Pin, J. Ferran-Quentin), **Bordeaux** (A. Taytard, C. Raherison), **Montpellier** (J. Bousquet, P.J. Bousquet); **Germany: Erfurt** (J. Heinrich, M. Wjst, C. Frye, I. Meyer); **Iceland: Reykjavik** (T. Gislason, E. Bjornsson, D. Gislason, K.B. *Jörundsdóttir*); **Italy: Turin** (R. Bono, M. Bugiani, P. Piccioni, E. Caria, A. Carosso, E. Migliore, G. Castiglioni), **Verona** (R. de Marco†, G. Verlato, E. Zanolin, S. Accordini, A. Poli, V. Lo Cascio, M. Ferrari, I. Cazzoletti), **Pavia** (A. Marinoni, S. Villani, M. Ponzio, F. Frigerio, M. Comelli, M. Grassi, I. Cerveri, A. Corsico); **Norway: Bergen** (A. Gulsvik, E. Omenaas, C. Svanes, B. Laerum); **Spain: Albacete** (J. Martinez-Moratalla Rovira, E. Almar, M. Arévalo, C. Boix, G. González, J.M. Ignacio García, J. Solera, J. Damián), **Galdakao** (N. Muñozguren, J. Ramos, I. Urrutia, U. Aguirre), **Barcelona** (J.M. Antó, J. Sunyer, M. Kogevinas, J.P. Zock, X. Basagaña, A. Jaen, F. Burgos, C. Acosta), **Huelva** (J. Maldonado, A. Pereira, J.L. Sanchez), **Oviedo** (F. Payo, I. Huerta, A. de la Vega, L. Palenciano, J. Azofra, A. Cañada); **Sweden**: **Göteborg** (K. Toren, L. Lillienberg, A.C. Olin, B. Balder, A. Pfeifer-Nilsson, R. Sundberg), **Umea** (E. Norrman, M. Soderberg, K.A. Franklin, B. Lundback, B. Forsberg, L. Nystrom), **Uppsala** (C. Janson, G. Boman, D. Norback, G. Wieslander, M. Gunnbjornsdottir); **Switzerland: Basel** (N. Küenzli, B. Dibbert, M. Hazenkamp, M. Brutsche, U. Ackermann-Liebrich); **United Kingdom: Ipswich** (D. Jarvis, R. Hall, D. Seaton), **Norwich** (D. Jarvis, B. Harrison).
Financial Support: **Australia:** National Health and Medical Research Council; **Belgium: Antwerp:** Fund for Scientific Research (G.0402.00), University of Antwerp, Flemish Health Ministry; **Estonia: Tartu:** Estonian Science Foundation grant no 4350; **France: (All)** Programme Hospitalier de Recherche Clinique—Direction de la Recherche Clinique (DRC) de Grenoble 2000 number 2610, Ministry of Health, Ministère de l'Emploi et de la Solidarité, Direction Génerale de la Santé, Centre Hospitalier Universitaire (CHU) de Grenoble; **Bordeaux:** Institut Pneumologique d'Aquitaine; **Grenoble:** Comité des Maladies Respiratoires de l'Isere; **Montpellier:** Aventis (France), Direction Regionale des Affaires Sanitaires et Sociales Languedoc-Roussillon; **Paris:** Union Chimique Belge-Pharma (France), Aventis (France), Glaxo France; **Germany: Erfurt:** GSF—National Research Centre for Environment and Health, Deutsche Forschungsgemeinschaft (FR1526/1-1); **Hamburg:** GSF—National Research Centre for Environment and Health, Deutsche Forschungsgemeinschaft (MA 711/4-1); **Iceland: Reykjavik:** Icelandic Research Council, Icelandic University Hospital Fund; **Italy: Pavia:** GlaxoSmithKline Italy, Italian Ministry of University and Scientific and Technological Research (MURST), Local University Funding for Research 1998 and 1999; **Turin:** Azienda Sanitaria Locale 4 Regione Piemonte (Italy), Azienda Ospedaliera Centro Traumatologico Ospedaliero/Centro Traumatologico Ortopedico—Istituto Clinico Ortopedico Regina Maria Adelaide Regione Piemonte; **Verona:** Ministero dell'Università e della Ricerca Scientifica (MURST), Glaxo Wellcome spa; **Norway: Bergen:** Norwegian Research Council, Norwegian Asthma and Allergy Association, Glaxo Wellcome AS, Norway Research Fund; **Spain: Albacete:** Fondo de Investigaciones Sanitarias (97/0035-01, 99/0034-01, and 99/0034-02), Hospital Universitario de Albacete, Consejeria de Sanidad; **Barcelona:** Sociedad Espanola de Neumología y Cirugía Toracica,

For peer review only

Public Health Service (R01 HL62633-01), Fondo de Investigaciones Sanitarias (97/0035-01, 99/0034-01, and 99/0034-02), Consell Interdepartamental de Recerca i Innovació Tecnològica (1999SGR 00241), Instituto de Salud Carlos III, Red de Centros de Epidemiología y Salud Pública (C03/09), Red de Bases moleculares y fisiológicas de las Enfermedades Respiratorias (C03/011), Red de Grupos Infancia y Medio Ambiente (G03/176); **Huelva:** Fondo de Investigaciones Sanitarias (97/0035-01, 99/0034-01, and 99/0034-02); **Galdakao:** Basque Health Department; **Oviedo:** Fondo de Investigaciones Sanitarias (97/0035-02, 97/0035, 99/0034-01, 99/0034-02, 99/0034-04, 99/0034-06, 99/350, and 99/0034-07), European Commission (EU-PEAL PL01237), Generalitat de Catalunya (CIRIT 1999 SGR 00214), Hospital Universitario de Albacete, Sociedad Española de Neumología y Cirugía Torácica (SEPAR R01 HL62633-01), Red de Centros de Epidemiología y Salud Pública (C03/09), Red de Bases moleculares y fisiológicas de las Enfermedades Respiratorias (C03/011), Red de Grupos Infancia y Medio Ambiente (G03/176, 97/0035-01, 99/0034-01, and 99/0034-02); **Sweden: Göteborg, Umea, and Uppsala**: Swedish Heart Lung Foundation, Swedish Foundation for Health Care Sciences and Allergy Research, Swedish Asthma and Allergy Foundation, Swedish Cancer and Allergy Foundation, Swedish Council for Working Life and Social Research (FAS); **Switzerland: Basel:** Swiss National Science Foundation, Swiss Federal Office for Education and Science, Swiss National Accident Insurance Fund; **UK: Ipswich and Norwich**: Asthma UK (formerly known as National Asthma Campaign).

Cordination: The coordination of this work was supported by the European Commission, as part of their Quality of Life programme (QLK4-CT-1999-01237).

ECRHS III

Principal Investigators and senior scientific teams: **Australia: Melbourne** (M. Abramson, G. Benke, S. Dharmage, B. Thompson, S. Kaushik, M. Matheson). **Belgium: South Antwerp & Antwerp City** (J. Weyler, H. Bentouhami, V. Nelen). **Estonia: Tartu** (R. Jõgi, H.Orru). **France: Bordeaux** (C. Raherison, P.O. Girodet), **Grenoble** (I. Pin, V. Siroux, J. Ferran, J.L. Cracowski), **Montpellier** (P. Demoly, A. Bourdin, I. Vachier), **Paris** (B. Leynaert, D. Soussan, D. Courbon, C. Neukirch, L. Alavoine, X. Duval, I. Poirier). **Germany: Erfurt** (J. Heinrich, E. Becker, G. Woelke, O. Manuwald), **Hamburg** (H. Magnussen, D. Nowak, A-M. Kirsten). **Iceland: Reykjavik** (T. Gislason, B. Benediktsdottir, D. Gislason, E.S. Arnardottir, M. Clausen, G. Gudmundsson, L. Gudmundsdottir, H. Palsdottir, K. Olafsdottir, S. Sigmundsdottir, K. Bara-Jörundsdottir). **Italy**: **Pavia** (I. Cerveri, A. Corsico, A. Grosso, F. Albicini, E. Gini, E.M. Di Vincenzo, V. Ronzoni, S. Villani, F. Campanella, M. Gnesi, F. Manzoni, L. Rossi, O. Ferraro), **Turin** (M. Bugiani, R. Bono, P. Piccioni, R. Tassinari, V. Bellisario, G. Trucco), **Verona** (R. de Marco†, S. Accordini, L. Calciano, L. Cazzoletti, M. Ferrari, A.M. Fratta Pasini, F. Locatelli, P. Marchetti, A. Marcon, E. Montoli, G. Nguyen, M. Olivieri, C. Papadopoulou, C. Posenato, G. Pesce, P. Vallerio, G. Verlato, E. Zanolin). **Norway: Bergen** (C. Svanes, E. Omenaas, A. Johannessen, T. Skorge, F. Gomez Real). **Spain: Albacete** (J. Martinez-Moratalla Rovira, E. Almar, A. Mateos, S. García, A. Núñez, P. López, R. Sánchez, E. Mancebo), **Barcelona** (J.M. Antó, J.P. Zock, J. Garcia-Aymerich, M. Kogevinas, X. Basagaña, A.E. Carsin, F. Burgos, C. Sanjuas, S. Guerra, B. Jacquemin, P. Davdand), **Galdakao** (N. Muñozguren, I. Urrutia, U. Aguirre, S. Pascual), **Huelva** (J. Antonio Maldonado, A. Pereira, J. Luis

For peer review only

Sánchez, L. Palacios), **Oviedo** (F. Payo, I. Huerta, N. Sánchez, M. Fernández, B. Robles). **Sweden: Göteborg** (K. Torén, M. Holm, J-L. Kim, A-C. Olin, A. Dahlman-Höglund), **Umea** (B. Forsberg, L. Braback, L. Modig, B. Järvholm, H. Bertilsson, K.A. Franklin, C. Wahlgreen), **Uppsala** (B. Andersson, D. Norback, U. Spetz Nystrom, G. Wieslander, G.M. Bodinaa Lund, K. Nisser). **Switzerland: Basel** (N.M. Probst-Hensch, N. Künzli, D. Stolz, C. Schindler, T. Rochat, J.M. Gaspoz, E. Zemp Stutz, M. Adam, C. Autenrieth, I. Curjuric, J. Dratva, A. Di Pasquale, R. Ducret-Stich, E. Fischer, L. Grize, A. Hensel, D. Keidel, A. Kumar, M. Imboden, N. Maire, A. Mehta, H. Phuleria, M. Ragettli, M. Ritter, E. Schaffner, G.A. Thun, A. Ineichen, T. Schikowski, M. Tarantino, M. Tsai). **UK: London** (P. Burney, D. Jarvis, S. Kapur, R. Newson, J. Potts), **Ipswich** (N. Innes), **Norwich** (A. Wilson).

Financial Support: **Australia:** National Health & Medical Research Council; **Belgium: Antwerp South, Antwerp City:** Research Foundation Flanders (FWO) (G.0.410.08.N.10); **Estonia: Tartu:** Estonian Ministry of Education (SF0180060s09); **France: (All centres)** Ministère de la Santé, Programme Hospitalier de Recherche Clinique (PHRC) national 2010; **Bordeaux:** INSERM U897 Université Bordeaux segalen; **Grenoble:** Comite Scientifique AGIRadom 2011; **Paris:** Agence Nationale de la Santé, Région Ile de France, domaine d'intérêt majeur (DIM); **Germany: Erfurt:** German Research Foundation (HE 3294/10-1); **Hamburg:** German Research Foundation (MA 711/6-1, NO 262/7-1); **Iceland: Reykjavik:** The Landspitali University Hospital Research Fund, University of Iceland Research Fund, ResMed Foundation, California, USA, Orkuveita Reykjavikur (Geothermal plant), Vegagerðin (The Icelandic Road Administration (ICERA). **Italy: (All centres)** Italian Ministry of Health, Chiesi Farmaceutici SpA; **Verona:** Cariverona foundation, Education Ministry (MIUR). **Norway:** Norwegian Research council (214123), Western Norway Regional Health Authorities (911631), Bergen Medical Research Foundation. **Spain:** Fondo de Investigación Sanitaria (PS09/02457, PS09/00716, 09/01511, PS09/02185, PS09/03190), Servicio Andaluz de Salud, Sociedad Española de Neumología y Cirurgía Torácica (SEPAR 1001/2010), Fondo de Investigación Sanitaria (PS09/02457); **Barcelona:** Fondo de Investigación Sanitaria (FIS PS09/00716); **Galdakao:** Fondo de Investigación Sanitaria (FIS 09/01511); **Huelva:** Fondo de Investigación Sanitaria (FIS PS09/02185), Servicio Andaluz de Salud; **Oviedo:** Fondo de Investigación Sanitaria (FIS PS09/03190). **Sweden: (All centres)** The Swedish Heart and Lung Foundation, The Swedish Asthma and Allergy Association, The Swedish Association against Lung and Heart Disease, Swedish Research Council for health, working life and welfare (FORTE); **Göteborg:** Swedish Council for Working life and Social Research; **Umea:** Vasterbotten Country Council ALF grant. **Switzerland:** The Swiss National Science Foundation (33CSCO-134276/1, 33CSCO-108796, 3247BO-104283, 3247BO-104288, 3247BO-104284, 3247-065896, 3100-059302, 3200-052720, 3200-042532, 4026-028099), The Federal office for forest, environment and landscape, The Federal Office of Public Health, The Federal Office of Roads and Transport, the canton's government of Aargan, Basel-Stadt, Basel-Land, Geneva, Luzern, Ticino, Valais, and Zürich, the Swiss Lung League, the canton's Lung League of Basel Stadt/Basel, Landschaft, Geneva, Ticino, Valais, and Zurich, SUVA, Freiwillige Akademische Gesellschaft, UBS Wealth Foundation, Talecris Biotherapeutics GmbH, Abbott Diagnostics, European Commission (018996) (GABRIEL), Wellcome Trust (WT 084703MA). **UK:** Medical Research Council (92091). Support also provided by the National Institute for Health Research through the Primary Care Research Network.

For peer review only

Coordination: The coordination was funded through the Medical Research Council (92091).

† deceased

Ethics approval: Ethics approval was obtained by all centres from the appropriate ethics committees: **Antwerp City and Antwerp South:** Adviescommissie Medische Ethiek UZA-UA (CME); **Tartu:** Research Ethics Committee of the University of Tartu, Estland (N° 209T-17); **French centres:** Comite de protection des personnes, Sud V Est (N° 2011-A00013-38); **German centres:** Ethik-Kommission der Bayerischen Landesarztekammer (N° 10015); **Reykjavik:** National Biotecs Committe of Iceland (NBCI) (N° VSNb2011090016/03.11); **Pavia:** Fondazione IRCCS Policlinico 'San Matteo' (N° P-20110024215); **Turin:** Comitato Etico dell'Azienda Sanitaria Locale TO/2 di Torino (N° 569/09/08); **Verona:** Comitato Etico per la Sperimentazione dell'Azienda Ospedaliera Istituti Ospitalieri di Verona (N° 1393); **Bergen:** Universitetet i Bergen, Regional komité for medisinsk og helsefaglig forskningsetikk, Vest-Norge (REK Vest) (N° 2010/759); **Albacete:** Comité de Ética e Investigación de Complejo Hospitalario de Albacete (N° 04/09); **Barcelona:** Comité Ético de Investigación Clínica del Instituto Municipal de Asistencia Sanitaria, Barcelona, Spain (N° PS09/00716); **Galdakao:** Comité Éticode Investigación del Hospital de Galdakao, Spain (N° 20101104); **Huelva:** Comisión de Investigación del Hospital Juan Ramón Jiménez de Huelva (N° 20090417); **Oviedo:** Comité Ético de Investigación Clínica Regional, Hospital Universitario Central de Asturias (N° 20110415); **Swedish centres:** Ethics Committee at the Medical Faculty, Uppsala University (N° 1999/313 and 2010/068); **Basel:** Swiss Academy of Medical Sciences and the ethics committee of Basel (N° PV123/00,157/00); **UK centres:** NRES Committee London - Stanmore (REC Reference 11/LO/0965 IRAS number 70769).

For peer review only

BMJ Open

Cohort profile: The multi-generation Respiratory Health in Northern Europe, Spain and Australia (RHINESSA) cohort

Journal:	*BMJ Open*
Manuscript ID	bmjopen-2021-059434.R1
Article Type:	Cohort profile
Date Submitted by the Author:	22-Feb-2022
Complete List of Authors:	Svanes, C; University of Bergen, Department of Global Public Health and Primary Care; Haukeland University Hospital, Department of Occupational Medicine<br>Johannessen, Ane; University of Bergen Department of Global Public Health and Primary Care, Centre for International Health<br>Bertelsen, Randi; University of Bergen, Department of Clinical Science; Oral Helath Centre of Expertise Western Norway<br>Dharmage, Shyamali; University of Melbourne, Allergy and Health Unit, School of Population and Global Health<br>Benediktsdottir, Bryndis; University of Iceland, Medical Faculty; Landspitali University Hospital Reykjavík, Department of Sleep<br>Bråbäck, Lennart; Umeå Universitet, Department of Public Health and Clinical Medicine<br>Gislason, Thorarinn; Landspitali University Hospital; Landspitali University Hospital Reykjavík, Department of Sleep<br>Holm, Mathias; University of Gothenburg, Occupational and Environmental Medicine, School of Public Health and Community Medicine, Institute of Medicine, Sahlgrenska Academy<br>Jõgi, Oskar; University of Bergen, Department of Clinical Science; Tartu University Hospital, Lung Clinic<br>Lodge, Caroline J.; University of Melbourne, Allergy and Lung Health Unit, School of Population and Global Health<br>Malinovschi, Andrei; Uppsala University, Department of Medical Sciences: Clinical Physiology<br>Martinez-Moratalla, Jesus; Complejo Hospitalario Universitario de Albacete, Servicio de Neumología; Universidad de Castilla-La Mancha - Campus de Albacete, Facultad de Medicina<br>Oudin, Anna; Umeå Universitet, Section of Sustainable Health, Department of Public Health and Clinical Medicine<br>Sánchez-Ramos, José Luis; University of Huelva, Department of Nursing<br>Timm, Signe; University of Southern Denmark, Department of Regional Health Research; University Hospital of Southern Denmark, Research Unit, Kolding Hospital<br>Janson, Christer; Uppsala University, Department of Medical Sciences: Clinical Physiology; Uppsala University, Department of Medical Sciences: Respiratory, Allergy, Sleep Research<br>Real, Francisco Gomez; University of Bergen, Department of Clinical Science; Haukeland University Hospital, Department of Obstetrics and Gynecology<br>Schlünssen, Vivi; Aarhus Universitet, Department of Public Health - Work, Environment and Health, Danish Ramazzini Centre; National

	Research Centre for the Working Environment
**Primary Subject Heading**:	Epidemiology
Secondary Subject Heading:	Respiratory medicine, Public health
Keywords:	Asthma < THORACIC MEDICINE, Allergy < THORACIC MEDICINE, Chronic airways disease < THORACIC MEDICINE, EPIDEMIOLOGY, PUBLIC HEALTH

SCHOLARONE™
Manuscripts

I, the Submitting Author has the right to grant and does grant on behalf of all authors of the Work (as defined in the below author licence), an exclusive licence and/or a non-exclusive licence for contributions from authors who are: i) UK Crown employees; ii) where BMJ has agreed a CC-BY licence shall apply, and/or iii) in accordance with the terms applicable for US Federal Government officers or employees acting as part of their official duties; on a worldwide, perpetual, irrevocable, royalty-free basis to BMJ Publishing Group Ltd ("BMJ") its licensees and where the relevant Journal is co-owned by BMJ to the co-owners of the Journal, to publish the Work in this journal and any other BMJ products and to exploit all rights, as set out in our licence.

The Submitting Author accepts and understands that any supply made under these terms is made by BMJ to the Submitting Author unless you are acting as an employee on behalf of your employer or a postgraduate student of an affiliated institution which is paying any applicable article publishing charge ("APC") for Open Access articles. Where the Submitting Author wishes to make the Work available on an Open Access basis (and intends to pay the relevant APC), the terms of reuse of such Open Access shall be governed by a Creative Commons licence – details of these licences and which Creative Commons licence will apply to this Work are set out in our licence referred to above.

Other than as permitted in any relevant BMJ Author's Self Archiving Policies, I confirm this Work has not been accepted for publication elsewhere, is not being considered for publication elsewhere and does not duplicate material already published. I confirm all authors consent to publication of this Work and authorise the granting of this licence.

For peer review only

Cohort profile: The multi-generation Respiratory Health in Northern Europe, Spain and Australia (RHINESSA) cohort

Authors

Svanes C[1,2*], Johannessen A[1]*, Bertelsen RJ[3,4], Dharmage SC[5], Benediktsdottir B [6,7], Bråbäck L[8], Gíslason T[6,7], Holm M[9], Jõgi NO[3, 10], Lodge C[5], Malinovschi A[11], Martinez-Moratalla J [12,13], Oudin A[8], Sanchez JL[14,], Timm S [15,16], Janson C[11,17], Gómez Real F[3,18]†, Schlünssen V[19,20]† on behalf of the RHINESSA International Collaboration.

* Shared first authors † Shared last authors

[1] Centre for International Health, Department of Global Public Health and Primary Care, University of Bergen, Bergen, Norway

[2] Department of Occupational Medicine, Haukeland University Hospital, Bergen, Norway

[3] Department of Clinical Science, University of Bergen, Bergen, Norway

[4] Oral Health Centre of Expertise Western Norway, Bergen, Norway

[5] Allergy and Lung Health Unit, School of Population and Global Health, University of Melbourne, Australia

[6] Medical Faculty, University of Iceland, Iceland

[7] Department of Sleep, Landspitali University Hospital Reykjavík, Iceland

[8] Section of Sustainable Health, Department of Public Health and Clinical Medicine, Umeå University, Sweden

[9] Occupational and Environmental Medicine, School of Public Health and Community Medicine, Institute of Medicine, Sahlgrenska Academy, University of Gothenburg, Gothenburg, Sweden

[10] Tartu Universtity Hospital, Lung Clinic, Tartu, Estonia

[11] Department of Medical Sciences: Clinical Physiology, Uppsala University, Uppsala, Sweden

For peer review only

[12] Servicio de Neumología del Complejo Hospitalario Universitario de Albacete, Albacete, Spain.

[13] Facultad de Medicina de Albacate. Universidad de Castilla – La Mancha. Albacete, Spain.

[14] Department of Nursing, University of Huelva, Huelva, Spain

[15] Department of Regional Health Research, University of Southern Denmark, 5230 Odense, Denmark;

[16] Research Unit, Kolding Hospital, University Hospital of Southern Denmark, 6000 Kolding, Denmark

[17] Department of Medical Sciences: Respiratory, Allergy, Sleep Research, Uppsala university, Uppsala, Sweden

[18] Department of Obstetrics and Gynecology, Haukeland University Hospital, Bergen, Norway

[19] Department of Public Health - Work, Environment and Health, Danish Ramazzini Centre, Aarhus University, Denmark

[20] National Research Centre for the Working Environment, Copenhagen, Denmark

Correspondence

Professor Cecilie Svanes, Centre for International Health, Overlege Danielsens Hus, Aarstadveien 21, University of Bergen, 5009 Bergen, Norway.

Telephone +4790892762. E-mail Cecilie.Svanes@uib.no

Word count 3447 words

Key words: RHINESSA; generation study; preconception factors; asthma; allergy; lung function; overweight; environment; occupation; smoking; puberty; non-genetic heredity; anthropometry; chronic diseases

ABSTRACT

For peer review only

For peer review only - http://bmjopen.bmj.com/site/about/guidelines.xhtml

Purpose: The Respiratory Health in Northern Europe, Spain and Australia (RHINESSA) cohort was established to 1) investigate how exposures before conception and in previous generations influence health and disease, particularly allergies and respiratory health, 2) identify susceptible time windows, and 3) explore underlying mechanisms. The ultimate aim is to facilitate efficient intervention strategies targeting multiple generations.

Participants: RHINESSA includes study participants of multiple generations from ten study centres in Norway (1), Denmark (1), Sweden (3), Iceland (1), Estonia (1), Spain (2) and Australia (1). The RHINESSA core cohort, adult offspring generation 3 (G3), was first investigated in 2014-17 in a questionnaire study (N=8818, age 18-53 years) and a clinical study (subsample, n=1405). Their G2 parents participated in the population-based cohorts, European Community Respiratory Heath Survey (ECRHS) and Respiratory Health In Northern Europe (RHINE), followed since the early 1990s when they were 20-44 years old, at 8-10 years intervals. Study protocols are harmonised across generations.

Findings to date: Collected data include spirometry, skin prick tests, exhaled nitric oxide, anthropometrics, bioimpedance, blood pressure; questionnaire/interview data on respiratory/general/reproductive health, indoor/outdoor environment, smoking, occupation, general characteristics and lifestyle; biobanked blood, urine, gingival fluid, skin swabs; measured specific and total IgE, DNA methylation, sex hormones, and oral microbiome. Research results suggest that parental environment years before conception, in particular, father's exposures such as smoking and overweight, may be of key importance for asthma and lung function, and that there is an important susceptibility window in male prepuberty. Statistical analyses developed to approach causal inference suggest that these associations may be causal. DNA methylation studies suggest a mechanism for transfer of father's exposures to offspring health and disease through impact on offspring DNA methylation.

For peer review only

Future plans: Follow-up is planned at 5-8 year intervals, first in 2021-22. Linkage with health registries contributes to follow-up of the cohort.

Strengths and limitations of this study

- The main strength of the RHINESSA cohort is the availability of rich preconception exposure information for a large number of young adolescent and adult study participants, from both the paternal and maternal line, taking advantage of extensive information collected from mothers/fathers over twenty years of childbearing age.
- Excellent health and population registries in the Northern European study centres contribute to unbiased identification of study participants and enrichment of data, and, for some study centres, provide additional information on multiple generations covering cohorts born over the last century.
- The multi-generation design and harmonisation of study protocols across generations provide a valuable opportunity to validate next of kin information, thereby improving the validity of retrospectively collected data on family members.
- The Spanish and Australian study centres contribute to generalisability beyond Northern Europe which has the majority of study participants, however, generalisation to low-income countries must be done with care.
- Weaknesses of RHINESSA further include relatively low response rates, partly mitigated by the opportunity to analyse selection bias based on parental data for responders and non-responders; further, extensive exposure data is only available from one parent in most study centres, while information on the other parent is available from next of kin data reported by the offspring, and from registry data in the Nordic study centres.

For peer review only - http://bmjopen.bmj.com/site/about/guidelines.xhtml

INTRODUCTION

While it is generally acknowledged that intrauterine life and early childhood is essential to health and disease throughout life, emerging evidence supports that there may be important susceptibility windows *before* conception[1-7]. The hypothesis arose from new understanding of epigenetic mechanisms by which environmental effects could be transferred across generations[2, 8-10] and from studies supporting that such transfer of non-mutagenic environmental effects across generations could actually be taking place in humans[11-13]. Theoretically, an exposure affecting one person might at the same time affect that person's germ cells, and thereby the health of future offspring (figure 1). The intrauterine period and male puberty may be time windows when the germ cells are more susceptible to external and internal factors due to extensive epigenetic reprogramming[2, 6, 14].

Knowledge on the early life origins of health and disease led to a paradigm shift in public health strategies, and is today implemented in public health programs targeting mother and child across the globe. The concept of *preconception* origins of health and disease, of susceptible time windows *before* conception, opens a new perspective on public health: Are there opportunities for preventive measures that may result in improved health, not only for the individual but also for their future offspring and generations[15]?

There is a need to establish human generation cohorts that are tailored to investigate the preconception origins of health and disease. Most available literature is based on animal studies. There are human cohort studies with preconception data, such as e.g., the Isle of Wight Studies, the Avon Longitudinal Study of Parents and Children study, the Lifelines NEXT generation study, and the Tasmanian Longitudinal Health Study. However, birth cohort studies often have not collected data from the fathers, or from the childhood/ adolescence of any of the parents. Since the human

For peer review only - http://bmjopen.bmj.com/site/about/guidelines.xhtml

reproductive cycle spans decades, investigating exposure effects from before conception and across generations represents a great challenge.

The RHINESSA study is designed to address this by investigating the offspring of persons who have already been extensively characterized during twenty years of their reproductive life. RHINESSA (**R**espiratory **H**ealth in **N**orthern **E**urope, **S**pain and **A**ustralia) is an international multi-generation multi-centre study established to research the preconception origins of health and disease, in particular allergies and respiratory health. The aims of RHINESSA are to investigate the influence of exposures before conception including in previous generations for health and disease, to identify potentially susceptible time windows for such influences, and to explore mechanisms for exposure effects. RHINESSA's primary focus is allergies and chronic respiratory disorders, namely asthma, rhino-conjunctivitis, allergic sensitization, eczema, chronic obstructive pulmonary disease (COPD), lung function and sleep disorders. The cohort resource and research methodologies of RHINESSA also have the capacity for multi-generation research in other areas, such as obesity, women's health and oral health. The ultimate aim of RHINESSA is to improve health at large by generating a knowledge base for efficient strategies that may improve health over several generations.

COHORT DESCRIPTION

This cohort profile describes the RHINESSA adult offspring cohort (generation 3 (G3) of ≥18 years of age) and their G2 parents investigated as part of the European Community Respiratory Health Survey (ECRHS) and Respiratory Health in Northern Europe (RHINE) studies (figure 2). The on-line supplement gives summary data for younger offspring and additional cohorts (G1-G4) investigated in some study centres - altogether four generations.

Study design

For peer review only - http://bmjopen.bmj.com/site/about/guidelines.xhtml

RHINESSA builds on the large longitudinal studies of respiratory health in adults, the ECRHS, www.ecrhs.org) established in the early 1990's[16-18] and the linked study, RHINE, www.rhine.nu)[19, 20]. For a range of environmental exposures and lifestyle factors, the ECRHS and RHINE cohorts (G2) have data with high time-resolution, both before and during the age of childbearing. The children born to these parents, are the target population of RHINESSA (G3). Bergen RHINESSA also investigated the G1 grandparent generation, the G2 parent not participating in RHINE/ECRHS, and the G4 offspring's offspring. Summary data for these additional cohorts are given in the online supplement (table S2). Northern Europe is well suited for generation studies due to excellent national registries with full coverage of the populations for decades, providing means to identify family members in an unbiased manner as well as information on exposures and outcomes (i.e. (life-time) home addresses for geo-coding, prescription registries for asthma medication). Study centres in Estonia with recent transition from middle- to high-income economy, Spain as a southern European country, and Australia with particularly high allergy prevalence, extend the generalisability of study results beyond Northern Europe where most study centres are situated.

Offspring cohort (G3)

The RHINESSA adult offspring study invited offspring age ≥18 years (G3) of RHINE and ECRHS participants (G2) from ten study centres: Bergen, Norway; Aarhus, Denmark; Uppsala, Göteborg and Umeå, Sweden; Reykjavik, Iceland; Tartu, Estonia; Melbourne, Australia; Huelva and Albacete, Spain (table 1, figure 2). In the Northern European countries all G3 offspring were identified through national registries, for the Spanish and Australian study centres the G3 offspring's contact details were obtained from the G2 parents in ECRHS III (table 1). All offspring with parental questionnaire information (from RHINE or ECRHS) were invited to a questionnaire study. The subsample of these with parental clinical information (from ECRHS) and residing in the study area, were invited to a clinical study (Figure S1). The baseline data collection was performed in all study centres during

For peer review only - http://bmjopen.bmj.com/site/about/guidelines.xhtml

2014-17. The same study protocols (adapted to age) were used in all study centres and all generations, and detailed standard operating procedures (see www.rhinessa.net), interview guides and procedures for translations/back translations contribute to secure harmonisation of data across study centres and generations.

The study centres in Bergen, Aarhus, Tartu and Melbourne also investigated younger G3 offspring <18 years (table S1). Bergen RHINESSA further investigated the G1 grandparent generation, the G2 parent not participating in RHINE/ECRHS, and the G4 offspring's offspring. Summary data for these additional cohorts are given in the online supplement (table S2).

For peer review only

Table 1. Sources of identification of RHINESSA adult participants (18+ years) (G3) and their parents (G2), by centre, including a questionnaire cohort (8818 offspring with their 6441 parents), and a clinical cohort (1405 offspring with their 1041 parents)

	Parents (G2)		RHINESSA adult offspring (G3)	
Study centre	Source used for identifying offspring	N	Source of identification	N included in cohort
**QUESTIONNAIRE COHORT**				
Norway, Bergen	ECRHS I quest. respondents	1250	National registers	1763
Denmark, Aarhus	ECRHS I quest. respondents	974	National registers	1224
Sweden, Uppsala	RHINE III quest. respondents	894	National registers	1314
Sweden, Göteborg	RHINE III quest. respondents	709	National registers	951
Sweden, Umeå	RHINE III quest. respondents	876	National registers	1307
Iceland, Reykjavik	ECRHS I quest. respondents	977	National registers	1245
Estonia, Tartu	ECRHS I quest. respondents	525	National registers	618
Australia, Melbourne	ECRHS III clin. respondents	149	Through the parents	245*
Spain, Huelva	ECRHS III clin. respondents	48	Through the parents	72*
Spain, Albacete	ECRHS III clin. respondents	39	Through the parents	79*
**CLINICAL COHORT**				
Norway, Bergen	ECRHS III clin. respondents	346	National registers	499
Denmark, Aarhus	ECRHS III clin. respondents	68	National registers	83
Sweden, Uppsala	ECRHS III clin. respondents	74	National registers	90
Sweden, Göteborg	ECRHS III clin. respondents	53	National registers	60
Sweden, Umeå	ECRHS III clin. respondents	66	National registers	86
Iceland, Reykjavik	ECRHS III clin. respondents	97	National registers	120
Estonia, Tartu	ECRHS III clin. respondents	159	National registers	195
Australia, Melbourne	ECRHS III clin. respondents	102	Through the parents	144
Spain, Huelva	ECRHS III clin. respondents	38	Through the parents	62
Spain, Albacete	ECRHS III clin. respondents	38	Through the parents	66

For peer review only - http://bmjopen.bmj.com/site/about/guidelines.xhtml

*Parental (G2) information extracted from the ECRHS and harmonized with RHINE questions.

For peer review only

For peer review only - http://bmjopen.bmj.com/site/about/guidelines.xhtml

Table 2. Response rate for RHINESSA adult offspring (18+ years) participants (G3) for the questionnaire cohort and the clinical cohort

Eligible subjects were defined as live subjects 18 years and older with known residential addresses residing in the country (questionnaire cohort) or in/near the study centre (clinical cohort).

	Questionnaire cohort (G3)			*Clinical cohort (G3)*		
Center	Eligible*, N	Included, N	Response rate, %	Eligible*, N	Included, N	Response rate, %
Bergen	4385	1763	40.2	1278	499	39.0
Aarhus	4014	1224	30.5	381	83	21.8
Uppsala, Göteborg, Umeå	8256	3572	42.7	639	236	36.9
Reykjavik	4756	1245	26.2	200	120	60.0
Tartu	2907	618	21.3	669	195	29.1
Melbourne	499	245	49.1	245	144	58.8
Huelva	244	72	29.5	244	66	27.0
Albacete	365	79	21.6	365	62	17.0
Total	**25426**	**8818**	**34.7**	**4021**	**1405**	**34.9**

For peer review only - http://bmjopen.bmj.com/site/about/guidelines.xhtml

Parental cohorts (G2)

In the early 1990's the ECRHS conducted a population based survey among random samples of young adults aged 20-44 years in several European and non-European countries (www.ecrhs.org)[16]. On average 4000 persons (range 1000-7000) from each centre were invited to a postal survey (mean response rate 73%). Clinical examinations were conducted in subsamples from ~45 study centres, primary random subsamples, but for some centres an additional subsample with persons with asthma symptoms. ECRHS followed up the clinical samples in 30 study centres in 2002-04 (ECRHS II)[17] and 2012-15 (ECRHS III)[18].

The RHINE study (www.rhine.nu) developed protocols to follow up responders to the initial ECRHS postal survey in seven Northern European centres: Bergen, Norway; Göteborg, Umeå and Uppsala, Sweden; Aarhus, Denmark; Reykjavik, Iceland and Tartu, Estonia. In 2000-2002, 16106 persons answered extensive postal questionnaires (RHINE II, mean response rate 75%)[19, 20]. The population was reinvestigated in 2010-12, with 13093 answering a postal questionnaire. Analyses of non-response showed only minor differences between long-term participants and baseline participants in exposure-outcome associations between age, gender, smoking and respiratory symptoms[19].

Follow-up

Regular follow-up of the RHINESSA clinical and questionnaire cohorts is planned to take place with 5-8 years intervals. The first follow-up of the full cohort is about to start in all study centres in 2021-22. An *ad hoc* clinical follow-up was performed in Bergen study centre in 2020 to capture features related to the Covid-19 pandemic. The parent populations of all study centres have been followed with 8-10 year intervals since the 1990's, and the fourth study waves of RHINE and ECRHS are taking place in 2021-22.

For peer review only - http://bmjopen.bmj.com/site/about/guidelines.xhtml

Ethical consideration

Ethical permissions were obtained for each study wave from the local ethics committee in each of the participating centres. The ethical approval reference numbers are listed on www.rhinessa.net. All study participants provided written informed consent prior to participation. Permission to extract information about themselves and family members from national registers were obtained from each participant in the Northern European study centres. For children and adolescents participating in the additional study groups presented in the online supplementary, written informed consents were given by the parents/guardian, as required by the local ethics committees.

Response rate and parental characteristics related to offspring response

Identified offspring were sent an invitation letter with information about the study and log-in details to a web-based questionnaire, two reminders were sent, in some centres including a postal questionnaire. Persons eligible to a clinical study, were invited by a letter and/or contacted by telephone to agree on an appointment for clinical investigation, also with two reminders. For the three Swedish study centres, the researchers were not allowed to identify and approach study participants directly and Statistics Sweden distributed the invitation letters to participants of both the questionnaire and the clinical study. Altogether 8818 persons participated in the questionnaire cohort and 1405 of these in the clinical cohort (table 1). The overall response rate was 35% both for the questionnaire and the clinical cohort, with differences between study centres and between the questionnaire and clinical stages (table 2). Reasons for non-participation included inability to make contact with the persons due to erroneous contact details or because the person was no longer living at that address, as well as unwillingness or inability to participate. However, parental characteristics were compared between the responders and the source parental population (table 3), showing fairly similar characteristics and no clear patterns of differences, e.g. approximately 55% had a father or mother who had ever smoked in both groups, and there was e.g. no clear trend of higher response

For peer review only - http://bmjopen.bmj.com/site/about/guidelines.xhtml

rates among offspring of symptomatic parents. As expected due to the original sampling strategy in ECRHS (enriched with persons with symptoms) the prevalence of asthma is somewhat higher in the clinical sample compared to the questionnaire sample.

For peer review only

Table 3. Parental (G2) characteristics for RHINESSA (G3) adult (18+ years) responders compared to the source parental RHINE/ECRHS population (G2) for the questionnaire cohort and the clinical cohort

	Questionnaire cohort		*Clinical cohort*	
	RHINE/ECRHS parents (G2) to RHINESSA adult offspring (G3) N = 6441	All RHINE (G2) N = 13260	RHINE/ECRHS parents (G2) to RHINESSA adult offspring (G3) N = 1041	All* ECRHS (G2) N = 3205
**Paternal (G2) characteristics**				
Ever smoker, %	53.2	54.6	55.9	55.3
Primary school	17.1	11.9	14.2	12.1
Secondary school	37.1	44.3	38.1	45.4
College/University	45.8	43.8	47.7	42.5
BMI (SD)	26.8 (4)	26.8 (4)	27.8 (4)	27.6 (4)
Overweight in puberty**, %	9.2	10.3	9.9	10.9
Asthma, %	12.3	10.3	23.7	17.6
Wheeze, %	20.1	20.7	28.6	27.1
**Maternal (G2) characteristics**				
Ever smoker, %	54.0	54.0	45.1	52.1
Primary school	16.2	10.9	16.4	12.3
Secondary school	33.9	39.5	33.9	38.2
College/University	49.9	49.6	49.7	49.5
BMI (SD)	25.7 (5)	25.6 (5)	27.0 (5)	27.0 (5)
Overweight in puberty**, %	23.9	24.3	23.5	23.5
Asthma, %	14.3	13.6	23.1	26.4
Wheeze, %	20.2	19.3	24.4	27.7

* Only including data for the 10 study centres in RHINESSA.
** Overweight defined by self-reported body silhouette at age of menarche / age of voice break[21]

For peer review only - http://bmjopen.bmj.com/site/about/guidelines.xhtml

Collected data and characteristics of study participants

Data and biomaterial collected in RHINESSA include questionnaire/interview information on respiratory and general health, life style and environmental exposures; measurements of lung function, anthropometrics, blood pressure; allergy markers, sex hormones, DNA methylation, and biomarkers in urine and dust samples. Table 4 displays questionnaire/interview data, clinical measures, samples, and measured biomarkers that is available in the RHINESSA adult (18+ years) population (G3), as well as information that has been collected from/about their parents (G2), their grandparents (G4) and their offspring (G1). In addition, national health registries in the Nordic countries with excellent coverage provide an additional data source for the generations G1-G4 and their family members. Some registries date back to the 18th century, while there are most registry data available the last decades.

For peer review only

Table 4. Key data available for the RHINESSA adult offspring (18+ years), and their grandparents, parents and offspring

For the G2 generation, information from three study waves are presented. (*x available for all; ss available in subsample– see footnotes*)

	Grandparents (G1) born 1898-1965	Parents (G2) at 20-44 years RHINE/ECRHS I	Parents (G2) at 30-54 years RHINE/ECRHS II	Parents (G2) at 40-64 years RHINE/ECRHS III	RHINESSA (G3) adult offspring 18-53 years*	Offspring's (G4) offspring age 0-30 years
**Questionnaire/interview**						
Social class, education	x	x	x	x	x	ss
Childhood factors	x	x	x	x	x	ss
Birth characteristics**		ss			ss	ss
Place of upbringing	x	x	x	x	x	ss
Smoking	x	x	x	x	x	ss
Snus, oral moist tobacco, E-cigarettes					x	ss
Occupation	ss	x	x	x	x	ss
Indoor environment	ss	x	x	x	x	ss
Personal care products	ss			x	x	ss
Height/weight	ss	x	x	x	x	ss
Body shapes	x			x	x	ss
Waist circumference (self-measured)				x		
Physical activity	ss	ss	x	x	x	ss
Diet	ss	ss	ss	x	x	ss
Allergic diseases / symptoms	x	x	x	x	x	x
Asthma / symptoms	x	x	x	x	x	x
Asthma/ allergy treatment	ss	x	x	x	x	ss
Sleep	ss	ss	ss	x	x	ss
Other diseases/ symptoms	x	x	x	x	x	ss
Quality of life SF-36 /RAND	ss		ss	ss	ss	ss
Work disability			ss	x	x	
Air pollution and greenness***				ss	ss	
**Women questionnaire/interview** (from women in each cohort)						
Pregnancies and complications			x	x	x	ss
Birth characteristics of offspring			x	x	x	ss
Menarche, menstrual data, menopause			x	x	x	ss
Exogeneous sex hormones			x	x	x	ss
Irregular menstruation, PCOS			x	x	x	ss
Gynecological and related diseases			x	x	x	ss
**Clinical measures** (from clinical stage in each cohort)						
Anthropometry (height/weight/waist/hip)	ss	x	x	x	x	ss
Bioimpedance	ss			x	ss	ss

For peer review only

Spirometry (FEV1, FVC)	ss	x	x	x	x	ss
Post BD spirometry	ss			x	x	ss
Metacholine test		x	x			
FeNO	ss			x	x	ss
Skin prick test	ss	x		x	x	ss
Blood pressure	ss			x	x	ss
Heart rate	ss			x	x	ss
CIMT (carotis intima media)					ss	
CPI/caries index				ss	ss	
**Biological material and environmental samples** (from clinical stage in each cohort)						
Blood samples	ss	x	x	x	x	ss
Gingival samples	ss			ss	ss	ss
Skin swab	ss				ss	ss
Saliva	ss				ss	ss
Urine	ss	ss		x	x	ss
Bedroom dust samples	ss		ss	ss	ss	ss
**Biomarkers measured/funded at time of publication** (from clinical stage in each cohort)						
Total and specific IgEs	ss	x	x	x	x	ss
Genome wide genotyping			ss			
Selective genotyping			ss			
DNA methylation in fullblood	ss		ss	ss	ss	
Fasting blood glucose	ss			ss	ss	ss
Sex hormones			women	women	ss	ss
Oral microbiome	ss				ss	
Urine biomarkers of chemical exposures					ss	
Complete blood cell counts					ss	
Adipokines			ss			

* Sweden and Iceland did a shorter clinical protocol of RHINESSA adult offspring, not including bioimpedance, skin swap or saliva (except that Uppsala collected saliva). RHINESSA offspring <18 years were included in Aarhus, Bergen, Melbourne and Tartu, following age-adapted slightly shorter protocols, similar to protocols used for corresponding age groups in offspring's offspring.
** Information from registries and hospital protocols, and from mothers
*** Information generated using geo-coding based on registry data on life-time addresses

<u>Subsamples:</u>
Grandparents and offsprings' offspring were only investigated in Bergen, information in other centres are given by family members.
CIMT and CPI were only measured in Bergen.
Gingival samples were collected in parents and offspring from Bergen, Melbourne and Tartu, and in offspring from Uppsala.
DNA methylation was measured in fullblood using the Illumina EPIC BeadChip arrays in approximately 900 offspring, 400 parents and 140 grandparents.
Sex hormones were measured in mothers and approx. 1000 offspring from all centres.

For peer review only

For peer review only - http://bmjopen.bmj.com/site/about/guidelines.xhtml

Oral microbiome was measured using 16S rRNA Illumina MiSeq in Bergen adult/adolescent offspring and grandparent.
Urine biomarker concentrations of chemical exposure was measured in Bergen adult / adolescent offspring.
Complete blood cell counts were measured in Swedish centres, adipokines also in Reykjavik.
Helminth serology was measured in offspring from Bergen, Tartu and Aarhus, and parents from Bergen.

For peer review only

Tables 5a and 5b displays characteristics of the study population by study centre, separately presented for the questionnaire study population (5a) and the clinically investigated subsample (5b). Mean age at baseline was 30.1 years and there were 58% women, 33% had ever smoked and 21% had ever used oral moist tobacco (0.8% in Aarhus, 33.9% in Umeå). Asthma medication was used at the time of study by 8.7%, ranging from 6.5% in Aarhus to18.9% in Melbourne (table 5). The proportion of missing data ranges from <0.01 to 4.2% for key variables presented in tables 5a and b.

For peer review only

For peer review only - http://bmjopen.bmj.com/site/about/guidelines.xhtml

Table 5. Characteristics of the RHINESSA adult offspring (18+ years) cohorts by centre; a) questionnaire cohort (N=8818), and b) clinical cohort (N=1405)

a)

	Bergen	Aarhus	Uppsala	Göteborg	Umeå	Reykjavik	Tartu	Melbourne	Huelva	Albacete	*Total*	*Missing, %*
Age (mean, SD)	29.2 (7.4)	27.0 (7.4)	30.4 (7.6)	31.5 (8.0)	32.0 (7.5)	32.0 (8.1)	28.6 (6.2)	28.9 (6.5)	32.5 (7.0)	30.6 (7.1)	30.1 (7.7)	*0.4*
Sex, % females	57.8	59.8	56.3	52.9	57.0	62.7	58.4	53.7	62.5	52.6	57.8	*<0.1*
BMI (mean, SD)	24.3 (4.3)	23.7 (4.3)	24.0 (4.2)	24.5 (4.3)	24.6 (4.4)	26.2 (5.1)	23.8 (4.5)	23.7 (4.8)	24.2 (4.1)	23.9 (5.2)	24.4 (4.5)	*3.2*
Ever smoker, %	36.5	30.3	29.5	35.1	26.0	38.3	38.0	31.2	41.7	55.1	33.3	*1.9*
Ever used oral moist tobacco[a], %	29.6	4.1	23.1	24.3	33.9	15.7	9.2	0.8	N/A	N/A	20.5	*0.5*
Current smoker, %	12.7	15.2	8.8	14.4	7.6	14.1	21.5	13.9	33.3	32.1	13.0	*2.0*
Domestic ETS in childhood, %	54.8	50.8	37.0	49.3	43.5	61.2	55.8	24.6	54.2	63.6	49.4	*3.7*
Educational level:												*2.0*
Primary school, %	2.6	2.1	2.5	2.4	2.2	5.3	7.2	0.0	1.4	6.6	3.1	
Secondary educ. %	35.9	43.3	37.3	45.3	42.4	33.1	38.2	22.2	40.3	32.9	38.5	
College, univ. %	61.5	54.6	60.2	52.3	55.4	61.6	54.6	77.8	58.3	60.5	58.4	
Childhood asthma (onset <10 years), %	6.3	5.9	6.4	5.0	7.9	10.8	3.6	25.0	13.9	3.9	7.4	*1.1*
Current asthma medication, %	7.6	6.5	9.9	8.1	11.4	8.6	3.9	18.9	9.7	14.3	8.7	*<0.1*
Current hay fever / nose allergy, %	28.9	25.7	29.6	27.9	26.9	32.2	27.3	47.3	36.1	35.1	29.1	*0.4*
Childhood atopic dermatitis (onset <10 years), %	6.7	8.5	8.9	9.7	8.0	10.3	5.3	9.8	0.0	3.9	8.2	*4.0*
Current atopic dermatitis, %	8.6	8.2	13.0	11.3	10.0	14.5	11.0	11.1	5.6	12.8	10.8	*<0.1*

[a] Not available information on e-cigarettes in centres labelled N/A

[b] Defined as ever having had itchy rash that was coming and going for at least 6 months, and that the rash affected any of the following places: the fold of the elbows, behind the knees, in front of the ankles, under the buttocks or around the neck, ears or eyes.

For peer review only

For peer review only - http://bmjopen.bmj.com/site/about/guidelines.xhtml

b)

	Bergen	Aarhus	Uppsala	Göteborg	Umeå	Reykjavik	Tartu	Melbourne	Huelva	Albacete	*Total*	*Missing %*
Age (SD)	28.0 (6.6)	28.2 (8.2)	31.4 (7.8)	31.3 (7.5)	31.2 (7.3)	34.6 (8.1)	29.8 (5.8)	29.1 (6.5)	32.2 (7.1)	31.0 (7.7)	29.9 (7.2)	*0.2*
Sex, % females	47.3	62.2	68.9	55.2	55.3	56.8	44.3	53.2	63.6	47.5	52.1	*0.2*
BMI (SD)	25.1 (4.5)	24.4 (5.0)	25.3 (5.5)	24.6 (3.5)	25.2 (4.4)	28.1 (5.1)	24.9 (5.0)	25.0 (4.5)	24.3 (4.5)	24.2 (5.0)	25.2 (4.8)	*0.3*
Ever smoker, %	29.9	25.3	30.0	30.0	17.4	45.0	38.5	26.4	43.9	51.6	32.6	*0.6*
Current smoker, %	13.9	16.9	4.5	5.0	1.2	9.2	19.5	6.9	28.8	29.0	13.4	*0.2*
Domestic ETS in childhood[a], %	54.2	44.6	N/A	N/A	N/A	N/A	53.9	36.8	53.0	75.8	52.2	*0.8*
Childhood asthma, onset <10 years, %	4.4	8.4	6.7	3.5	5.8	10.9	2.1	19.4	9.2	3.2	6.8	*1.1*
Current asthma medication, %	4.6	3.6	8.9	0.0	5.8	2.5	1.5	18.1	6.2	6.5	5.6	*3.1*
Current hay fever/ nose allergy, %	31.5	31.3	38.2	50.0	37.7	29.2	26.7	51.4	31.8	29.0	34.1	*3.7*
Childhood atopic dermatitis, onset <10 years) [b], %	5.4	2.4	5.6	3.3	4.7	9.2	5.1	20.1	3.0	1.6	6.6	*0.9*
Current atopic dermatitis, %	7.2	6.0	10.1	10.0	5.8	14.3	3.6	16.0	6.1	3.2	8.1	*0.1*
FEV_1 l (SD)	3.91 (0.8)	3.80 (0.7)	3.56 (0.7)	3.77 (0.7)	3.72 (0.7)	3.64 (0.8)	4.08 (0.8)	3.78 (0.8)	3.51 (0.7)	3.63 (0.6)	3.83 (0.8)	*1.6*
FVC l (SD)	4.80 (1.1)	4.65 (0.9)	4.42 (0.9)	4.70 (0.9)	4.73 (0.9)	4.60 (1.0)	4.97 (1.0)	4.69 (0.9)	4.29 (0.9)	4.28 (0.8)	4.73 (1.0)	*1.9*
FEV_1/FVC (SD)	0.82 (0.1)	0.82 (0.1)	0.81 (0.1)	0.80 (0.1)	0.79 (0.1)	0.79 (0.1)	0.82 (0.1)	0.81 (0.1)	0.82 (0.1)	0.85 (0.1)	0.81 (0.1)	*2.0*
FEV_1 % pred[c]. (SD)	95 (11)	94 (10)	94 (13)	93 (11)	93 (10)	94 (12)	97 (11)	95 (13)	96 (11)	95 (11)	95 (11)	*4.2*
FVC % pred[c]. (SD)	98 (11)	97 (10)	97 (12)	96 (10)	98 (10)	98 (11)	98 (10)	99 (11)	97 (10)	93 (12)	98 (11)	*4.2*
FEV_1/FVC% pred (SD)	97 (7)	96 (6)	96 (7)	96 (6)	94 (6)	96 (6)	98 (7)	95 (7)	98 (7)	102 (7)	97 (7)	*4.2*

[a] Not available information on ETS exposure in centres labelled N/A

[b] Defined as ever having had itchy rash that was coming and going for at least 6 months, and that the rash affected any of the following places: the fold of the elbows, behind the knees, in front of the ankles, under the buttocks or around the neck, ears or eyes.

[c] Calculated based on Global Lung function Initiative GLI2012 reference values (Quanjer et al, ERJ 2012)

Definitions of asthma, hay fever/ nose allergies and atopic dermatitis are the same as in the questionnaire study presented above in Table 2a, but based on information obtained by standardised interviews rather than self-filled in questionnaires.

The presented lung function data refer to pre-bronchodilator measurements.

For peer review only - http://bmjopen.bmj.com/site/about/guidelines.xhtml

Participant and public involvement

User representatives from the Norwegian Asthma and Allergy Foundation and the Norwegian Labour Inspection have been involved in the RHINESSA Advisory Board from the establishment of the study, and have contributed to development of the study as well as discussions of priorities in analyses and publication of data. One study participant has at a later stage been included in the RHINESSA Advisory Board, as user representative of the study population. Information on the research is available to the study participants through the study website and newsletters. Field workers are alert to comments from the study participants regarding the burden of the study and convey these experiences in annual meetings.

FINDINGS TO DATE

A summary of key findings to date is provided in Table 6.

Smoking and overweight in male prepuberty and offspring health

An explorative analysis of asthma in >24.000 offspring of the RHINE cohort[22], suggested that father's smoking before conception was associated with asthma in future offspring. Mother's smoking around the time of pregnancy, but not before conception, and paternal grandmother's smoking were further associated with offspring asthma. A multi-generation analysis of the ECRHS cohort[23] by Accordini et al. confirmed effects of father's pre-pubertal smoking, using advanced statistical mediation modelling to account for the complexity in the multi-centre multi-generation data, including simulation analyses showing that the impact of unmeasured confounding on the estimates was limited[24, 25]. State-of-the-art statistical methods for causal inference from observational data[26] were applied in a subsequent analysis of the RHINESSA/ECRHS cohorts, suggesting that father's smoking <15 years caused lower lung function in offspring[27]. Effects of father's smoking across generations are supported by preliminary mechanistic work, including a study by Knudsen TM et al. showing that father's smoking was associated with specific DNA methylation patterns in

For peer review only - http://bmjopen.bmj.com/site/about/guidelines.xhtml

adult offspring[28], and a murine study by Hammer et al. uncovering that preconception smoke exposure altered miRNAs in the spermatozoa, and gave higher postnatal body weight in progeny[29].

Further support for early male puberty as an important susceptibility window, was revealed by Johannessen et al. showing that father's onset of overweight between age 8 years and voice break was associated with asthma in future offspring[21]. An ongoing analysis by Lønnebotn et al. suggests that father's prepubertal overweight also may cause lower lung function in offspring[30]. Investigating ***overweight as outcome***, Knudsen et al. demonstrated that father's prepubertal smoking onset was associated with excessive fat mass in their future sons[31]. Johannessen et al. showed that father's and mother's overweight in childhood, and mother's overweight at menarche, were associated with offspring overweight in childhood[21].

Other preconception exposures in mothers and fathers and offspring health

Bertelsen et al. found that ***parental asthmatic and allergic disease activity*** *before* conception was more strongly associated with offspring allergic asthma, than parental disease activity *after* the child was born[32]. The identified pattern might possibly reflect an influence of asthmatic/allergic disease activity on germline cells and thereby on future offspring phenotype. A study of ***parental immune response to helminths*** in Norway by Jõgi et al. uncovered that IgG4 to the zoonotic helminth *Toxocara* in parents was associated with allergic symptoms in their offspring, following a sex-specific pattern[33]. Timm et al. explored whether ***farm upbringing in previous generations*** could influence offspring asthma and allergies, and found no evidence of an association between parental/grandparental farm upbringing and offspring asthma[34]. Regarding selective migration which has not previously been studied across three generations, an analysis suggested that asthma in the family was not a risk factor for quitting farming[35]. Regarding ***parental occupational exposures***, Svanes et al. found that father's welding ≥10 years before conception was associated with a doubled

For peer review only - http://bmjopen.bmj.com/site/about/guidelines.xhtml

risk of asthma in future offspring[22]. Pape et al. investigated four groups of exposures defined from an asthma-specific job exposure matrix (JEM), and compared exposure only before conception with exposure starting before conception and continuing. Associations with offspring asthma were not identified for most exposure groups, except higher risk of early-onset asthma for mothers' exposure to "allergens and reactive chemicals" before and after the offspring's birth[36]. Tjalvin et al. investigated the specific exposure category "indoor cleaning agents: cleaning products/detergents and disinfectants", present in jobs codes such as nurses, personal care workers, cooks and cleaner[37]. Exposure starting before conception was associated with higher asthma risk in offspring, while there was no association with exposure starting after birth. Kuiper et al.[38] analysed ***parental air pollution in*** childhood/adolescence as related to offspring asthma and hayfever. Data on various air pollutants in parents from 1975 onwards were generated by geocoding of parental individual residential addresses obtained from national registries. Mother's PM10 exposure before age 18 years had a direct effect with doubled asthma risk in offspring, and father's ozone exposure in the same age window was associated with increased offspring hayfever risk[38].

Heritability in symptoms and diseases across generations

In a study of ***sleep disturbances***, Lindberg et al. showed that sleep-related symptoms and sleep duration were more common in offspring whose parents had reported the same symptom, consistent after adjusting for lifestyle factors, education and parity[39]. Ekström et al. found that ***breathlessness*** was nearly doubled in offspring of parents with breathlessness, even when adjusting for factors associated with breathlessness in both generations (obesity, smoking, depression, asthma, lower lung function and female sex)[40]. Carsin et al. found that ***grandfather's cardiometabolic disease*** (CMD) was directly associated with grand offspring asthma, while accounting for indirect effects transmitted through parental CMD or asthma, consistently in the RHINE, ECRHS cohorts and RHINESSA cohorts (not yet published).

For peer review only - http://bmjopen.bmj.com/site/about/guidelines.xhtml

Validation studies informing multi-generation epidemiological research

Information about family members is often sought from study participants, as this is cost-effective and may be the only feasible way to obtain the information. The RHINESSA/RHINE/ECRHS cohorts provide an important opportunity for validation of such next of kin information. Kuiper et al. found moderate to good agreement between self-reported asthma and asthma reported by family members, both regarding offspring asthma reported by parents and vice versa[41]. The reporting was better for childhood onset *versus* later onset asthma. Pape et al. found that adults reported quite accurately their parents' smoking during their childhood and their mother' smoking when pregnant with them, when compared with the parents' own report[42]. Timm et al. found that the accuracy in reporting parental place of upbringing was dependent on own place of upbringing, but this did not bias the associations of place of upbringing with asthma[43]. Skulstad et al. validated mothers' information about births and pregnancies against data from the Medical Birth Registry of Norway. The analysis found high validity for mother's report of important birth and pregnancy parameters, and that risk-associations were similar when using maternal *versus* registry based information[44].

Life course data on obesity is rarely available for multiple generations. The RHINESSA/RHINE/ECRHS studies have included a tool with pictorial drawings of body silhouettes in childhood, voice break/menarche and adult ages. Dratva et al. found that current body silhouettes were highly correlated with BMI calculated from measured or self-reported weight and height[45]. Lønnebotn et al. found that retrospective body silhouettes from adult ages correlated well with BMI calculated from measured height and weight at the corresponding ages in the past, and allowed for differentiation of obesity and non-obesity[46].

For peer review only - http://bmjopen.bmj.com/site/about/guidelines.xhtml

Table 6. Overview of key publications from RHINESSA/RHINE/ECRHS on preconception exposures as related to offspring respiratory outcomes, phenotypes across generations and validation studies relevant for multi-generation research

Exposure	Outcome	Exposure window	Main findings	Study cohorts	Reference
Smoking					
Smoking	Non-allergic early onset asthma	Paternal prepuberty: paternal grandmother's pregnancy	Fathers smoking in prepuberty associated with asthma in his offspring, in absence of grandmothers smoking during the father's pregnancy.	RHINE	Svanes et al. Int J Epidemiol, 2017[22]
Smoking	Allergic and non-allergic asthma	Paternal prepuberty; pregnancy	Fathers smoking in prepuberty associated with non-allergic asthma in his offspring; grandmothers smoking during mother's fetal period associated with allergic asthma in her grandchild.	ECRHS	Accordini et al. Int J Epidemiol, 2018[23]
Smoking	Lung function	Paternal prepuberty; grand-maternal pregnancy	Fathers smoking in prepuberty reduced offspring's FEV_1 and FVC; the grandmothers smoking during the father's fetal period reduced the grandchild's FEV_1/FVC.	Parents: ECRHS Offspring: RHINESSA	Accordini et al. Eur Respir J, 2021[27]
Occupational exposures					
Welding	Non-allergic asthma	Paternal adolescence	Fathers' pre-conception welding was associated with non-allergic asthma in offspring.	RHINE	Svanes et al. Int J Epidemiol, 2017[22]
Allergens, reactive chemicals, microorganisms and pesticides	Asthma	Before conception of child; pre- and post-conception combined	Preconception maternal and paternal exposure to occupational agents not associated with asthma in offspring, expect higher early-onset asthma with mother exposure to allergens and/or reactive chemicals before and after conception	Parents: ECRHS Offspring: RHINESSA	Pape et al. Int Epidemiol, 2020[36]
Cleaning products and disinfectants	Asthma and/or wheeze	Before conception of child; around conception and pregnancy	Mother's exposure to indoor cleaning starting before conception was associated with offspring's childhood allergic and non-allergic asthma.	Parents: RHINE Offspring: RHINESSA	Tjalvin et al. J Allergy Clin Immunol, 2021[37]
Environmental exposures					
Air pollution	Asthma and allergies	Parental childhood	Parental exposure to air pollution during childhood increased the risk of asthma and allergies in offspring.	RHINESSA	Kuiper et al. Int. J Environ Res. Pub Health 2020[38]
Farm exposure	Asthma	Parental childhood	Farm upbringing in previous generations was not associated with offspring asthma – either for parental or grandparental upbringing.	Parents: ECRHS/RHINE Offspring: RHINESSA	Timm et al. Int J Epidemiol, 2020[34]
Metabolic and hormonal exposures					

For peer review only - http://bmjopen.bmj.com/site/about/guidelines.xhtml

Overweight and weight gain	Non-allergic asthma	Paternal puberty	Paternal overweight and weight gain before puberty associated with offspring non-allergic asthma.	Parents: ECRHS/RHINE Offspring: RHINESSA	Johannessen et al. JACI, 2020[21]
Overweight	Lung function	Paternal childhood/puberty	Paternal overweight during childhood and/or puberty may cause lower lung function in offspring.	Parents: ECRHS Offspring: RHINESSA	Lønnebotn et al. Eur Respir J, 2020[30]
Infections and disease processes					
Helminth infection	Allergies	Not known	*Toxocara spp* seropositivity in parents was associated with allergic outcomes in their offspring.	Parents: ECRHS Offspring: RHINESSA	Jogi et al. Clin Exp Allergy, 2018[33]
Bronchial hyper-responsiveness and level of specific IgEs	Asthma and allergies	Before conception of child	Parental asthmatic and allergic disease activity measured before conception was associated to offspring asthma and hay fever.	ECRHS	Bertelsen et al. Clin Exp Allergy, 2017[32]
**Phenotype across generations**					
Sleep characteristics		Sleep-related symptoms and sleep duration more common in offspring with same outcome in parents, after adjusting for lifestyle factors, education and parity in both generations		Parents: ECRHS/RHINE Offspring: RHINESSA	Lindberg et al. Sleep Med 2020[39].
Breathlessness		Breathlessness nearly doubled in offspring of parents with breathlessness, after adjusting for obesity, smoking, depression, asthma, lower lung function and female sex in both generations		Parents: ECRHS/RHINE Offspring: RHINESSA	Ekstrøm et al. Thorax 2021[40].
**Validation studies**					
Asthma reported by family members		Moderate to good agreement between self-reported asthma and asthma reported by family members, for offspring asthma reported by parents and vice versa, better fr childhood than adult onset asthma.		Parents: ECRHS/RHINE Offspring: RHINESSA	Kuiper et al. BMC Pulm Med[41].
Parental smoking reported by offspring		Adults reported well their parents' smoking during their childhood and their mother' smoking when pregnant with them, when compared with the parents' own report.		Parents: ECRHS/RHINE Offspring: RHINESSA	Pape et al.BMC Public Health 2018[42].

For peer review only - http://bmjopen.bmj.com/site/about/guidelines.xhtml

Parents' place of upbringing reported by offspring	Offspring report of parents' place of upbringing was dependent on own place of upbringing, this did not bias the associations of place of upbringing with asthma[43].	Parents: ECRHS/RHINE Offspring: RHINESSA	Timm et al. Epidemiology 2019[43].
Birth characteristics reported by mothers	High validity for mother's report of birth and pregnancy parameters. Risk-associations were similar when using maternal *versus* registry based information.	Bergen RHINE, Medical Birth Registry of Norway	Skulstad et al. PlosOne 2017[44].
Current body silhouettes validated against measured and reported height/ weight	Current body silhouettes were highly correlated with BMI calculated from either measured or self-reported weight and height.	ECRHS, RHINE	Dratva et al. Pub Health Nutr 2016 [45].
Retrospective body silhouettes validated against previously measured and reported height/ weight	Retrospective body silhouettes from adult ages correlated well with BMI calculated from measured height/weight at corresponding ages in the past, and allowed differentiation of obesity and non-obesity	ECRHS, RHINE	Lønnebotn et al. PlosOne 2018[46].

For peer review only - http://bmjopen.bmj.com/site/about/guidelines.xhtml

STRENGTHS AND LIMITATIONS

The main strength of the RHINESSA study is the large number of offspring-parent pairs with rich and similar information from both generations, collected using similar protocols, and with very little missing data on key variables. Both fathers and mothers have been extensively characterised over twenty years of childbearing age, and the availability of such parental exposure information for adolescent and adult offspring is quite unique. The prospectively and retrospectively collected data on family members in this multi-generation study allow validation of information provided about family members[41-46], thereby extending the number of generations that can be analysed in a robust manner. The multi-centre structure is a strength in terms of larger external validity. While the largest number of study participants are from the relatively homogeneous Nordic countries, the Estonian, Spanish and Australian study centres contribute to diversity in the study population improving the external generalisability beyond the Nordic countries. The excellent population- and health registries in the Nordic countries represent a major strength of the study, family members can be identified in an unbiased manner and a wealth of data are available for all generations. For some study centres there is information on five generations, covering birth cohorts born over more than one century – the century when the welfare societies were established in many Western societies.

A weakness of the RHINESSA study is that detailed parental data are mostly available for one parent of the offspring, the parent (mother or the father) participating in the ECRHS and RHINE studies. To meet this challenge, a sub-cohort of the “other” parents has been studied in Bergen RHINESSA, validation studies have been performed to improve the usefulness of information reported by offspring on both parents, and there are registry data available for both parents in North European study centres. Another weakness is the relatively low response rates. Fortunately, exposure information in terms of parental information is available for responders and non-responders. While selection bias cannot be ruled out, it is reassuring that table 4 suggests similar parental characteristics

For peer review only - http://bmjopen.bmj.com/site/about/guidelines.xhtml

for responding and non-responding offspring. In study centres with the appropriate parental consent, information on a number of health outcomes in offspring can be obtained from national registries. Finally, the multi-generation multi-centre study design is challenging with regard to standardization of data collection over time and between generations and study centres, and random heterogeneity in the data due to this may attenuate true results. To face this challenge, we used detailed standard operating procedures and co-ordinated field-worker training, including extensive interview guides and standardised procedures for translations and back-translations of questionnaires and interviews. The use of self-reported data is encumbered with limitations but key in assessment of respiratory symptoms, occupational titles etc.; fortunately the ECRHS tools are widely used and offer important possibilities to compare with other studies.

So, what lessons have we learned from the cohort's creation that could be useful for other researchers? One most useful contribution from RHINESSA to other researchers, is the possibility to validate information provided by family members. In general, we find that strong, longstanding collaboration and friendship has been key for creating a complex set of cohorts in a longitudinal multi-centre setting. Thus, building on existing cohorts with well-functioning researcher networks appears to be important for future multi-generation epidemiological studies.

COLLABORATION

Requests for collaboration with RHINESSA and access to data can be made to the RHINESSA steering committee by PI Professor Cecilie Svanes (cecilie.svanes@uib.no) or vice PI Professor Vivi Schlünssen (vs@ph.au.dk). Reuse of the data must be done in collaboration with the RHINESSA study team, and we in particular encourage collaboration related to multi-generation analyses. Further information including issues on data security and sharing of data and additional study results can be found at www.rhinessa.net.

For peer review only - http://bmjopen.bmj.com/site/about/guidelines.xhtml

CONTRIBUTORS

The Cohort Profile manuscript was written by CS and VS, AJ had particular responsibility for the tables in addition to revision of the text, all co-authors contributed to discussions during the development of the manuscript and revised the manuscript. CS is principal investigator (PI) of the study; VS is vice PI of the study and PI of Arhus study centre; AJ is lead data manager of the study; FGR is PI for the RHINESSA women studies; CJ is PI of the RHINE study; RJB, SCD, BB, LB, MH, NOJ, AM, JMM, AO and JLS are current or previous centre PIs of the study; TG, CL and ST are centre vice PIs.

Contribution to the RHINESSA studies

The RHINESSA study is led by Cecilie Svanes (PI) and Vivi Schlünssen (vice PI), and a **steering committee** that includes study centre PIs Francisco Javier Callejas (Albacete), Randi J Bertelsen (Bergen), Mathias Holm (Göteborg), Jose Luis Sanchez (Huelva), Bryndis Benediktsdottir (Reykjavik), Shyamali Dharmage (Melbourne), Nils Oskar Jõgi (Tartu), Anna Oudin (Umeå), Andrei Malinovschi (Uppsala) and Vivi Schlünssen (Århus), and researchers with specific functions: Christer Janson (PI RHINE), Debbie Jarvis (PI ECRHS), Joachim Heinrich (SC ECRHS), Ane Johannessen (lead data management), Randi Bertelsen (lead biobank), Francisco Gómez Real (lead women's studies), Julia Dratva (children's and adolescent health), Thorarinn Gislason (sleep), Bertil Forsberg (air pollution), Dan Norbäck (indoor environment), Torben Sigsgaard (biodiversity, farm environment), Kjell Torén (occupation and disability). The **co-ordinating centre** in Bergen includes researchers Cecilie Svanes (lead RHINESSA), Randi J Bertelsen (lead biobank), Ane Johannessen (lead data management), Francisco Gomez Real (lead women's studies), and a support team with administrative leader/project coordinator Vilde Marie Svanes Sørbye (2015-d.d) and Eirunn Waatevik Saure (2012-15), assistant project coordinator Benedikte Svanes Sørbye, data managers Bente Sved Skottvoll and Eivind Rebnord, and lead field work Nina Særvold. The RHINESSA Scientific Advisory Board includes Simone Accordini (transgenerational statistical analyses), Jan

For peer review only - http://bmjopen.bmj.com/site/about/guidelines.xhtml

Vilhelm Bakke (the Norwegian Labour inspection), Hogne Skogesal (the Norwegian Asthma and Allergy Association), Karin Lødrup Carlsen (paediatric asthma, children cohorts), Susanne Krauss-Etschmann (transgenerational animal models), Joachim Heinrich (respiratory epidemiology, children cohorts), John Holloway (epigenomics), William Horsnell (immunology, animal models), Deborah Jarvis (PI ECRHS, respiratory epidemiology), Francine Kaufmann (respiratory epidemiology, genetic epidemiology), Torvid Kiserud (foetal medicine), Stephanie London (genetic epidemiology), Benjamin Marsland (airways microbiome), Juha Pekkanen (respiratory epidemiology) and Jordi Sunyer (epidemiology, children cohorts). The related **ECRHS** study is led by Deborah Jarvis and Peter Burney (ECRHS co-ordinating team in London), and the **RHINE** study by Christer Janson (RHINE co-ordinator, Uppsala). Standardisation of procedures in ECRHS has guided standardisation of procedures in the RHINESSA clinical study phases, and standardisation of ECRHS and RHINE questionnaires has guided the RHINESSA questionnaire study phases.

RHINESSA investigators

Albacete: Francisco Javier Callejas (PI), Raúl Godoy (vice PI), Jesus Martínez-Moratalla (former PI). **Bergen**: Randi J Bertelsen (PI), Trude Duelien Skorge (vice PI), Christine Drengenes, Ane Johannessen, Nils Oskar Jõgi, Maryia Khomich, Jorunn Kirkeleit, Toril Mørkve Knudsen, Ingrid Kuiper, Juan Pablo López-Cervantes, Marianne Lønnebotn, Shokouh Makvandi-Nejad, Antonio Pérez, Francisco Gomez Real, Anders Røsland, Rajesh Shigdel, Svein Magne Skulstad, Torgeir Storaas, Cecilie Svanes, Øistein Svanes, Kai Triebner, Gro Tjalvin, Hilde Vindenes, Shanshan Xu. **Göteborg**: Mathias Holm (PI), Kjell Torén. **Huelva**: Jose Luis Sanchez (PI), Jose Maldonado (vice PI). **Reykjavik**: Bryndis Benediktsdottir (PI), Thorarinn Gislason (vice PI). **Melbourne**: Shyamali Dharmage (PI), Caroline Lodge (vice PI), Michael Abramson, Lyle Gurrin, Adrian Lowe, David Martino, Gita Mishra, Jennifer Perret, Bruce Thompson. **Tartu**: Nils Oskar Jõgi (PI), Rain Jõgi (vice PI). **Umeå**: Anna Oudin (PI), Lennart Bräbeck (former PI), Bertil Forsberg (vice PI), Karl Franklin. **Uppsala**: Andrei Malinovschi (PI), Christer Janson (vice PI), Dan Norbäck, Eva Lindberg, Magnus

For peer review only - http://bmjopen.bmj.com/site/about/guidelines.xhtml

Ekstrøm. **Århus**: Vivi Schlünssen (PI), Christine Cramer, Kathrine Pape, Torben Sigsgaard, Signe Timm, Anne Mette Lund Würtz. **Other**: Simone Accordini, Lucia Calciano, Julia Dratva, Joachim Heinrich, John Holloway, William Horsnell, Deborah Jarvis, Susanne Krauss-Etchmann.

COMPETING INTERESTS

CS, AJ, RJB, SCD, BB, LB, TG, MH, NOJ, CL, AM, JMM, AO, JLS, ST, CJ and VS have declared no conflict of interest. SCD holds an Investigator initiated grant funded by Pfizer. FGR has received payment for a conference presentation from AstraAZeneca.

DATA AVAILABILITY STATEMENT

Requests for access to data can be made to the RHINESSA steering committee by PI Professor Cecilie Svanes (cecilie.svanes@uib.no) or vice PI Professor Vivi Schlünssen (vs@ph.au.dk). Reuse of the data must be done in collaboration with the RHINESSA study team. Further information including issues on data security and sharing of data can be found at www.rhinessa.net.

FUNDING

Co-ordination of the RHINESSA study has received funding from the Research Council of Norway (Grants No. 274767, 214123, 228174, 230827 and 273838), ERC StG project BRuSH #804199, the European Union's Horizon 2020 research and innovation programme under grant agreement No. 633212 (the ALEC Study WP2), the Bergen Medical Research Foundation, and the Western Norwegian Regional Health Authorities (Grants No. 912011, 911892 and 911631). Study centres have further received local funding from the following: **Bergen**: the above grants for study establishment and co-ordination, and, in addition, World University Network (REF and Sustainability grants), Norwegian Labour Inspection, the Norwegian Asthma and Allergy Association and Trond Mohn Foundation (Grant ID BFS2017TMT02). **Albacete** and **Huelva**: Sociedad Española de Patología Respiratoria (SEPAR) Fondo de Investigación Sanitaria (FIS PS09). **Gøteborg**, **Umeå** and **Uppsala**: the Swedish Heart and Lung Foundation, the Swedish Asthma and

For peer review only - http://bmjopen.bmj.com/site/about/guidelines.xhtml

Allergy Association. **Reykjavik**: Iceland University. **Melbourne**: Australian National Health and Medical Research Council (NHMRC) Project Grant ID1128450. **Tartu**: the Estonian Research Council (Grant No. PUT562). **Århus**: The Danish Wood Foundation (Grant No. 444508795), the Danish Working Environment Authority (Grant No. 20150067134), Aarhus University (PhD scholarship).

The **RHINE** study received funding by Norwegian Research Council, Norwegian Asthma and Allergy Association, Danish Lung Association, Swedish Heart and Lung Foundation, Vårdal Foundation for Health Care Science and Allergy Research, Swedish Asthma and Allergy Association, Icelandic Research Council, and Estonian Science Foundation. The RHINE IV ongoing study has received funding from the Research Council of Norway project Life-GAP grant No. 300765.

Co-ordination of the ECRHS study has received funding from the European Union's Horizon 2020 research and innovation programme under grant agreement No. 633212 (the ALEC study), the Medical Research Council (ECRHS III) and the European Commission FP5 and FP7 (ECRHS I and II). The ECRHS IV ongoing study in the ten RHINESSA study centres has received funding from the European Union's Horizon 2020 research and innovation programme projects EPHOR under grant agreement No. 874703 and European Research Council (ERC) project BRuSH under grant agreement No. 804199, and from the Research Council of Norway grant No. 273838. Funding agencies for ECRHS I, II and III are reported in the online supplement.

The funding agencies have had no direct role in the conduct of the study or the data collection and management, nor of data analysis or manuscript preparation.

ACKNOWLEDGMENTS

A special thanks to all RHINESSA and RHINE/ECRHS study participants, their contributions are greatly appreciated. We also thank the fieldworkers of all the study centres, in particular Nina

For peer review only - http://bmjopen.bmj.com/site/about/guidelines.xhtml

Særvold, Hilda Andersen and fieldworkers of the Research Unit for Health Surveys, University of Bergen, who had a leading role in the field work of the RHINESSA study.

For peer review only

References

1. Pembrey M, Saffery R, Bygren LO. Human transgenerational responses to early-life experience: potential impact on development, health and biomedical research. *J Med Genet* 2014; **51**: 563-72.
2. Soubry A, Hoyo C, Jirtle RL, Murphy SK. A paternal environmental legacy: evidence for epigenetic inheritance through the male germ line. *Bioessays* 2014; **36**: 359-71.
3. Mørkve Knudsen T, Rezwan FI, Jiang Y, Karmaus W, Svanes C, Holloway JW. Transgenerational and intergenerational epigenetic inheritance in allergic diseases. *J Allergy Clin Immunol* 2018; **142**: 765-72.
4. Jawaid A, Jehle KL, Mansuy IM. Impact of Parental Exposure on Offspring Health in Humans. *Trends Genet* 2021; **37**: 373-88.
5. Golding J, Pembrey M, Iles-Caven Y, Watkins S, Suderman M, Northstone K. Ancestral smoking and developmental outcomes: a review of publications from a population birth cohort†. *Biol Reprod* 2021; **105**: 625-31.
6. Svanes C, Bertelsen RJ, Accordini S, et al. Exposures during the prepuberty period and future offspring's health: evidence from human cohort studies†. *Biol Reprod* 2021; **105**: 667-80.
7. Krauss-Etschmann S, Meyer KF, Dehmel S, Hylkema MN. Inter- and transgenerational epigenetic inheritance: evidence in asthma and COPD? *Clin Epigenetics* 2015; **7**: 53.
8. Nilsson EE, Sadler-Riggleman I, Skinner MK. Environmentally induced epigenetic transgenerational inheritance of disease. *Environ Epigenet* 2018; **4**: dvy016.
9. Heard E, Martienssen RA. Transgenerational epigenetic inheritance: myths and mechanisms. *Cell* 2014; **157**: 95-109.
10. Hammer B, Wagner C, Divac Rankov A, et al. In utero exposure to cigarette smoke and effects across generations: A conference of animals on asthma. *Clin Exp Allergy* 2018; **48**: 1378-90.
11. Pembrey ME, Bygren LO, Kaati G, et al. Sex-specific, male-line transgenerational responses in humans. *European journal of human genetics : EJHG* 2006; **14**: 159-66.
12. Bygren LO, Kaati G, Edvinsson S. Longevity determined by paternal ancestors' nutrition during their slow growth period. *Acta Biotheor* 2001; **49**: 53-9.
13. Li YF, Langholz B, Salam MT, Gilliland FD. Maternal and grandmaternal smoking patterns are associated with early childhood asthma. *Chest* 2005; **127**: 1232-41.
14. Bline AP, Dearfield KL, DeMarini DM, Marchetti F, Yauk CL, Escher J. Heritable hazards of smoking: Applying the "clean sheet" framework to further science and policy. *Environ Mol Mutagen* 2020; **61**: 910-21.
15. Behrman JR, Calderon MC, Preston SH, Hoddinott J, Martorell R, Stein AD. Nutritional supplementation in girls influences the growth of their children: prospective study in Guatemala. *Am J Clin Nutr* 2009; **90**: 1372-9.
16. Burney PG, Luczynska C, Chinn S, Jarvis D. The European Community Respiratory Health Survey. *The European respiratory journal* 1994; **7**: 954-60.
17. Chinn S, Jarvis D, Burney P, et al. Increase in diagnosed asthma but not in symptoms in the European Community Respiratory Health Survey. *Thorax* 2004; **59**: 646-51.
18. Jarvis D, Newson R, Janson C, et al. Prevalence of asthma-like symptoms with ageing. *Thorax* 2018; **73**: 37-48.
19. Johannessen A, Verlato G, Benediktsdottir B, et al. Longterm follow-up in European respiratory health studies - patterns and implications. *BMC Pulm Med* 2014; **14**: 63.
20. Janson C, Johannessen A, Franklin K, et al. Change in the prevalence asthma, rhinitis and respiratory symptom over a 20 year period: associations to year of birth, life style and sleep related symptoms. *BMC Pulm Med* 2018; **18**: 152.

For peer review only

For peer review only - http://bmjopen.bmj.com/site/about/guidelines.xhtml

21. Johannessen A, Lonnebotn M, Calciano L, et al. Being overweight in childhood, puberty, or early adulthood: Changing asthma risk in the next generation? *J Allergy Clin Immunol* 2020; **145**: 791-9 e4.
22. Svanes C, Koplin J, Skulstad SM, et al. Father's environment before conception and asthma risk in his children: a multi-generation analysis of the Respiratory Health In Northern Europe study. *International journal of epidemiology* 2017; **46**: 235-45.
23. Accordini S, Calciano L, Johannessen A, et al. A three-generation study on the association of tobacco smoking with asthma. *International journal of epidemiology* 2018; **47**: 1106-17.
24. Arah OA. Commentary: Tobacco smoking and asthma: multigenerational effects, epigenetics and multilevel causal mediation analysis. *International journal of epidemiology* 2018; **47**: 1117-9.
25. Lutz SM, Thwing A, Schmiege S, et al. Examining the role of unmeasured confounding in mediation analysis with genetic and genomic applications. *BMC Bioinformatics* 2017; **18**: 344.
26. Lederer DJ, Bell SC, Branson RD, et al. Control of Confounding and Reporting of Results in Causal Inference Studies. Guidance for Authors from Editors of Respiratory, Sleep, and Critical Care Journals. *Ann Am Thorac Soc* 2019; **16**: 22-8.
27. Accordini S, Calciano L, Johannessen A, et al. Prenatal and prepubertal exposures to tobacco smoke in men may cause lower lung function in future offspring: a three-generation study using a causal modelling approach. *The European respiratory journal* 2021; **58**.
28. Mørkve Knudsen GT, Rezwan FI, Johannessen A, et al. Epigenome-wide association of father's smoking with offspring DNA methylation: a hypothesis-generating study. *Environ Epigenet* 2019; **5**: dvz023.
29. Hammer B, Kadalayil L, Boateng E, et al. Preconceptional smoking alters spermatozoal miRNAs of murine fathers and affects offspring's body weight. *Int J Obes (Lond)* 2021; **45**: 1623-7.
30. Lønnebotn M, Calciano C, Accordini S, et al. Lung function in adult offspring as associated with their father's overweight in childhood/puberty. *European Respiratory Journal* 2020; **56**: 2066.
31. Knudsen GTM, Dharmage S, Janson C, et al. Parents' smoking onset before conception as related to body mass index and fat mass in adult offspring: Findings from the RHINESSA generation study. *PloS one* 2020; **15**: e0235632.
32. Bertelsen RJ, Rava M, Carsin AE, et al. Clinical markers of asthma and IgE assessed in parents before conception predict asthma and hayfever in the offspring. *Clin Exp Allergy* 2017; **47**: 627-38.
33. Jogi NO, Svanes C, Siiak SP, et al. Zoonotic helminth exposure and risk of allergic diseases: A study of two generations in Norway. *Clin Exp Allergy* 2018; **48**: 66-77.
34. Timm S, Svanes C, Frydenberg M, et al. Does parental farm upbringing influence the risk of asthma in offspring? A three-generation study. *International journal of epidemiology* 2020.
35. Timm S, Frydenberg M, Abramson MJ, et al. Asthma and selective migration from farming environments in a three-generation cohort study. *Eur J Epidemiol* 2019; **34**: 601-9.
36. Pape K, Svanes C, Sejbaek CS, et al. Parental occupational exposure pre- and post-conception and development of asthma in offspring. *International journal of epidemiology* 2020.
37. Tjalvin G, Svanes Ø, Igland J, et al. Maternal preconception occupational exposure to cleaning products and disinfectants and offspring asthma. *J Allergy Clin Immunol* 2022; **149**: 422-31.e5.
38. Kuiper IN, Markevych I, Accordini S, et al. Associations of Preconception Exposure to Air Pollution and Greenness with Offspring Asthma and Hay Fever. *Int J Environ Res Public Health* 2020; **17**.

For peer review only - http://bmjopen.bmj.com/site/about/guidelines.xhtml

39. Lindberg E, Janson C, Johannessen A, et al. Sleep time and sleep-related symptoms across two generations - results of the community-based RHINE and RHINESSA studies. *Sleep Med* 2020; **69**: 8-13.
40. Ekström M, Johannessen A, Abramson MJ, et al. Breathlessness across generations: results from the RHINESSA generation study. *Thorax* 2021.
41. Kuiper IN, Svanes C, Benediktsdottir B, et al. Agreement in reporting of asthma by parents or offspring - the RHINESSA generation study. *BMC Pulm Med* 2018; **18**: 122.
42. Pape K, Svanes C, Malinovschi A, et al. Agreement of offspring-reported parental smoking status: the RHINESSA generation study. *BMC Public Health* 2019; **19**: 94.
43. Timm S, Schlunssen V, Benediktsdottir B, et al. Offspring Reports on Parental Place of Upbringing: Is It Valid? *Epidemiology* 2019; **30**: e16-e8.
44. Skulstad SM, Igland J, Johannessen A, et al. Validation of maternal reported pregnancy and birth characteristics against the Medical Birth Registry of Norway. *PloS one* 2017; **12**: e0181794.
45. Dratva J, Bertelsen R, Janson C, et al. Validation of self-reported figural drawing scales against anthropometric measurements in adults. *Public Health Nutr* 2016; **19**: 1944-51.
46. Lonnebotn M, Svanes C, Igland J, et al. Body silhouettes as a tool to reflect obesity in the past. *PloS one* 2018; **13**: e0195697.

Legend to figures

Figure 1. RHINESSA study concept

The RHINESSA multi-generation study provides data and biomaterial to study how factors in girls and boys, during different age windows, can influence the health of their future children. Factors such as smoking, overweight and air pollution could influence the developing and maturing germ cells in both sexes, and a "fingerprint" of such exposures could be transferred to future offspring and thereby influence their phenotype.

Figure 2. **RHINESSA study design**

The RHINESSA adult offspring cohort (generation 3 "**G3**") includes 8818 young men and women investigated with questionnaires (q), of which 1405 were investigated clinically (c). These are the offspring of men and women participating in the RHINE/ECRHS studies (**G2**) who were followed up over twenty years. In addition, Aarhus, Bergen, Melbourne and Tartu study centres investigated **G3** offspring age 4-17 years (1139q/ 201c), and Bergen study centre investigated **G1** (1470q/145c), the **other G2** parent (910q/152c) and **G4** (750q/433c). In all study centres G3 and G2 study participants provided information about their parents and offspring in G1 and G4.

For peer review only

Supplemental Figure S1. Questionnaire and clinical phases of three study waves of the G2 parents' studies (ECRHS/ RHINE) and the RHINESSA G3 baseline study.

For peer review only

For peer review only - http://bmjopen.bmj.com/site/about/guidelines.xhtml

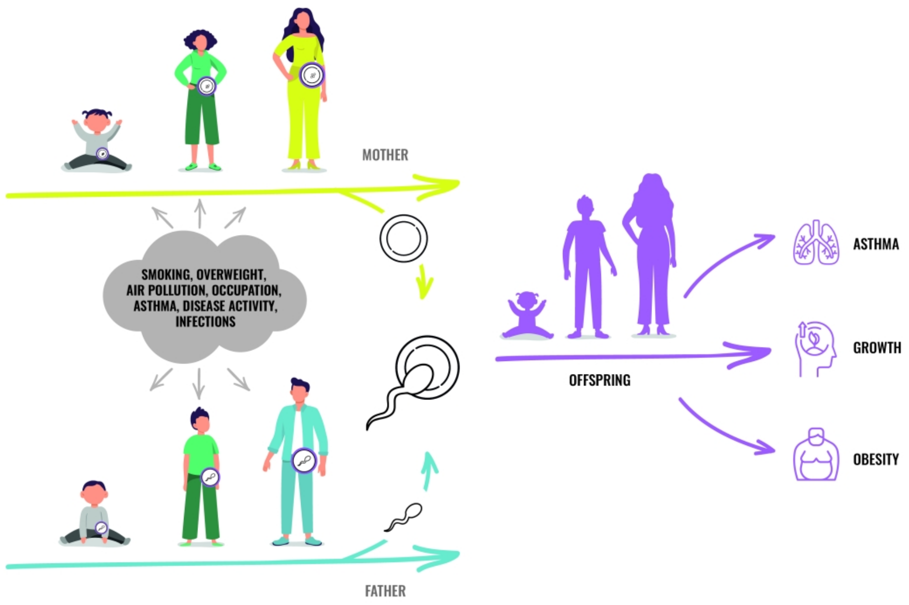

Figure 1. The RHINESSA multi-generation study provides data and biomaterial to study how factors in girls and boys, during different age windows, can influence the health of their future children. Factors such as smoking, overweight and air pollution could influence the developing and maturing germ cells in both sexes, and a "fingerprint" of such exposures could be transferred to future offspring and thereby influence their phenotype.

297x209mm (300 x 300 DPI)

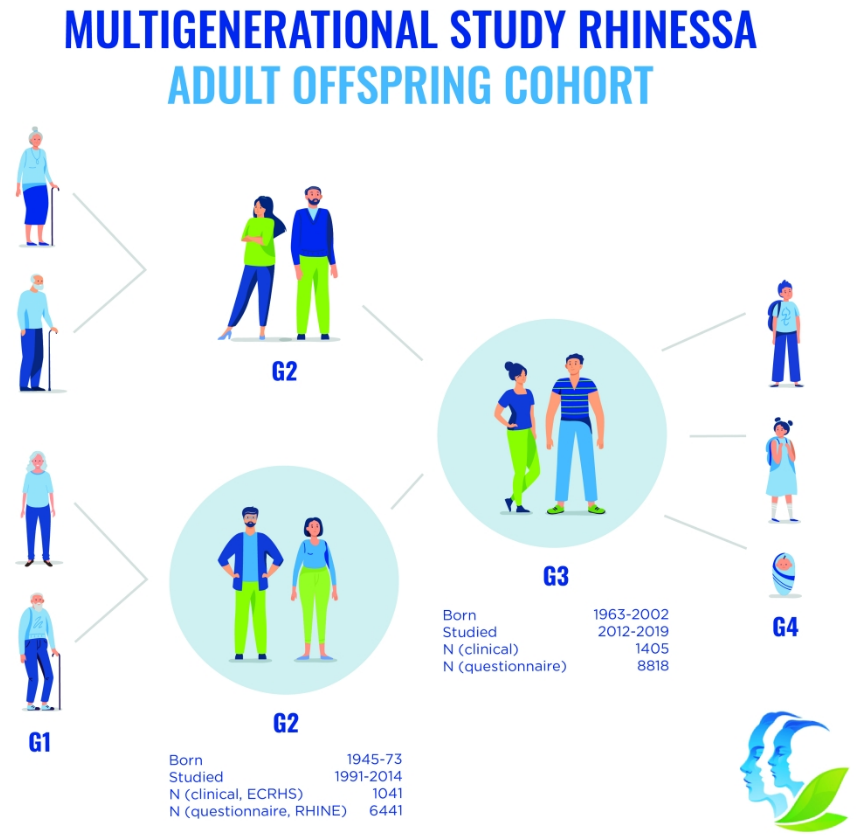

Figure 2. The RHINESSA adult offspring cohort (generation 3 “G3”) includes 8818 young men and women investigated with questionnaires (q), of which 1405 were investigated clinically (c). These are the offspring of men and women participating in the RHINE/ECRHS studies (G2) who were followed up over twenty years. In addition, Aarhus, Bergen, Melbourne and Tartu study centres investigated G3 offspring age 4-17 years (1139q/ 201c), and Bergen study centre investigated G1 (1470q/145c), the other G2 parent (910q/152c) and G4 (750q/433c). In all study centres G3 and G2 study participants provided information about their parents and offspring in G1 and G4.

297x280mm (300 x 300 DPI)

SUPPLEMENTARY MATERIAL

Cohort profile: The multi-generation Respiratory Health in Northern Europe, Spain and Australia (RHINESSA) cohort

Supplementary Table 1. Children and adolescents investigated as part of the RHINESSA G3 study (the adult offspring cohort is the main focus of the paper and not presented here).

	Children (4-9 years)		Adolescents (10-17 years)	
	Questionnaire	Clinical	Questionnaire	Clinical
Aarhus			245	6
Bergen	105	44	571	124
Melbourne	13	0	73	11
Tartu			132	16

Supplementary Table 2A. Bergen all generations

	G1	G2 RHINE/ ECRHS parent	G2 other parent	G3 (all age groups)	G4 (all age groups)
Birth years	1918-65	1947-71	1935-88	1965-2012	1988-2008
Study years	2016-18	1991-2012	2016-18	2012-18	2019-21
Numbers, questionnaire study	1470	3452	910	2440	750
Numbers, clinical study	145	835	152	667	433

Supplementary Table 2B. More details about the Bergen 4th generation cohort

	Children (4-9 years)	Adolescents (10-17 years)	Adults (18+ years)	Total	*Response rate (of total)*
Invited, eligible	436	399	138	973	
Participated questionnaire	379	263	108	750	*77%*
Participated clinical	197	163	73	433	*45%*

For peer review only

SUPPLEMENTARY INFORMATION ON THE EUROPEAN COMMUNITY RESPIRATORY HEALTH SURVEY (ECRHS)

ECRHS I

Co-ordinating Centre (London): P. Burney, S. Chinn, C. Luczynska†, D. Jarvis, E. Lai.

Project Management Group: P. Burney (Project leader-UK), S. Chinn (UK), C. Luczynska† (UK), D. Jarvis (UK), P. Vermeire† (Antwerp), H. Kesteloot (Leuven), J. Bousquet (Montpellier), D. Nowak (Hamburg), J. Prichard† (Dublin), R. de Marco† (Verona), B. Rijcken (Groningen), J.M. Antó (Barcelona), J. Alves (Oporto), G. Boman (Uppsala), N. Nielsen (Copenhagen), P. Paoletti (Pisa).

Financial support: The following grants helped to fund the local studies. **Australia**: Asthma Foundation of Victoria, Allen and Hanbury's; **Belgium**: Belgian Science Policy Office, National Fund for Scientific Research; **Estonia:** Estonian Science Foundation, grant no 1088; **France:** Ministère de la Santé, Glaxo France, Institut Pneumologique d'Aquitaine, Contrat de Plan Etat-Région Languedoc-Rousillon, CNMATS, CNMRT (90MR/10, 91AF/6), Ministre Delegué de la Santé, RNSP; **Germany**: GSF, Bundesminister für Forschung und Technologie; **Italy:** Ministero dell'Università e della Ricerca Scientifica e Tecnologica, CNR, Regione Veneto grant RSF n. 381/05.93; **Norway:** Norwegian Research Council project no. 101422/310; **Spain:** Fondo de Investigación Sanitaria (#91/0016-060-05/E, 92/0319 and #93/0393), Hospital General de Albacete, Hospital General Juan Ramón Jiménez, Dirección Regional de Salud Pública (Consejería de Sanidad del Principado de Asturias), CIRIT (1997 SGR 00079) and Servicio Andaluz de Salud; **Sweden:** The Swedish Medical Research Council, the Swedish Heart Lung Foundation, the Swedish Association against Asthma and Allergy; **Switzerland:** Swiss national Science Foundation grant 4026-28099; **UK:** National Asthma Campaign, British Lung Foundation, Department of Health, South Thames Regional Health Authority.

Coordination: The co-ordination of this work was supported by the European Commission; the authors and participants are grateful to the late C. Baya and M. Hallen for their help during the study, and to K. Vuylsteek and the members of the COMAC for their support.

ECRHS II

Steering Committee: U. Ackermann-Liebrich (University of Basel, Switzerland); N. Kuenzli (University of Basel, and University of Southern California, Los Angeles, USA); J.M. Antó and J. Sunyer (Institut Municipal d' Investigació Médica (IMIM-IMAS), Universitat Pompeu Fabra (UPF), Spain); P. Burney (project leader), S Chinn, D. Jarvis, J. Knox and C. Luczynska (King's College London, UK); I. Cerveri (University of Pavia, Italy); R. de Marco† (University of Verona, Italy); T. Gislason (Iceland University Hospital, Iceland); J. Heinrich and M. Wjst (GSF–Institute of Epidemiology, Germany); C. Janson (Uppsala University, Sweden); B. Leynaert and F. Neukirch

For peer review only

(Institut National de la Santé et de la Recherche Medicale (INSERM), France); J. Schouten (University of Groningen, The Netherlands); C. Svanes (University of Bergen, Norway); P. Vermeire† (University of Antwerp, Belgium).

Principal Investigators and senior scientific teams: **Australia: Melbourne** (M. Abramson, E.H. Walters, J. Raven); **Belgium: South Antwerp and Antwerp City** (P. Vermeire, J. Weyler, M. van Sprundel, V. Nelen); **Estonia: Tartu** (R. Jõgi, A. Soon); **France: Paris** (F. Neukirch, B. Leynaert, R. Liard, M. Zureik), **Grenoble** (I. Pin, J. Ferran-Quentin), **Bordeaux** (A. Taytard, C. Raherison), **Montpellier** (J. Bousquet, P.J. Bousquet); **Germany: Erfurt** (J. Heinrich, M. Wjst, C. Frye, I. Meyer); **Iceland: Reykjavik** (T. Gislason, E. Bjornsson, D. Gislason, K.B. *Jörundsdóttir*); **Italy: Turin** (R. Bono, M. Bugiani, P. Piccioni, E. Caria, A. Carosso, E. Migliore, G. Castiglioni), **Verona** (R. de Marco†, G. Verlato, E. Zanolin, S. Accordini, A. Poli, V. Lo Cascio, M. Ferrari, I. Cazzoletti), **Pavia** (A. Marinoni, S. Villani, M. Ponzio, F. Frigerio, M. Comelli, M. Grassi, I. Cerveri, A. Corsico); **Norway: Bergen** (A. Gulsvik, E. Omenaas, C. Svanes, B. Laerum); **Spain: Albacete** (J. Martinez-Moratalla Rovira, E. Almar, M. Arévalo, C. Boix, G. González, J.M. Ignacio García, J. Solera, J. Damián), **Galdakao** (N. Muñozguren, J. Ramos, I. Urrutia, U. Aguirre), **Barcelona** (J.M. Antó, J. Sunyer, M. Kogevinas, J.P. Zock, X. Basagaña, A. Jaen, F. Burgos, C. Acosta), **Huelva** (J. Maldonado, A. Pereira, J.L. Sanchez), **Oviedo** (F. Payo, I. Huerta, A. de la Vega, L. Palenciano, J. Azofra, A. Cañada); **Sweden**: **Göteborg** (K. Toren, L. Lillienberg, A.C. Olin, B. Balder, A. Pfeifer-Nilsson, R. Sundberg), **Umea** (E. Norrman, M. Soderberg, K.A. Franklin, B. Lundback, B. Forsberg, L. Nystrom), **Uppsala** (C. Janson, G. Boman, D. Norback, G. Wieslander, M. Gunnbjornsdottir); **Switzerland: Basel** (N. Küenzli, B. Dibbert, M. Hazenkamp, M. Brutsche, U. Ackermann-Liebrich); **United Kingdom: Ipswich** (D. Jarvis, R. Hall, D. Seaton), **Norwich** (D. Jarvis, B. Harrison).

Financial Support: **Australia:** National Health and Medical Research Council; **Belgium: Antwerp:** Fund for Scientific Research (G.0402.00), University of Antwerp, Flemish Health Ministry; **Estonia: Tartu:** Estonian Science Foundation grant no 4350; **France: (All)** Programme Hospitalier de Recherche Clinique—Direction de la Recherche Clinique (DRC) de Grenoble 2000 number 2610, Ministry of Health, Ministère de l'Emploi et de la Solidarité, Direction Génerale de la Santé, Centre Hospitalier Universitaire (CHU) de Grenoble; **Bordeaux:** Institut Pneumologique d'Aquitaine; **Grenoble:** Comité des Maladies Respiratoires de l'Isere; **Montpellier:** Aventis (France), Direction Regionale des Affaires Sanitaires et Sociales Languedoc-Roussillon; **Paris:** Union Chimique Belge-Pharma (France), Aventis (France), Glaxo France; **Germany: Erfurt:** GSF—National Research Centre for Environment and Health, Deutsche Forschungsgemeinschaft (FR1526/1-1); **Hamburg:** GSF—National Research Centre for Environment and Health, Deutsche Forschungsgemeinschaft (MA 711/4-1); **Iceland: Reykjavik:** Icelandic Research Council, Icelandic University Hospital Fund; **Italy: Pavia:** GlaxoSmithKline Italy, Italian Ministry of University and Scientific and Technological Research (MURST), Local University Funding for Research 1998 and 1999; **Turin:** Azienda Sanitaria Locale 4 Regione Piemonte (Italy), Azienda Ospedaliera Centro Traumatologico Ospedaliero/Centro Traumatologico Ortopedico—Istituto Clinico Ortopedico Regina Maria Adelaide Regione Piemonte; **Verona:** Ministero dell'Università e della Ricerca Scientifica (MURST), Glaxo Wellcome spa; **Norway: Bergen:** Norwegian Research Council, Norwegian Asthma and Allergy Association, Glaxo Wellcome AS, Norway Research Fund; **Spain: Albacete:** Fondo de Investigaciones Sanitarias (97/0035-01, 99/0034-01, and 99/0034-02), Hospital Universitario de Albacete, Consejeria de Sanidad; **Barcelona:** Sociedad Espanola de Neumología y Cirugía Toracica,

For peer review only

Public Health Service (R01 HL62633-01), Fondo de Investigaciones Sanitarias (97/0035-01, 99/0034-01, and 99/0034-02), Consell Interdepartamental de Recerca i Innovació Tecnològica (1999SGR 00241), Instituto de Salud Carlos III, Red de Centros de Epidemiología y Salud Pública (C03/09), Red de Bases moleculares y fisiológicas de las Enfermedades Respiratorias (C03/011), Red de Grupos Infancia y Medio Ambiente (G03/176); **Huelva:** Fondo de Investigaciones Sanitarias (97/0035-01, 99/0034-01, and 99/0034-02); **Galdakao:** Basque Health Department; **Oviedo:** Fondo de Investigaciones Sanitarias (97/0035-02, 97/0035, 99/0034-01, 99/0034-02, 99/0034-04, 99/0034-06, 99/350, and 99/0034-07), European Commission (EU-PEAL PL01237), Generalitat de Catalunya (CIRIT 1999 SGR 00214), Hospital Universitario de Albacete, Sociedad Española de Neumología y Cirugía Torácica (SEPAR R01 HL62633-01), Red de Centros de Epidemiología y Salud Pública (C03/09), Red de Bases moleculares y fisiológicas de las Enfermedades Respiratorias (C03/011), Red de Grupos Infancia y Medio Ambiente (G03/176, 97/0035-01, 99/0034-01, and 99/0034-02); **Sweden: Göteborg, Umea, and Uppsala**: Swedish Heart Lung Foundation, Swedish Foundation for Health Care Sciences and Allergy Research, Swedish Asthma and Allergy Foundation, Swedish Cancer and Allergy Foundation, Swedish Council for Working Life and Social Research (FAS); **Switzerland: Basel:** Swiss National Science Foundation, Swiss Federal Office for Education and Science, Swiss National Accident Insurance Fund; **UK: Ipswich and Norwich**: Asthma UK (formerly known as National Asthma Campaign).

Cordination: The coordination of this work was supported by the European Commission, as part of their Quality of Life programme (QLK4-CT-1999-01237).

ECRHS III

Principal Investigators and senior scientific teams: **Australia: Melbourne** (M. Abramson, G. Benke, S. Dharmage, B. Thompson, S. Kaushik, M. Matheson). **Belgium: South Antwerp & Antwerp City** (J. Weyler, H. Bentouhami, V. Nelen). **Estonia: Tartu** (R. Jõgi, H.Orru). **France: Bordeaux** (C. Raherison, P.O. Girodet), **Grenoble** (I. Pin, V. Siroux, J. Ferran, J.L. Cracowski), **Montpellier** (P. Demoly, A. Bourdin, I. Vachier), **Paris** (B. Leynaert, D. Soussan, D. Courbon, C. Neukirch, L. Alavoine, X. Duval, I. Poirier). **Germany: Erfurt** (J. Heinrich, E. Becker, G. Woelke, O. Manuwald), **Hamburg** (H. Magnussen, D. Nowak, A-M. Kirsten). **Iceland: Reykjavik** (T. Gislason, B. Benediktsdottir, D. Gislason, E.S. Arnardottir, M. Clausen, G. Gudmundsson, L. Gudmundsdottir, H. Palsdottir, K. Olafsdottir, S. Sigmundsdottir, K. Bara-Jörundsdottir). **Italy**: **Pavia** (I. Cerveri, A. Corsico, A. Grosso, F. Albicini, E. Gini, E.M. Di Vincenzo, V. Ronzoni, S. Villani, F. Campanella, M. Gnesi, F. Manzoni, L. Rossi, O. Ferraro), **Turin** (M. Bugiani, R. Bono, P. Piccioni, R. Tassinari, V. Bellisario, G. Trucco), **Verona** (R. de Marco†, S. Accordini, L. Calciano, L. Cazzoletti, M. Ferrari, A.M. Fratta Pasini, F. Locatelli, P. Marchetti, A. Marcon, E. Montoli, G. Nguyen, M. Olivieri, C. Papadopoulou, C. Posenato, G. Pesce, P. Vallerio, G. Verlato, E. Zanolin). **Norway: Bergen** (C. Svanes, E. Omenaas, A. Johannessen, T. Skorge, F. Gomez Real). **Spain: Albacete** (J. Martinez-Moratalla Rovira, E. Almar, A. Mateos, S. García, A. Núñez, P. López, R. Sánchez, E. Mancebo), **Barcelona** (J.M. Antó, J.P. Zock, J. Garcia-Aymerich, M. Kogevinas, X. Basagaña, A.E. Carsin, F. Burgos, C. Sanjuas, S. Guerra, B. Jacquemin, P. Davdand), **Galdakao** (N. Muñozguren, I. Urrutia, U. Aguirre, S. Pascual), **Huelva** (J. Antonio Maldonado, A. Pereira, J. Luis

Sánchez, L. Palacios), **Oviedo** (F. Payo, I. Huerta, N. Sánchez, M. Fernández, B. Robles). **Sweden: Göteborg** (K. Torén, M. Holm, J-L. Kim, A-C. Olin, A. Dahlman-Höglund), **Umea** (B. Forsberg, L. Braback, L. Modig, B. Järvholm, H. Bertilsson, K.A. Franklin, C. Wahlgreen), **Uppsala** (B. Andersson, D. Norback, U. Spetz Nystrom, G. Wieslander, G.M. Bodinaa Lund, K. Nisser). **Switzerland: Basel** (N.M. Probst-Hensch, N. Künzli, D. Stolz, C. Schindler, T. Rochat, J.M. Gaspoz, E. Zemp Stutz, M. Adam, C. Autenrieth, I. Curjuric, J. Dratva, A. Di Pasquale, R. Ducret-Stich, E. Fischer, L. Grize, A. Hensel, D. Keidel, A. Kumar, M. Imboden, N. Maire, A. Mehta, H. Phuleria, M. Ragettli, M. Ritter, E. Schaffner, G.A. Thun, A. Ineichen, T. Schikowski, M. Tarantino, M. Tsai). **UK: London** (P. Burney, D. Jarvis, S. Kapur, R. Newson, J. Potts), **Ipswich** (N. Innes), **Norwich** (A. Wilson).

Financial Support: **Australia:** National Health & Medical Research Council; **Belgium: Antwerp South, Antwerp City:** Research Foundation Flanders (FWO) (G.0.410.08.N.10); **Estonia: Tartu:** Estonian Ministry of Education (SF0180060s09); **France:** (**All centres**) Ministère de la Santé, Programme Hospitalier de Recherche Clinique (PHRC) national 2010; **Bordeaux:** INSERM U897 Université Bordeaux segalen; **Grenoble:** Comite Scientifique AGIRadom 2011; **Paris:** Agence Nationale de la Santé, Région Ile de France, domaine d'intérêt majeur (DIM); **Germany: Erfurt:** German Research Foundation (HE 3294/10-1); **Hamburg:** German Research Foundation (MA 711/6-1, NO 262/7-1); **Iceland: Reykjavik:** The Landspitali University Hospital Research Fund, University of Iceland Research Fund, ResMed Foundation, California, USA, Orkuveita Reykjavikur (Geothermal plant), Vegagerðin (The Icelandic Road Administration (ICERA). **Italy: (All centres)** Italian Ministry of Health, Chiesi Farmaceutici SpA; **Verona:** Cariverona foundation, Education Ministry (MIUR). **Norway:** Norwegian Research council (214123), Western Norway Regional Health Authorities (911631), Bergen Medical Research Foundation. **Spain:** Fondo de Investigación Sanitaria (PS09/02457, PS09/00716, 09/01511, PS09/02185, PS09/03190), Servicio Andaluz de Salud, Sociedad Española de Neumología y Cirurgía Torácica (SEPAR 1001/2010), Fondo de Investigación Sanitaria (PS09/02457); **Barcelona:** Fondo de Investigación Sanitaria (FIS PS09/00716); **Galdakao:** Fondo de Investigación Sanitaria (FIS 09/01511); **Huelva:** Fondo de Investigación Sanitaria (FIS PS09/02185), Servicio Andaluz de Salud; **Oviedo:** Fondo de Investigación Sanitaria (FIS PS09/03190). **Sweden: (All centres)** The Swedish Heart and Lung Foundation, The Swedish Asthma and Allergy Association, The Swedish Association against Lung and Heart Disease, Swedish Research Council for health, working life and welfare (FORTE); **Göteborg:** Swedish Council for Working life and Social Research; **Umea:** Vasterbotten Country Council ALF grant. **Switzerland:** The Swiss National Science Foundation (33CSCO-134276/1, 33CSCO-108796, 3247BO-104283, 3247BO-104288, 3247BO-104284, 3247-065896, 3100-059302, 3200-052720, 3200-042532, 4026-028099), The Federal office for forest, environment and landscape, The Federal Office of Public Health, The Federal Office of Roads and Transport, the canton's government of Aargan, Basel-Stadt, Basel-Land, Geneva, Luzern, Ticino, Valais, and Zürich, the Swiss Lung League, the canton's Lung League of Basel Stadt/Basel, Landschaft, Geneva, Ticino, Valais, and Zurich, SUVA, Freiwillige Akademische Gesellschaft, UBS Wealth Foundation, Talecris Biotherapeutics GmbH, Abbott Diagnostics, European Commission (018996) (GABRIEL), Wellcome Trust (WT 084703MA). **UK:** Medical Research Council (92091). Support also provided by the National Institute for Health Research through the Primary Care Research Network.

For peer review only

Coordination: The coordination was funded through the Medical Research Council (92091).

† deceased

Ethics approval: Ethics approval was obtained by all centres from the appropriate ethics committees: **Antwerp City and Antwerp South:** Adviescommissie Medische Ethiek UZA-UA (CME); **Tartu:** Research Ethics Committee of the University of Tartu, Estland (N° 209T-17); **French centres:** Comite de protection des personnes, Sud V Est (N° 2011-A00013-38); **German centres:** Ethik-Kommission der Bayerischen Landesarztekammer (N° 10015); **Reykjavik:** National Biotecs Committe of Iceland (NBCI) (N° VSNb2011090016/03.11); **Pavia:** Fondazione IRCCS Policlinico 'San Matteo' (N° P-20110024215); **Turin:** Comitato Etico dell'Azienda Sanitaria Locale TO/2 di Torino (N° 569/09/08); **Verona:** Comitato Etico per la Sperimentazione dell'Azienda Ospedaliera Istituti Ospitalieri di Verona (N° 1393); **Bergen:** Universitetet i Bergen, Regional komité for medisinsk og helsefaglig forskningsetikk, Vest-Norge (REK Vest) (N° 2010/759); **Albacete:** Comité de Ética e Investigación de Complejo Hospitalario de Albacete (N° 04/09); **Barcelona:** Comité Ético de Investigación Clínica del Instituto Municipal de Asistencia Sanitaria, Barcelona, Spain (N° PS09/00716); **Galdakao:** Comité Éticode Investigación del Hospital de Galdakao, Spain (N° 20101104); **Huelva:** Comisión de Investigación del Hospital Juan Ramón Jiménez de Huelva (N° 20090417); **Oviedo:** Comité Ético de Investigación Clínica Regional, Hospital Universitario Central de Asturias (N° 20110415); **Swedish centres:** Ethics Committee at the Medical Faculty, Uppsala University (N° 1999/313 and 2010/068); **Basel:** Swiss Academy of Medical Sciences and the ethics committee of Basel (N° PV123/00,157/00); **UK centres:** NRES Committee London - Stanmore (REC Reference 11/LO/0965 IRAS number 70769).

For peer review only

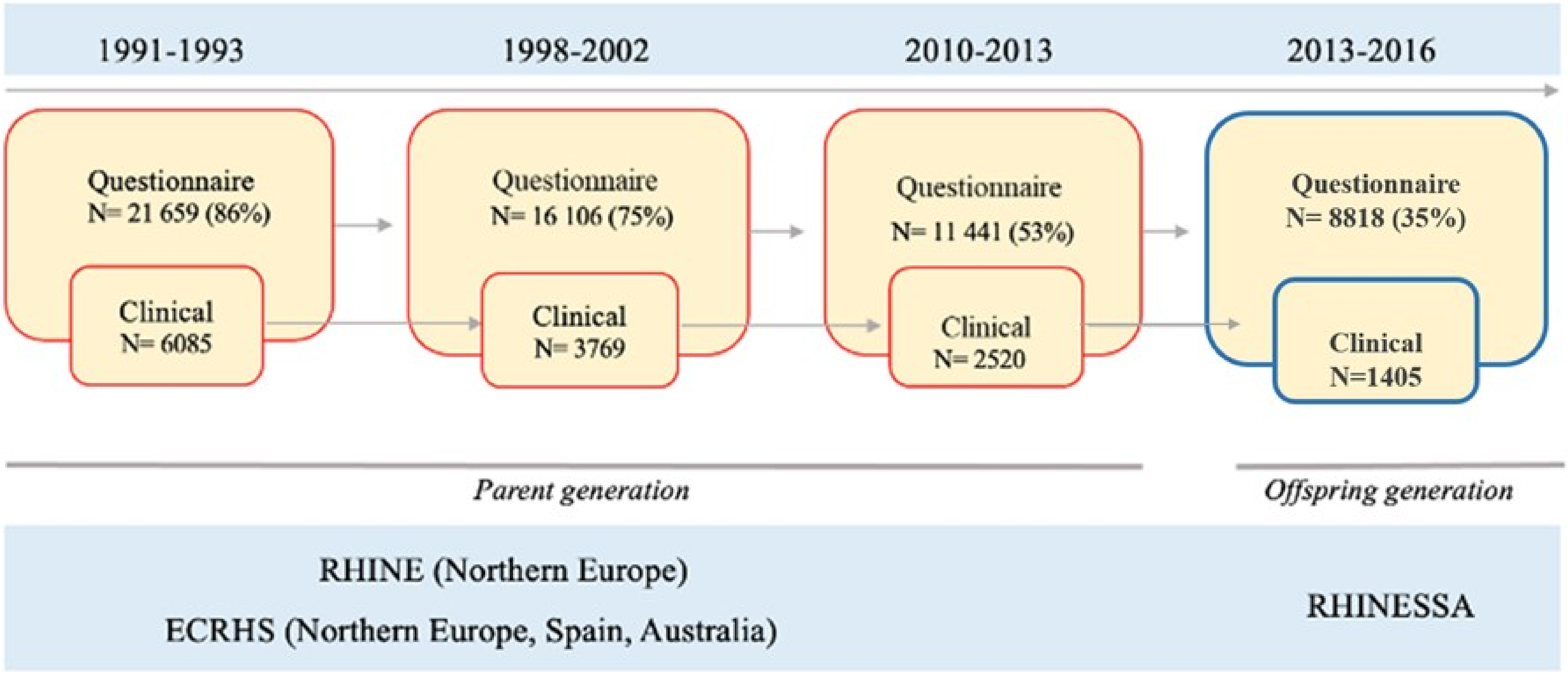
1991-1993
1998-2002
2010-2013
2013-2016
Questionnaire
N= 21 659 (86%)
Clinical
N= 6085
Questionnaire
N= 16 106 (75%)
Clinical
N= 3769
Questionnaire
N= 11 441 (53%)
Clinical
N= 2520
Questionnaire
N= 8818 (35%)
Clinical
N=1405
Parent generation
Offspring generation
RHINE (Northern Europe)
ECRHS (Northern Europe, Spain, Australia)
RHINESSA
